# Synthesis of α,ε-*N*,*N*′-Di-stearoyl Lysine-Derived Amide Lipids and Their Application to Liposome Formulation: Incorporation of Lipid A-Ligand for Bacterial Targeting and Sialic Acid for Phagocytosis Resistance

**DOI:** 10.3390/ijms26189176

**Published:** 2025-09-19

**Authors:** Dean Williams, Alyssa McAdorey, Eric Lei, Greg Beaudoin, Binbing Ling, Debbie Callaghan, Dorothy Fatehi, Angie Verner, Jacqueline Slinn, Maria Moreno, Umar Iqbal, Hui Qian, Hongbin Yan, Wangxue Chen, Wei Zou

**Affiliations:** 1 Human Health Therapeutics Research Centre, National Research Council Canada, 100 Sussex Drive, Ottawa, ON K1A 0R6, Canadadorothy.fatehi@nrc-cnrc.gc.ca (D.F.);; 2 Department of Chemistry and Centre for Biotechnology, Brock University, 1812 Sir Isaac Brock Way, St. Catharines, ON L2S 3A1, Canada; 3 Quantum and Nanotechnologies Research Centre, National Research Council Canada, 11421 Saskatchewan Drive NW, Edmonton, AB T6G 2M9, Canada

**Keywords:** amide lipids, sialic acid, liposomes, targeted delivery, phagocytosis, fluorescent imaging, biodistribution

## Abstract

As part of an antimicrobial resistance (AMR) strategy, we have prepared α,ε-*N*,*N*′-di-stearoyl lysine-based amide lipids to improve the chemical and biological stabilities of nanoparticles. Those amide lipids incorporated a variety of head groups, including lipid A-binding ligand (polymyxin B nonapeptide, PMBN) for bacterial targeting and sialic acid as an alternative to PEGylation for phagocytosis resistance. The study demonstrated that the PMBN-liposome specifically targeted lipid A-containing Gram-negative *Acinetobacter baumannii* bacteria, but not Gram-positive *Staphylococcus aureus*. However, such interaction was interrupted by the adsorption of serum proteins onto liposomes, demonstrating the complexity and challenge of targeted delivery. As expected, slower uptake of sialic acid-liposomes by human leukemia monocytic THP-1 cells was observed, suggesting their resistance to phagocytosis. Additionally, in a mouse model, the sialic acid-containing liposomes showed more favorable biodistribution and longer retention time than the comparable phospholipid-only liposomes. We observed that both sialic acid-incorporated and PEGylated liposomes distributed over the whole mouse bodies and remained for over 48 h. In contrast, the phospholipid-only liposomes rapidly migrated to the liver (5–15 min). In conclusion, although this study did not achieve bacteria-targeted liposome delivery, it provided evidence that the sialic acid-amide lipid can serve as an alternative to PEGylation in future nanomedicine.

## 1. Introduction

The FDA has approved a number of nanomedicines, including liposomes and lipid nanoparticles (LNPs) [1]. Liposomes and LNPs enhance drug deliverability and pharmacokinetics [2,3] by protecting encapsulated payloads from chemical and enzymatic degradation, thereby reducing drug toxicity [4,5]. However, there are still two significant challenges remaining: (1) rapid clearance by the reticuloendothelial system (RES) due to serum protein adsorption [2,6,7] and (2) more efficient targeted delivery. The first challenge has been addressed by the incorporation of PEGylated lipid into liposomal formulations. Polyethylene glycol (PEG) acts as a coating agent to minimize protein adsorption and prolong circulation time [8]. However, PEGylated nanoparticles can still interact with macrophages and other immune cells, leading to the production of anti-PEG antibodies [9]. Recent reports have revealed that SARS-CoV-2 LNP-based mRNA vaccines induce or boost anti-PEG antibodies in humans, likely due to their PEGylated lipid components [10,11,12]. The observation poses a significant challenge to the application of PEGylated nanomedicine, as PEGylated agents are now widely used not only in medicines but also in other commercial products. Additionally, PEGylated nanoparticles may cause severe allergic reactions and anaphylaxis in a small number of patients [13], suggesting nanoparticle PEGylation may not be a long-term solution to overcome the immunological barriers of nanomedicine. To avoid interaction with pre-existing anti-PEG antibodies, alternative approaches included the use of cleavable PEG [14] and other substitutes such as poly(2-methacryloyloxyethyl) phosphorylcholine [15]. Human glycans, such as Gb3 and LacCer [16], could also be attractive replacements, as they are hydrophilic and, in many cases, poorly immunogenic. Since bacterial capsules play a critical role in evading the innate immune system [17], it is conceivable that liposomes could be modified with non-immunogenic carbohydrates to extend their circulation time. Another alternative to PEGylation is to optimize the surface properties of liposomes to allow serum albumin to be favorably adsorbed over opsonin, which could also stabilize the liposomes, resulting in decreased cellular uptake and extended circulation time [18,19,20]. It has been observed that opsonin interacts more favorably with positively charged nanoparticles than those with a negative charge; thus, neutral and negatively charged nanoparticles exhibit longer circulation times [20,21]. In comparison, the second challenge in the targeted delivery of nanomedicine is more difficult and has been attempted by incorporating molecular ligands [22,23], which must be dealt with individually due to the lack of effective common receptors in different cancers and bacterial pathogens. Additionally, improvement of chemical stability is also desired as unsaturated phospholipids can be oxidized, and the ester linkages in the formulation could be hydrolysized [24]. It remains a challenge to formulate phospholipid liposomes with both biological and chemical stability [25,26]. As part of our solution to antimicrobial resistance, the ideal AMR liposomes would have a surface coating other than PEGylation to enhance circulation time, a ligand capable of interacting with a broad spectrum of bacteria, and more stable lipid components. Given that ceramides are an important component of the cell membrane [27], we reasoned that synthetic amide lipids are likely to be biocompatible, more stable than phospholipids, yet still biodegradable, which could make them a potential substitute for phospholipids. Therefore, we synthesized various α,ε-*N*,*N*′-di-stearoyl lysine-derived amide lipids, in which the head groups include a negatively charged sialic acid, a neutral PEGylated entity, and a lipid A-specific ligand (PMBN). In this study, we also investigate whether the PMBN-incorporated liposome can specifically target Gram-negative bacteria, such as *Acinetobacter baumannii*, and the impact of serum protein adsorption on this interaction. Additionally, we assessed the phagocytosis of the liposomes by measuring their uptake by THP-1 cells. Finally, we validated in a mouse model that sialic acid-modified liposomes could be an alternative to PEGylation by measuring their biodistribution and retention time.

## 2. Results and Discussion

### 2.1. Synthesis of Amide Lipids

**Scheme 1:**
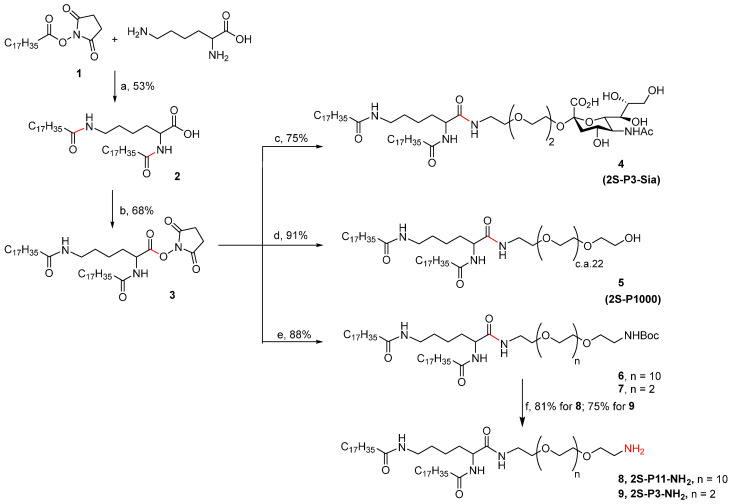
Amide lipid synthesis. Reagents and conditions: (**a**) DMF-Pyridine-TFA (2:1:0.1), reflux, overnight; (**b**) NHS, DCC, 50–60 °C for 3 h then rt overnight; (**c**) Sia-PEG3-NH_2_, in CHCl_3_-DMF, overnight; (**d**) P1000-amine, in CHCl_3_, rt, overnight; (**e**) BocNH-PEG11-amine or BocNH-PEG3-amine, in CHCl_3_, rt, overnight; (**f**) DCM:water:TFA (3:1:1), reflux, 6 h. The reaction sites are highlighted with red color.

Phospholipid liposomes adsorb serum opsonin, triggering a host immune response and facilitating rapid clearance [28], and the lipids also degrade as part of the metabolic process [29]. Therefore, alternative lipids are needed to enhance the immunological, biological, and chemical stabilities of liposomes. Fatty acid amide lipids can be such candidates, in which the fatty acid esters are replaced with amides, and various hydrophilic moieties substitute the phosphates. We used lysine as a surrogate of glycerol, in which both α,ε-amino groups are condensed with fatty acid to form *N*,*N*′-di-amides, and its remaining carboxylic group is linked to a hydrophilic moiety through another amide linkage, as illustrated in Scheme 1. In brief, stearic acid was first converted into an activated ester (**1**) by coupling its carboxylic acid with NHS in the presence of DCC [30]. Further reaction of **1** with lysine met some challenges due to the difficulty in finding a compatible solvent. Eventually, a trinary solvent of pyridine-TFA-DMF was able to dissolve both hydrophobic ester and hydrophilic lysine, which allowed the reaction between **1** and lysine to afford α,ε-*N*,*N*′-di-stearoyl lysine **2**. The purification was simply accomplished by the addition of water to the reaction mixture to precipitate esters (amide lipid **2** and unreacted ester **1**), which were then washed with ethyl acetate to remove residue **1** and retain the product (**2**). Pure **2** was obtained by recrystallization from anhydrous ethanol. Similar to activated ester **1**, ester **3** was prepared from NHS and **2**, and again, the product was purified by recrystallization from anhydrous ethanol. The activated ester **3** was then reacted with various amines to introduce hydrophilic head groups, including sialic acid, amino PEG, and a neutral PEG (1K), to yield respective amide lipids **4** (**2S-P3-Sia**) and **5** (**2S-P1000**) directly, and **8** and **9** (**2S-P11-NH_2_** and **2S-P3-NH_2_**) through *N*-Boc protected intermediates **6** and **7**, respectively. Under proper conditions, amide lipids **4** and **8** would form liposomes carrying either negative or positive surface charges, whereas liposomes from **5** would remain neutral.

**Scheme 2:**
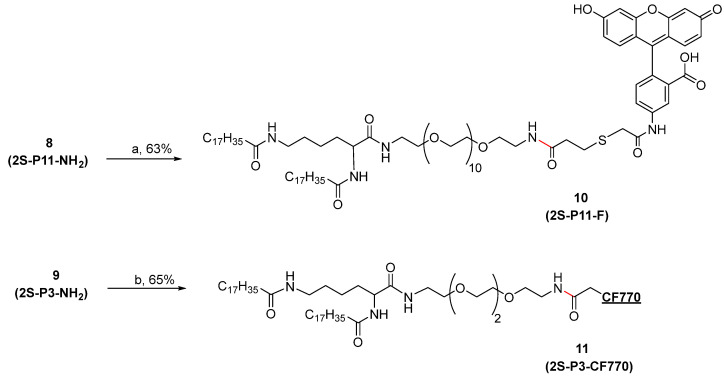
Synthesis of amide lipids for fluorescent imaging. Reagent and conditions: (**a**) fluorescein-5-EX-NHS/DMSO to **8** in CHCl_3_, rt, overnight; (**b**) CF770-NHS/DMF to **8** in CHCl_3_, rt, overnight. The reaction sites are highlighted with red color.

Two additional amide lipids, namely, **2S-P11-F** (**10**) from **8** with a fluorescein tag and **2S-P3-CF770** (**11**) with a near-infrared dye CF770, were also synthesized from **9** (Scheme 2). Both lipids are co-formulated with other synthetic or commercial lipids as fluorescent probes in assays. **2S-P11-F** labeled liposomes were used in liposome-bacteria binding studies and in vitro cellular uptake assays [31], whereas **2S-P3-CF770** was used for in vivo imaging to evaluate liposome biodistribution and retention in mice [32].

**Scheme 3:**
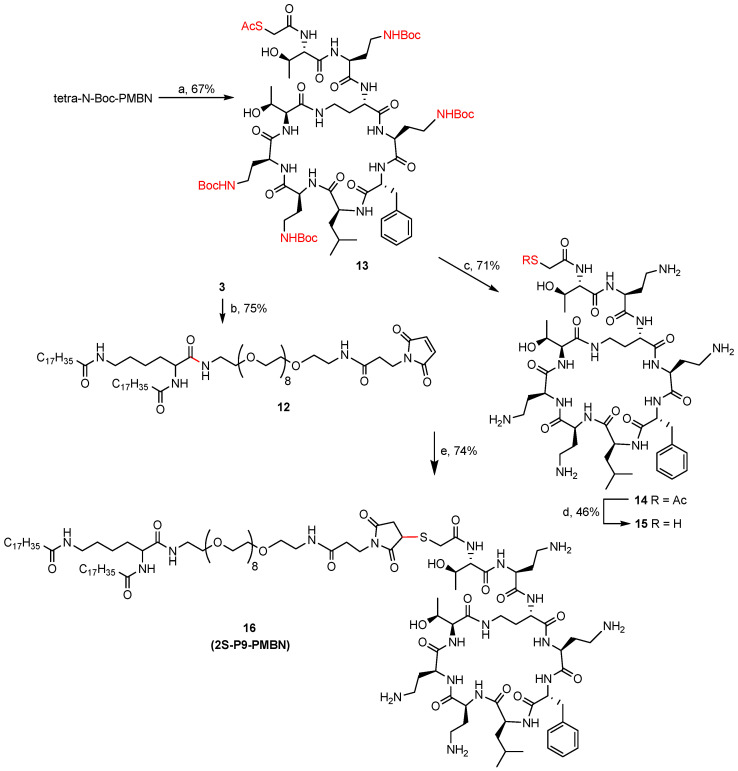
Synthesis of amide lipid with a ligand specific to lipid A. (**a**) *N*-succinimidyl *S*-acetylthioacetate (SATA) in DMSO, r.t. 1 h; (**b**) Mal-PEG9-amine in CHCl_3_, rt, overnight; (**c**) DCM:water:TFA (3:1:1 *v*/*v*), reflux, overnight; (**d**) hydroxylamine, phosphate buffer, pH 7.5, rt, 1.5 h; (**e**) CHCl_3_/MeOH, rt, overnight. The reaction sites are highlighted with red color.

One of the key considerations in developing novel nanomedicines for combating antimicrobial resistance is the incorporation of a ligand for targeted delivery. We selected PMBN, a lipid A-binding cyclopeptide derived from polymyxin B, for Gram-negative bacterial targeting (Scheme 3). Polymyxins are considered last-resort antibiotics for treating Gram-negative bacterial infections due to their significant renal and neurotoxicities [33]. The bactericidal activity of polymyxins depends on its fatty acyl chain interacting with the bacterial lipid domain [34], as PMBN alone is not antibacterial [35]. The low eukaryotic cytotoxicity of PMBN has been exploited for immune targeting; for example, PMBN was conjugated to dinitrophenol, a hapten, through a PEG linker. The conjugate was able to recruit antibodies through the hapten at one end and to bind Gram-negative bacteria by PMBN at the other end, leading to antibody-mediated bacterial killing [36]. Since it has been demonstrated that the binding affinity of multiple interactions is far greater than that of a one-on-one interaction [37], liposomes displaying numerous copies of PMBN, as presented here, could significantly enhance their binding to Gram-negative bacteria and potentially achieve more effective payload delivery [38].

Accordingly, we synthesized an amide lipid with PMBN as the hydrophilic head from commercially available polymyxin B sulfate. First, it was treated with a papain protease to remove the *N*-terminal fatty acyl group and the diaminobutyric acid (Dab) residue [39]. The product was then converted to a tetra-*N*-Boc-PMBN by selective protection of the amino groups of four Dab residues but not the *N*-terminal a-amino group [40]. Next, the free amine was reacted with SATA to give **13** (Scheme 3). Finally, the removal of protecting groups (*N*-Boc and *S*-Ac) from **13** produced **14** and thiol **15**, respectively. Meanwhile, amide lipid **12** with a maleimide was prepared from ester **3** and maleimide PEG9-amine. Coupling of maleimide **12** with thiol **15** afforded **16** (**2S-P9-PMBN**), an amide lipid with PMBN ligand.

### 2.2. Liposome Formulations

**Figure 1:**
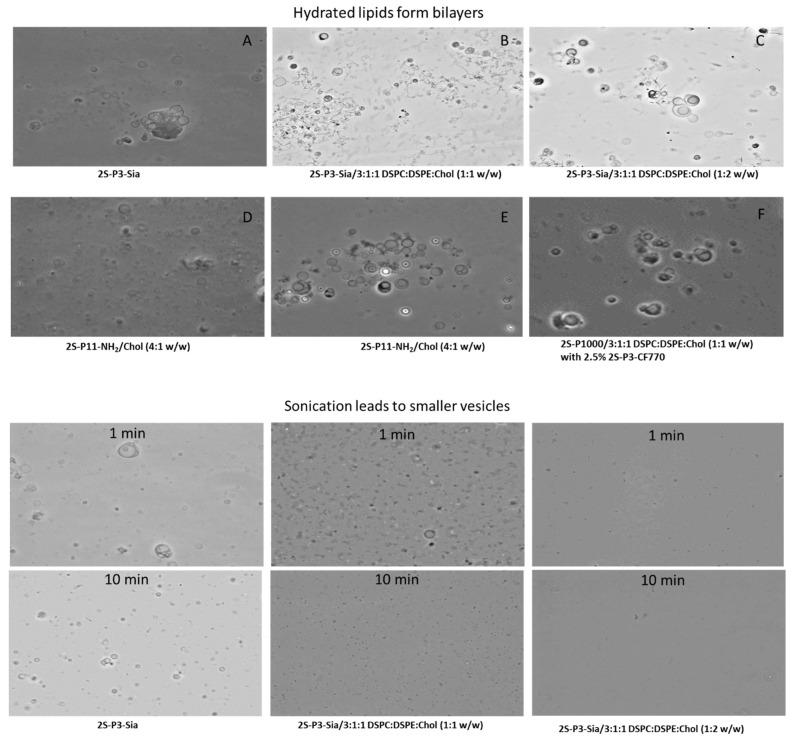
Visualization of hydrated amide lipids and vesicles by light microscope: bilayer vesicles were formed by hydration of amide lipid **2S-P3-Sia** (**A**) or its phospholipids co-formulations (**B**,**C**) at 65 °C in 20 mM sodium phosphate buffer (pH 7.2); **2S-P11-NH_2_/cholesterol** (4:1 *w*/*w*) hydrated at 70 °C in monobasic phosphate (pH 4.5) (**D**) and 10 mM sodium acetate buffer (pH 5.0) (**E**); hydration of **2S-P1000** and phospholipids/cholesterol with 2.5% **2S-P3-CF770** at 65 °C in 20 mM sodium phosphate buffer (pH 7.2) (**F**). Smaller vesicles formed after sonication, and their size appears to be influenced by the **2S-P3-Sia** content.

Liposomes can be formulated through various methods [41,42], which leads to different sizes, structures, encapsulation payloads, and stability. The common method requires lipids to be soluble in diethyl ether or ethanol, which is then mixed, by rapid injection, with an aqueous buffer. The amide lipids we synthesized, however, dissolve only in chloroform or in a mixture of chloroform and methanol, which limited our choices. With the thin film method, the lipid films formed after removal of organic solvent were hydrated in buffer. The microscopic images of several hydrated lipids are shown in Figure 1, and detailed formulation conditions are presented in Table 1.

As expected, hydration of **2S-P3-Sia** in 20 mM phosphate buffer at pH 7.2 resulted in the formation of large-sized bilayer vesicles. Similar results were also observed when **2S-P3-Sia** co-formulated with phospholipids, e.g., DSPC:DSPE:Chol (3:1:1) as shown in Figure 1A–C. The sizes of those vesicles became smaller after sonication. However, **2S-P3-Sia** in the formulation apparently played a role in vesicle stability, as the vesicles formed from **2S-P3-Sia** alone maintained larger sizes than the co-formulated ones after 1 min and 10 min of sonication. While **2S-P11-NH_2_** formed vesicles in 10 mM NaH_2_PO_4_ (pH 4.5) or 10 mM NaOAc-HOAc buffer (pH 5.5), but the results were not consistently reproducible. Fortunately, the addition of cholesterol to **2S-P11-NH_2_** stabilized the vesicles, as evidenced by images obtained from hydrated **2S-P11-NH_2_**/cholesterol (4:1, *w*/*w*) in either buffer (Figure 1D,E). On the other hand, we were unable to obtain a light microscopic image from the hydration of **2S-P1000** in 20 mM phosphate buffer (pH 7.2), suggesting that **2S-P1000** formed SUVs spontaneously. However, large vesicles were visualized when it was co-formulated with phospholipids (Figure 1F).

**Figure 2:**
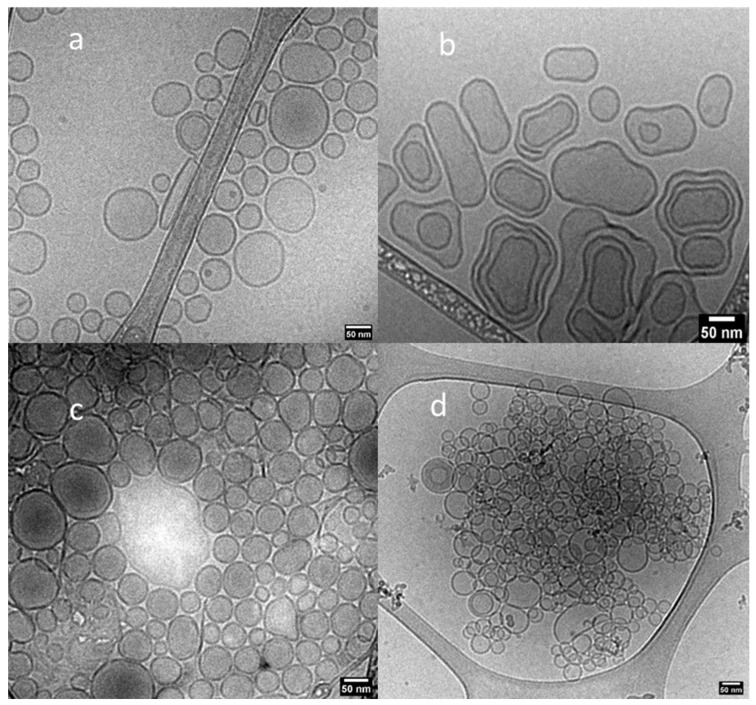
Cryo-TEM images from (**a**) liposomes of DSPC:DSPE:Chol (3:1:1, *w*/*w*) and 1% **2S-P11-F** in 20 mM sodium phosphate buffer (pH 7.2); (**b**) liposomes co-formulated with **2S-P3-Sia** and DSPC:DSPE:Chol (3:1:1, *w*/*w*) in 1:3 (*w*/*w*) and **1% 2S-P11-F** in 20 mM sodium phosphate buffer (pH 7.2); (**c**) liposomes of DPPC/Chol (2:1 *w*/*w*) in 10 mM monobasic phosphate (pH 4.5); and (**d**) liposomes co-formulated with **2S-P11-NH_2_** and DPPC/Chol (2:1 *w*/*w*) in a ratio of 1:4 (*w*/*w*) in 10 mM monobasic phosphate (pH 4.5).

Additionally, we also examined liposomes formulated from phospholipids and **2S-P3-Sia** or **2S-P11-NH_2_** by Cryo-TEM (Figure 2). Both SUVs and MLVs were observed in **2S-P3-Sia**/DSPC:DSPE:Chol (3:1:1 *w*/*w*), which could be related to **2S-P3-Sia** amide lipid. Cryo-TEM also showed that liposomes formulated with **2S-P12-NH_2_** and DPPC/Chol (2:1 *w*/*w*) in 10 mM NaH_2_PO_4_ (pH 4.5) resulted in some degree of aggregation. The addition of those amide lipids to phospholipids provided different surface properties, which should be further investigated and, if possible, exploited for drug delivery. Although their long-term stability is unknown, liposomes containing **2S-P3-Sia**, formed by sonication at 60–70 °C for 45 min, were relatively stable at 4 °C. Although small increases in average size were observed over 4 weeks, all liposomes had maintained sizes smaller than 200 nm. The zeta potentials of those liposomes were essentially the same over the period (Table S1). Since the bacterial targeting and cellular uptake assays were performed under different conditions, we examined the impact of pH on the stability of **2S-P11-NH_2_**-containing liposomes, particularly as their surface charges are pH-dependent. DLS analysis indicated that aggregation occurred in those liposomes at pH 7.0 when co-formulated with phospholipids, which is consistent with the findings from Cryo-TEM. But the short-term stability of those liposomes (Table 2) allowed us to study their interaction with bacteria and phagocytic cells.

### 2.3. Ligand-Directed Binding of Liposomes to Bacteria

**Figure 3:**
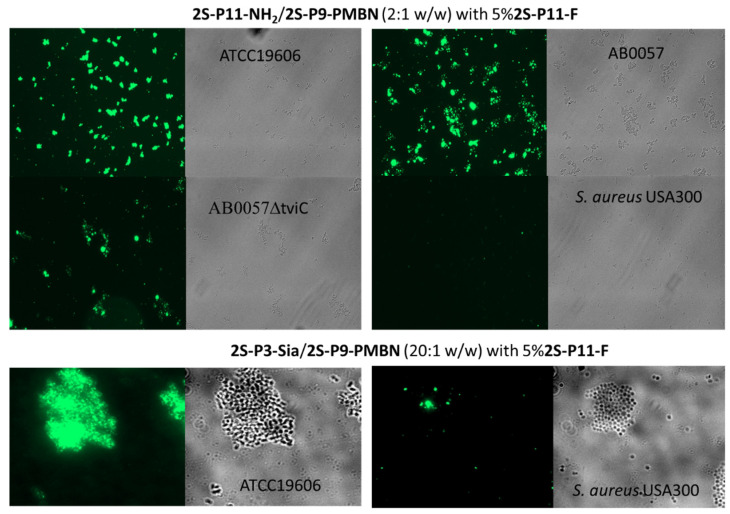
Liposomes incorporated with PMBN target Gram-negative *A. baumannii*. Two images were taken from the same microscopic slide, one with and one without fluorescence. Top: **2S-P11-NH_2_**/**2S-P9-PMBN** (2:1)/5% **2S-P11-F** effectively bound to *A. baumannii* ATCC19606, AB0057, and a mutant AB0057DtviC; Bottom: **2S-P3-Sia**/**2S-P9-PMBN** (20:1)/5% **2S-P11-F** also attached to *A. baumannii* ATCC19606; Liposomes of both formulations did not interact with lipid A-lacking Gram-positive *S. aureus* USA300.

Amide lipids, **2S-P3-Sia** and **2S-P11-NH_2_**, were co-formulated with **2S-P9-PMBN** and **2S-P11-F** to form liposomes, which were used to investigate their interaction with *A. baumannii* bacteria. Unlike **2S-P9-PMBN**, the fluorescent lipid, **2S-P11-F,** which is not water soluble and does not form liposomes by itself, was included for imaging liposome localization. *A. baumannii* ATCC19606, AB0057, and its mutant AB0057DtivC [43] were tested. The bacteria and liposomes were mixed in buffer for 60 min and then transferred to a microscope slide. The same slide was imaged twice, with and without exciting the fluorescein in **2S-P11-F**. The liposome binding to bacteria was evidenced by the detection of fluorescence from bacteria. As expected, PMBN-labeled liposomes, with either **2S-P3-Sia** or **2S-P11-NH_2_**, bound to *A. baumannii* (Figure 3) but not to Gram-positive *S. aureus,* as it lacks lipid A. Although AB0057DtivC was expected to have more accessible lipid A due to its deficient capsule production since its UDP-GalNAcA biosynthesis gene was deleted, no increase in liposome binding was observed. This lack of recognition is suspected to be due to modification of the lipid A and/or lipooligosaccharide (LOS) structures [43]. Future studies using varying PMBN concentrations or other capsule mutants will be necessary to clarify this finding.

**Figure 4:**
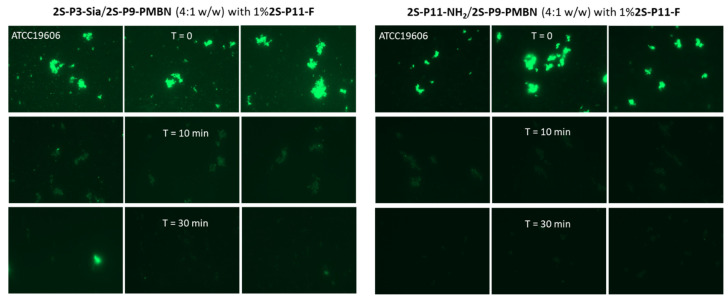
The adsorption of serum proteins disrupted the targeted delivery of liposomes. Liposomes were incubated with 50% mouse serum for 0, 10, and 30 min prior to interaction with *A. baumannii* ATCC19606. Right: liposomes formulated with 4:1 **2S-P11-NH_2_**/**2S-P9-PMBN** with 1% **2S-P11-F**; Left: liposomes formulated with 4:1 **2S-P3-Sia**/**2S-P9-PMBN** with 1% **2S-P11-F**.

We further examined the effect of serum proteins on the liposome–bacteria interaction, particularly if corona formation would disrupt the interaction between PMBN and lipid A. Thus, the liposomes were incubated with an equal volume of naïve mouse serum prior to mixing with bacteria in PBS. Otherwise, the same procedures were followed for bacterial imaging. After 10 min incubation with serum, the bacterial binding capacity of liposomes from **2S-P3-Sia** and **2S-P11-NH_2_** was dramatically decreased, as evidenced by a significant decrease in fluorescent intensity. After 30 min incubation, the liposome–bacteria binding was mostly disrupted (Figure 4). Apparently, the corona formation changed the surface properties as PMBN was likely blocked due to its interaction with serum albumin [44].

Although targeted delivery of liposomes may provide a better therapeutic index, specific ligands such as PMBN, described above, could adsorb serum proteins, including opsonin, which not only disrupt binding to the intended target but may also trigger antibody-mediated clearance. The bacteria-targeted delivery is further complicated by the following facts: first, bacteria may form biofilms as a barrier; second, the bactericidal process by antimicrobial agents mainly involves diffusion into bacterial cells; and third, the bacterial surface lacks common receptors. Thus, it could be more practical that the focus of AMR nanomedicine should be on infection site-directed delivery, improving serum stability and active extravasation, rather than directly targeting bacteria [45].

### 2.4. Liposome Uptake by THP-1 Cells

The phagocytic uptake of liposomes by immune cells results in their rapid clearance from circulation [46]. THP-1 is a human leukemia monocytic cell line that has been extensively used to study monocyte/macrophage functions and has become a common model for estimating the modulation of monocyte and macrophage activities [47]. The contributing factors of cellular uptake include liposome size and surface properties [18,48]. It has been shown that opsonin interacts more favorably with positively charged nanoparticles, and therefore, neutral and negatively charged nanoparticles are observed to have longer circulation times [20,21].

**Figure 5:**
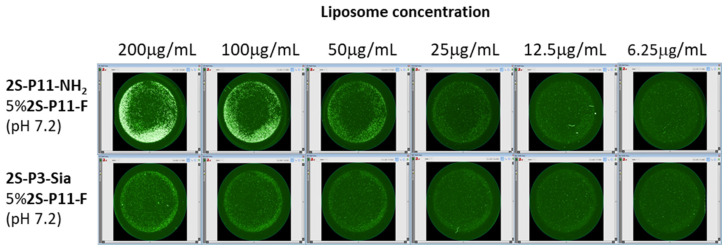
The phagocytic uptake of liposomes by THP-1 cells demonstrates that formulations with negatively charged **2S-P3-Sia** offer more resistance than those containing **2S-P11-NH_2_**. The relative fluorescent intensity observed in cells treated with **2S-P11-NH_2_** containing liposomes at 50–200 μg/mL is 4–5 times stronger than those treated with **2S-P3-Sia** containing liposomes (Table S2).

**Figure 6:**
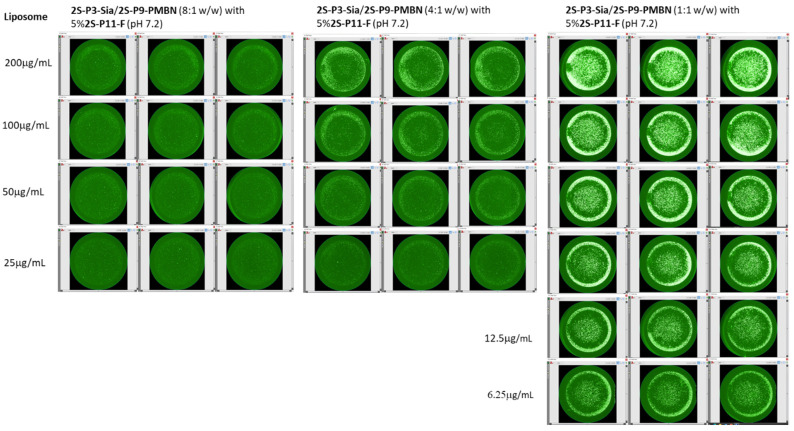
THP-1 cells were used to evaluate in vitro the cellular uptake, and the liposomes were labeled with fluorescent **2S-P11-F**. The cellular uptake was apparently influenced by PMBN, which increased with **2S-P9-PMBN** in the formulation.

We began the study of phagocytic uptake by THP-1 cells using liposomes without serum treatment. The uptake of liposomes containing **2S-P11-NH_2_** was more significant than that of those containing **2S-P3-Sia**, as shown in Figure 5. This could be attributed to the negative charge carried by the sialic acid, as well as possibly due to partial aggregation of the **2S-P11-NH_2_** formulation (Table 2 and Figure 2). We further evaluated the impact of cationic PMBN on liposome uptake by THP-1 cells through varying ratios of **2S-P3-Sia**/**2S-P9-PMBN**, 8:1, 4:1, and 1:1 (*w*/*w*). The results clearly show that the presence of PMBN significantly promotes liposome uptake by THP-1 cells (Figure 6). The enhanced cellular uptake was likely due to the increasing cationic charges of the co-formulated liposomes from PMBN. The fact that PMBN not only interacts with serum proteins but also influences phagocytosis by THP-1 cells further demonstrates the delicate balance in liposome formulations required to avoid unintended consequences.

**Figure 7:**
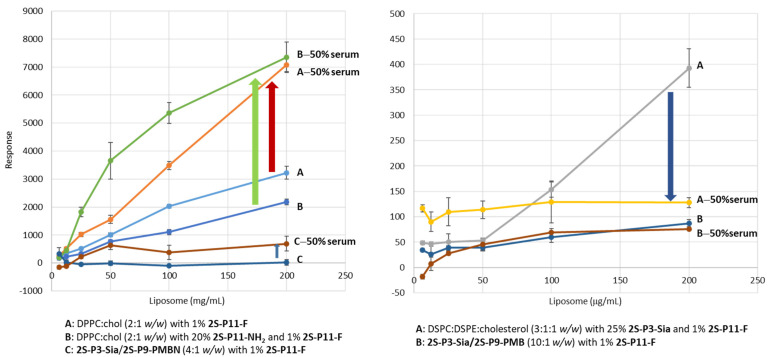
Liposome formulation affects cellular uptake by THP-1 cells and may also influence serum protein adsorption. **Left**: phospholipid liposomes with or without **2S-P11-NH_2_** (A and B) showed significant cellular uptake, while **2S-P3-Sia** can tamp down the phagocytosis (C). Serum treatment dramatically increased cellular uptake of phospholipid/**2S-P11-NH_2_** liposomes (A—50% serum and B—50% serum); **Right**: phospholipid/**2S-P3-Sia** liposomes might be protected from phagocytic uptake by adsorbing certain serum proteins but not opsonin (A vs. A—50% serum), and the sialic acid may play a key role.

Since current nanomedicines are primarily based on phospholipids, the fact that liposomes formulated with **2S-P3-Sia** (see Figure 6) were more resistant to cellular uptake by THP-1 cells encouraged us to explore if the liposomes of amide lipids and phospholipids would provide any beneficial effects such as improving phagocytic resistance, particularly after treatment with naïve mouse sera. As shown in Figure 7 (left), phospholipid (DPPC:cholesterol 2:1 *w*/*w*) liposomes, regardless of the addition of **2S-P11-NH_2_**, had higher cellular uptake than that of **2S-P3-Sia/2S-P9-PMBN** (4:1, *w*/*w*). The uptake was further significantly enhanced after serum treatment, whereas only a slight increase occurred with sialic acid-modified liposomes. In another experiment, shown in Figure 7 (right), we observed a decrease in the cellular uptake of liposomes co-formulated with 25% **2S-P3-Sia** and 75% DSPC:DSPE:cholesterol (3:1:1 *w*/*w*) by THP-1 cells after serum treatment, which may be less significant since their cellular uptake was already relatively low. The results illustrate again that the sialic acid on the liposome surface played a significant role in phagocytic resistance. The function of sialic acid could be similar to PEGylation, namely, regulating protein adsorption.

### 2.5. Liposome Biodistribution and Retention in Mice

To validate whether sialic acid modification can serve as an alternative to PEGylation in enhancing phagocytic resistance, we compared phospholipid-based liposomes incorporating **2S-P3-Sia** and **2S-P1000** in terms of their biodistribution and retention in mice. The study was conducted in two separate experiments due to the constraints on animal handling. The first experiment included three liposome samples, namely, (1) phospholipids:**2S-P1000** (1:1 *w*/*w*); (2) phospholipids:**2S-P3-Sia** (1:1 *w*/*w*); and (3) phospholipids as a reference, which all contained 2.5% **2S-P3-CF770,** a dye-conjugated amide lipid (see Scheme 3) for imaging. The second experiment also involved three liposome formulations, a 3:1 (*w*/*w*) ratio of phospholipids to **2S-P1000** or **2S-P3-Sia** partnered with a phospholipid reference sample. The formulated liposomes were characterized by dynamic light scattering (DLS) prior to the in vivo experiment (Table 3). We observed that the liposome sizes increased slightly compared to the initial measurements (Table S1), possibly due to storage at 4 °C for one month and discrepancies in measurements between two different instruments. The concentrations of the samples were adjusted to have similar fluorescence intensity, which is crucial for obtaining comparable fluorescence signals when performing live animal imaging. Nevertheless, the tissue distribution of the liposomes was examined by the method previously reported [49,50].

**Figure 8:**
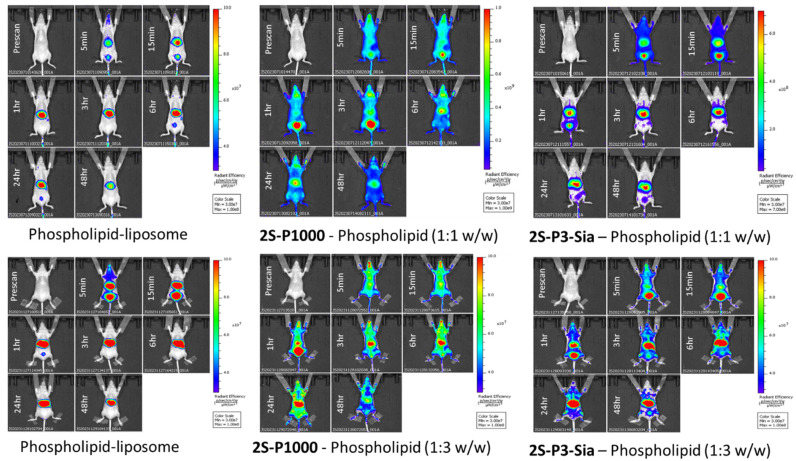
Liposome biodistribution and retention improved by incorporation of amide lipids, **2S-P1000** and **2S-P3-Sia** at 25% and 50% (*w*/*w*). The phospholipids in the formulation are DSPC:DSPE:Chol (3:1:1 *w*/*w*) and 2.5% **2S-P3-CF770**.

The scanned images of mice after administration of liposomes are shown in Figure 8. The phospholipid liposomes quickly accumulated in the liver within a few minutes, as expected. The other formulations incorporating **2S-P1000** and **2S-P3-Sia** demonstrated higher full body signals over time. Formulations, including **2S-P1000**, exhibited the most pronounced distribution and retention in mice. Liposomes containing **2S-P3-Sia** had significantly increased body residence time when compared to phospholipids. In all cases, liposomes accumulated in the liver, although at varying levels, after 48 h. The analysis of internal organ distribution also revealed the presence of liposomes in the spleen, kidney, and lung (see Figures S3 and S5). This experiment provided proof of concept that sialic acid modification can be an alternative to PEGylation. However, optimization in both amide lipids, e.g., using oligosialic acid or other carbohydrates, and formulation are required to achieve the desired outcomes.

## 3. Experimental and Methods

### 3.1. Synthesis of Amide Lipids

Reagents and methods: All pegylated reagents were purchased from BroadPharm (San Diego, CA, USA). Sia-PEG3-azide was purchased from Sussex Research Laboratories (Ottawa, ON, Canada). All reagents purchased from Millipore Sigma (Oakville, ON, Canada) and Thermo Fisher Scientific (Burlington, ON, Canada) were used without further purification. RP-HPLC was conducted using an Agilent 1260 Infinity system equipped with an Agilent 1260 Infinity Multiple Wavelength Detector (Thermo Fisher Scientific, Waltham, MA, USA). Semi-preparative HPLC was performed on a Phenomenex Luna 10 mM C18(2) 100 Å column (250 × 10 mm) using a gradient of water and acetonitrile containing 0.1% trifluoroacetic acid at a flow rate of 4 mL/min. NMR spectra were recorded on Varian Unity INOVA 500 MHz and JEOL 400 MHz (JNM-ECZL400S) spectrometers. ^1^H and ^13^C chemical shifts are reported in δ (ppm) and referenced to residual solvent peaks. NMR experiments in CDCl_3_ or a mixture of CDCl_3_ and CD_3_OD were referenced to 7.26 ppm for ^1^H and 77.16 ppm for ^13^C. ^1^H NMR experiments in D_2_O were referenced to 4.79 ppm. High-resolution mass spectrometry was obtained using a Thermo Fisher Scientific Orbitrap XL and Exploris 240 (Thermo Fisher Scientific, Waltham, MA, USA). Resolution was set to 120,000 or above. An internal mass reference was provided by Ultramark 1621 or fluorene, depending on the mass range. MALDI spectra were collected on a 4800 TOF-TOF (SCIEX, Framingham, MA, USA) in Reflectron mode. α-Cyano-4-hydroxy-cinnamic acid (CHCA) and 2,5-dihydroxybenzoic acid (DHB) were used as the matrix; the matrices were “Mass Spec” grade and used without any further purification.

#### 3.1.1. Stearic Acid N-Hydroxysuccinimide Ester (**1**)

Stearic acid (0.70 g, 2.5 mmol), NHS (0.31 g, 2.7 mmol), and DCC (0.56 g, 2.7 mmol) were dissolved in ethyl acetate (40 mL). The mixture was stirred at room temperature overnight, and the precipitate was filtered. The filtrate was concentrated to a solid, which was recrystallized in anhydrous ethanol to afford **1** as white crystal (0.79 g, 83%). ^1^H NMR (500 MHz, CDCl_3_) δ: 2.83 (br. s, 4H), 2.60 (t, *J* = 7.7 Hz, 2H), 1.74 (q, *J* = 7.5 Hz, 2H), 1.44–1.35 (m, 2H), 1.34–1.21 (m, 26H), 0.88 (t, *J* = 6.7 Hz, 3H); ^13^C NMR (125 MHz, CDCl_3_) δ: 169.3, 168.9, 32.1, 31.1, 29.84, 29.81, 29.77, 29.7, 29.5, 29.2, 28.9, 25.7, 24.7, 22.8, 14.3. HRMS (ESI) m/z: [M + H]^+^ Calcd for C_22_H_40_NO_4_ 382.29519; found 382.29513.

#### 3.1.2. α,ε-*N*,*N*′-Di-stearoyl Lysine (**2**)

To a solution of L-lysine (0.30 g, 2.1 mmol) in DMF-pyridine-TFA at 2:1:0.1 (10 mL) was added **1** (1.5 g, 3.9 mmol), and the mixture was stirred at 70 °C overnight. Ice water was added to precipitate the product, which, after filtration, was suspended in ethyl acetate and vigorously stirred to dissolve unreacted fatty ester. The recovered solid was recrystallized from anhydrous ethanol to give **2** as a white amorphous solid (0.75 g, 53%). ^1^H NMR (500 MHz, 4:3 CDCl_3_:CD_3_OD) δ: 4.14 (dd, *J* = 4.7, 8.4 Hz, 1H), 2.89 (t, *J* = 6.9 Hz, 2H), 1.96 (t, *J* = 7.7 Hz, 2H), 1.88 (t, *J* = 7.5 Hz, 2H), 1.62–1.53 (m, 1H), 1.48–1.38 (m, 1H), 1.37–1.28 (m, 4H), 1.27–1.18 (m, 2H), 1.15–1.07 (m, 2H), 1.06–0.90 (m, 56H), 0.60 (t, *J* = 6.2 Hz, 6H); ^13^C NMR (125 MHz, 4:3 CDCl_3_:CD_3_OD) δ: 174.7, 174.5, 174.1, 51.7, 38.5, 36.0, 35.7, 31.5, 31.0, 29.3, 29.2, 29.01, 28.97, 28.9, 28.4, 25.6, 25.4, 22.5, 22.3, 13.4. HRMS (ESI) m/z: [M + H]^+^ Calcd for C_42_H_83_N_2_O_4_ 679.63474; found 679.63427.

#### 3.1.3. α,ε-*N*,*N*′-Di-stearoyl Lysine N-Hydroxysuccinimide Ester (**3**)

*N*,*N*′-diacyl lysine **2** (0.64 g, 0.94 mmol) was dissolved in chloroform (15 mL) at 50–60 °C. To the solution, NHS (0.30 g, 2.6 mmol) and DCC (0.44 g, 2.2 mmol) were added, and the solution was stirred at 50–60 °C for 3 h, then at room temperature overnight. The products were filtered, and the filtrate was washed with brine (2 × 5 mL). The resulting solid was dried over sodium sulfate, filtered, and the solvent was removed to afford a white residue. Recrystallization twice with anhydrous ethanol gave pure **3** as a white solid (0.50 g, 68%). ^1^H NMR (500 MHz, CDCl_3_, 35 °C) δ: 6.30 (d, *J* = 7.6 Hz, 1H), 5.82 (br. s, 1H), 4.99–4.88 (m, 1H), 3.32–3.20 (m, 2H), 2.83 (br. s, 4H), 2.28–2.21 (m, 2H), 2.16 (t, *J* = 7.5 Hz, 2H), 2.02–1.87 (m, 2H), 1.68–1.43 (m, 8H), 1.34–1.18 (m, 56H), 0.87 (t, *J* = 6.5 Hz, 6H); ^13^C NMR (125 MHz, CDCl_3_, 35 °C) δ: 173.8, 173.3, 168.7, 168.3, 50.3, 38.4, 36.9, 36.4, 32.1, 31.7, 29.8, 29.69, 29.66, 29.54, 29.49, 29.4, 29.0, 26.0, 25.74, 25.66, 22.8, 21.8, 14.2. HRMS (ESI) m/z: [M + H]^+^ Calcd for C_46_H_86_N_3_O_6_ 776.65111; found 776.65048.

#### 3.1.4. α,ε-*N*,*N*′-Di-stearoyl Lysine (α-Sialo-PEG3-amine)-amide (**4**, **2S-P3-Sia**)

To a solution of Sia-PEG3-amine (33.6 mg, 0.076 mmol) in a mixture of DMF and chloroform (2:1 mL) was added ester **3** (40.9 mg, 0.053 mmol) in chloroform (1.0 mL). The pH of the solution was adjusted to 7 with 10% triethylamine in chloroform. The solution was stirred overnight at room temperature. The reaction mixture was diluted with chloroform (5 mL) and extracted with water (5 × 5 mL). The aqueous solutions were combined and lyophilized to yield **4** as a white solid (43.8 mg, 75%). ^1^H NMR (400 MHz, 2:1 CDCl_3_:CD_3_OD) δ: 4.08–4.02 (m, 1H), 3.74–3.19 (m, 18H), 3.16–3.08 (m, 1H), 2.92 (t, *J* = 6.9 Hz, 2H), 2.57 (dd, *J* = 4.1, 12.6 Hz, 1H), 2.00 (t, *J* = 7.6 Hz, 2H), 1.93 (t, *J* = 7.6 Hz, 2H), 1.81 (s, 3H), 1.56–1.32 (m, 7H), 1.31–1.22 (m, 2H), 1.14–0.97 (m, 58H), 0.65 (t, *J* = 6.0 Hz, 6H); ^13^C NMR (100 MHz, 2:1 CDCl_3_:CD_3_OD) δ: 174.7, 174.6, 174.2, 173.6, 172.5, 100.4, 73.1, 70.9, 70.6, 69.7, 69.43, 69.38, 68.8, 67.4, 63.3, 62.9, 53.0, 52.6, 40.6, 38.8, 38.6, 36.2, 35.8, 31.7, 31.3, 29.42, 29.38, 29.33, 29.30, 29.19, 29.16, 29.11, 29.08, 28.5, 25.7, 25.5, 22.6, 22.4, 22.0, 13.6. HRMS (ESI) m/z: [M + H]^+^ Calcd for C_59_H_113_N_4_O_14_ 1101.82478; found 1101.82483.

#### 3.1.5. α,ε-*N*,*N*′-Di-stearoyl Lysine (PEG1000-Amine)-amide (**5**, **2S-P1000**)

PEG1000-amine (72 mg, 0.067 mmol) in chloroform (2 mL) was added to a solution of **3** (40 mg, 0.051 mmol) in chloroform (2 mL). The pH was adjusted by the addition of 10% triethylamine in chloroform. The mixture was stirred at room temperature overnight, diluted with chloroform (4 mL) and washed with water (8 × 1 mL). The organic layer was concentrated, water added (4 mL) and briefly sonicated. The aqueous solution was frozen and lyophilized to give **5** as a white amorphous solid (81 mg, 91%). ^1^H NMR (400 MHz, CDCl_3_) δ: 7.00 (br. s, 1H), 6.42 (d, *J* = 7.8 Hz, 1H), 5.88 (br. s, 1H), 4.43–4.36 (m, 1H), 3.74–3.69 (m, 2H), 3.68–3.58 (m, 90H), 3.57–3.52 (m, 2H), 3.48–3.40 (m, 2H), 3.26–3.18 (m, 2H), 3.44–3.38 (m, 2H), 2.20 (t, *J* = 7.3 Hz, 2H), 2.14 (t, *J* = 7.8 Hz, 2H), 1.86–1.75 (m, 1H), 1.71–1.46 (m, 7H), 1.38–1.19 (m, 58H), 0.87 (t, *J* = 7.1 Hz, 6H); ^13^C NMR (100 MHz, CDCl_3_) δ: 173.6, 173.4, 172.0, 72.8, 70.74, 70.70, 70.4, 70.3, 69.8, 61.8, 52.7, 39.4, 38.9, 36.9, 36.8, 32.6, 32.1, 29.85, 29.80, 29.7, 29.6, 29.5, 29.0, 26.0, 25.9, 22.8, 22.5, 14.3. HRMS (ESI) m/z: [M + H]^+^ Calcd for C_90_H_180_N_3_O_27_ 1735.27987; found 1735.27963.

#### 3.1.6. α,ε-*N*,*N*′-Di-stearoyl Lysine (Boc-*N*-PEG11-amine)-amide (**6**, **2S-P11-NHBoc**)

To a solution of *t*-Boc-*N*-amido-PEG11-amine (73 mg, 0.11 mmol) in chloroform (2 mL) was added ester **3** (64 mg, 0.082 mmol) in chloroform (2 mL). The pH was adjusted by the addition of 10% triethylamine in chloroform. The mixture was stirred at room temperature overnight, diluted with chloroform (5 mL) and washed with water (5 × 5 mL). The organic layer was dried over sodium sulfate, filtered and concentrated to give product **6** (95 mg, 88%). The product was used without further purification. ^1^H NMR (500 MHz, CDCl_3_) δ: 6.87 (br. s, 1H), 6.50 (d, *J* = 7.5 Hz, 1H), 5.97 (br. s, 1H), 5.06 (br. s, 1H), 4.40–4.31 (m, 1H), 3.67–3.55 (m, 40H), 3.54–3.47 (m, 4H), 3.43–3.35 (m, 2H), 3.30–3.24 (m, 2H), 3.22–3.14 (m, 2H), 2.17 (t, *J* = 7.5 Hz, 2H), 2.11 (t, *J* = 7.6 Hz, 2H), 1.82–1.73 (m, 1H), 1.69–1.45 (m, 7H), 1.40 (s, 9H), 1.35–1.16 (m, 58H), 0.84 (t, *J* = 6.7 Hz, 6H); ^13^C NMR (125 MHz, CDCl_3_) δ 173.6, 173.4, 171.9, 156.1, 79.2, 70.6, 70.5, 70.3, 69.7, 52.7, 40.4, 39.3, 38.8, 36.8, 36.6, 32.3, 32.0, 29.8, 29.7, 29.6, 29.49, 29.46, 29.4, 29.0, 28.5, 25.9, 25.8, 22.7, 22.4, 14.2. HRMS (ESI) m/z: [M + H]^+^ Calcd for C_71_H_141_N_4_O_16_ 1306.03371; found 1306.03303.

#### 3.1.7. α,ε-*N*,*N*′-Di-stearoyl Lysine (Boc-*N*-PEG3-amine)-amide (**7**, **2S-P3-NHBoc**)

Ester **3** (85 mg, 0.11 mmol) dissolved in chloroform (1 mL) was added to a solution of *t*-Boc-*N*-amido-PEG3-amine (42 mg, 0.14 mmol) in chloroform (2 mL). The pH was adjusted by the addition of 10% triethylamine in chloroform. The mixture was stirred at room temperature overnight, diluted with chloroform (5 mL) and washed with water (5 × 5 mL). The organic layer was dried over sodium sulfate, filtered and concentrated to give product **7** (92 mg, 88%). The product was used without further purification. ^1^H NMR (400 MHz, CDCl_3_) δ: 6.83 (br. s, 1H), 6.39 (d, *J* = 5.3 Hz, 1H), 5.83 (br. s, 1H), 5.22 (br. s, 1H), 4.45–4.35 (m, 1H), 3.66–3.605 (m, 8H), 3.58–3.52 (m, 4H), 3.48–3.41 (m, 2H), 3.34–3.26 (m, 2H), 3.25–3.16 (m, 2H), 2.20 (t, *J* = 7.3 Hz, 2H), 2.14 (t, *J* = 7.3 Hz, 2H), 1.85–1.74 (m, 1H), 1.71–1.47 (m, 7H), 1.44 (s, 9H), 1.38–1.18 (m, 58H), 0.87 (t, *J* = 6.4 Hz, 6H); ^13^C NMR (100 MHz, CDCl_3_) δ: 173.6, 173.5, 172.0, 156.3, 79.3, 70.7, 70.6, 70.33, 70.26, 69.8, 52.7, 40.4, 39.4, 38.8, 37.0, 36.8, 32.4, 32.1, 29.85, 29.82, 29.80, 29.7, 29.6, 29.5, 29.1, 29.4, 28.6, 26.0, 25.8, 22.8, 22.5, 14.3. HRMS (ESI) m/z: [M + H]^+^ Calcd for C_55_H_109_N_4_O_8_ 953.82399; found 953.82046.

#### 3.1.8. α,ε-*N*,*N*′-Di-stearoyl Lysine (PEG11-Amine)-amide (**8**, **2S-P11-NH_2_**)

Compound **6** (35 mg, 0.027 mmol) was dissolved in 3:1:1 CHCl_3_-H_2_O-TFA (10 mL). The solution was refluxed for 6 h, diluted with chloroform (20 mL) and washed with water (5 × 5 mL) and saturated aqueous sodium bicarbonate solution (2 × 5 mL). The organic layer was dried over sodium sulfate, filtered, and concentrated. The residue obtained was sonicated after the addition of water (5 mL), and the aqueous solution was frozen and lyophilized to yield a white solid **8** (26 mg, 81%). ^1^H NMR (500 MHz, CDCl_3_) δ: 7.34 (br. s, 1H), 7.15 (d, *J* = 7.8 Hz, 1H), 6.28 (br. s, 1H), 4.39–4.33 (m, 1H), 3.81 (t, *J* = 4.7 Hz, 2H), 3.74–3.68 (m, 2H), 3.67–3.57 (m, 38H), 3.54 (t, *J* = 5.5 Hz, 2H), 3.44–3.38 (m, 2H), 3.27–3.13 (m, 4H), 2.21 (t, *J* = 7.8 Hz, 2H), 2.15 (t, *J* = 7.0 Hz, 2H), 1.82–1.73 (m, 1H), 1.72–1.64 (m, 1H), 1.63–1.44 (m, 6H), 1.42–1.32 (m, 2H), 1.31–1.17 (m, 56H), 0.86 (t, *J* = 7.8 Hz, 6H); ^13^C NMR (125 MHz, CDCl_3_) δ: 173.9, 173.8, 172.8, 70.54, 70.51, 70.44, 70.37, 70.32, 70.29, 70.2, 70.1, 70.0, 69.8, 67.2, 53.3, 40.2, 39.3, 38.6, 36.8, 36.6, 32.04, 32.01, 29.83, 29.78, 29.7, 29.55, 29.51, 29.48, 28.8, 26.0, 25.9, 22.8, 22.5, 14.2. HRMS (ESI) m/z: [M + H]^+^ Calcd for C_66_H_133_N_4_O_14_ 1205.98128; found 1205.98189.

#### 3.1.9. α,ε-*N*,*N*′-Di-stearoyl Lysine (PEG3-Amine)-amide (**9**, **2S-P3-NH_2_**)

Compound **7** (42 mg, 0.044 mmol) was dissolved in 3:1:1 CHCl_3_-H_2_O-TFA (10 mL). The solution was refluxed for 6 h and concentrated. The crude product was dissolved in chloroform (5 mL) and washed with water (5 × 5 mL) and a saturated aqueous solution of sodium bicarbonate (2 × 5 mL). The organic layer was dried over sodium sulfate, filtered, and concentrated. The product was sonicated after addition of water (5 mL), the solution was frozen and lyophilized to yield a white solid **9** (28 mg, 75%). ^1^H NMR (400 MHz, CDCl_3_) δ: 8.51 (br. s, 1H), 6.73 (d, *J* = 7.6 Hz, 1H), 6.22 (br. s, 1H), 4.28–4.20 (m, 1H), 3.91–3.52 (m, 12H), 3.30–3.06 (m, 4H), 2.24–2.11 (m, 4H), 1.82–1.45 (m, 8H), 1.42–1.15 (m, 58H), 0.87 (t, *J* = 6.4 Hz, 6H); ^13^C NMR (100 MHz, CDCl_3_) δ: 174.7, 174.0, 172.8, 70.5, 70.1, 69.6, 69.3, 66.7, 53.5, 39.7, 39.5, 38.7, 36.9, 36.4, 32.1, 31.9, 29.9, 29.8, 29.70, 29.68, 29.6, 29.5, 29.4, 28.8, 26.0, 25.7, 22.8, 22.7, 14.3. HRMS (ESI) m/z: [M + H]^+^ Calcd for C_50_H_101_N_4_O_6_ 853.77156; found 853.76723.

#### 3.1.10. α,ε-*N*,*N*′-Di-stearoyl Lysine (Fluorescein-5-EX-*N*-amido-PEG11 amine)-amide (**10**, **2S-P11-F**)

To a solution of **8** (11 mg, 9.1 mmol) in chloroform (2 mL) was added fluorescein-5-EX-NHS ester (5 mg, 8.4 mmol) in DMSO (0.5 mL). The mixture was kept at room temperature overnight, then washed with brine (3 × 5 mL), dried over sodium sulfate, filtered, and the solvent evaporated. After lyophilization, the product **10** (9 mg, 63%) was obtained as an orange solid. ^1^H NMR (400 MHz, 2:1 CDCl_3_:CD_3_OD) δ: 8.04 (s, 0.5H), 7.75 (d, *J* = 8.5 Hz, 0.5H), 6.91 (d, *J* = 8.2 Hz, 0.5H), 6.49–6.42 (m, 2H), 6.34–6.28 (m, 1H), 4.12–4.06 (m, 1H), 3.46–3.36 (m, 29H), 3.34–3.27 (m, 2H), 3.22–3.12 (m, 4H), 2.92 (t, *J* = 6.6 Hz, 2H), 2.72 (t, *J* = 6.9 Hz, 1H), 2.36 (t, *J* = 6.9 Hz, 1H) 1.99 (t, *J* = 7.6 Hz, 2H), 1.92 (t, *J* = 7.3 Hz, 2H), 1.58–1.46 (m, 1H), 1.44–1.32 (m, 5H), 1.31–1.22 (m, 2H), 1.14–0.95 (m, 62H), 0.64 (t, *J* = 6.4 Hz, 6H); ^13^C NMR (100 MHz, 2:1 CDCl_3_:CD_3_OD) δ: 174.6, 174.3, 172.5, 172.1, 169.8, 169.2, 153.1, 139.8, 129.1, 128.8, 126.1, 125.0, 115.6, 113.1, 110.5, 102.5, 70.1, 69.9, 69.8, 69.6, 69.3, 69.2, 52.7, 39.1, 38.9, 38.6, 36.2, 36.0, 35.6, 31.7, 31.6, 29.4, 29.3, 29.10, 29.06, 28.4, 28.0, 25.7, 25.6, 22.5, 22.4, 13.6. HRMS (ESI) m/z: [M + H]^+^ Calcd for C_91_H_150_N_5_O_21_ 1681.05385; found 1681.05323.

#### 3.1.11. α,ε-*N*,*N*′-Di-stearoyl Lysine (CF770-*N*-Amido-PEG3-amine)-amide (**11**, **2S-P3-CF770**)

Amine **9** (0.85 mg, 1 mmol) dissolved in chloroform (0.5 mL) was added to a solution of CF770-NHS (1 mmol) in DMF (0.5 mL). The mixture was kept at room temperature overnight, diluted with chloroform (4 mL) and washed with water (5 × 1 mL). The organic layer was concentrated. To the residue, water (5 mL) was added, and the mixture was briefly sonicated. The aqueous solution was frozen and lyophilized to yield the product as a green amorphous solid **11** (2.6 mg, 65%).

#### 3.1.12. α,ε-*N*,*N*′-Di-stearoyl Lysine (*N*-Maleimido-PEG9 amine)-amide (**12**, **2S-P9-Mal**)

Ester **3** (150 mg, 0.19 mmol) and *N*-maleimido-PEG9-amine TFA salt (200 mg, 0.33 mmol) were dissolved in chloroform (10 mL), and the pH was adjusted to neutral by the addition of 10% triethylamine in chloroform. The mixture was stirred at room temperature for 3 h, diluted by the addition of chloroform (10 mL), washed with brine (2 × 15 mL), dried over sodium sulfate, and the filtrate evaporated to yield a yellowish-white solid **12** (184 mg, 75%). ^1^H NMR (500 MHz, CDCl_3_) δ: 6.84 (br. s, 1H), 6.70 (s, 2H), 6.51 (br. s, 1H), 6.43 (d, *J* = 7.8 Hz, 1H), 5.86 (br. s, 1H), 4.43–4.36 (m, 1H), 3.84 (t, *J* = 7.3 Hz, 2H), 3.68–3.58 (m, 28H), 3.57–3.51 (m, 4H), 3.46–3.38 (m, 4H), 3.26–3.18 (m, 2H), 2.51 (t, *J* = 7.3 Hz, 2H), 2.21 (t, *J* = 7.3 Hz, 2H), 2.15 (t, *J* = 7.8 Hz, 2H), 1.81–1.75 (m, 1H), 1.71–1.44 (m, 7H), 1.38–1.16 (m, 58H), 0.88 (t, *J* = 6.3 Hz, 6H); ^13^C NMR (125 MHz, CDCl_3_) δ 173.7, 173.5, 172.0, 170.7, 169.9, 134.4, 70.7, 70.4, 69.9, 69.8, 52.7, 39.4, 38.9, 37.0, 36.8, 34.7, 34.5, 32.5, 32.1, 29.9, 29.7, 29.6, 29.5, 29.0, 26.0, 25.9, 22.8, 22.5, 14.3. HRMS (ESI) m/z: [M + H]^+^ Calcd for C_69_H_130_N_5_O_15_ 1268.95579; found 1268.95634.

#### 3.1.13. Tetra-N-Boc-PMBN-SAc (**13**)

To a solution of tetra-*N*-Boc-PMBN (90.4 mg, 0.066 mmol) in DMSO (1.4 mL) was added *N*-succinimidyl *S*-acetylthioacetate (SATA, 32.1 mg, 0.14 mmol) in DMSO (100 mL), which was followed by the addition of 0.2 M dibasic sodium phosphate (0.3 mL). The homogeneous solution was left at room temperature for 1 h. Water (4 mL) was added, and the resultant mixture was centrifuged (3000 rpm, 20 min). The pellet was washed with water (4 × 3 mL). The pellet was evenly suspended in water (5 mL) and lyophilized to yield a white solid **13** (66 mg, 0.045 mmol, 67%). ^1^H NMR (500 MHz, 1:1 CDCl_3_:CD_3_OD) δ: 7.00–6.87 (m, 5H), 6.17 (br. s, 1H), 5.98 (br. s, 1H), 5.89–5.78 (m, 2H), 4.12–3.70 (m, 10H), 3.39 (s, 2H), 3.28–3.15 (m, 1H), 2.99–2.55 (m, 10H), 2.10 (s, 3H), 1.90–1.22 (m, 13H), 1.19–1.07 (m, 36H), 1.10–0.84 (m, 6H), 0.60–0.35 (m, 7H). HRMS (ESI) m/z: [M + H]^+^ Calcd for C_67_H_111_N_14_O_21_S 1479.77634; found 1479.77393.

#### 3.1.14. PMBN-SAc (**14**)

Compound **13** (147.3 mg, 0.1 mmol) was dissolved in a 3:1:1 DCM-H_2_O-TFA mixture (10 mL) and refluxed overnight. The solution was concentrated, and the crude material was applied to a C18 column eluting with an increasing gradient of acetonitrile in water containing 0.1% TFA. The fractions were collected and lyophilized to a white solid **14** (76.5 mg, 71%). ^1^H NMR (500 MHz, D_2_O) δ: 7.42–7.33 (m, 3H), 7.32–7.25 (m, 2H), 4.64–4.45 (m, 3H), 4.38–4.17 (m, 7H), 3.80 (s, 2H), 3.43–3.33 (m, 1H), 3.20–3.01 (m, 8H), 2.96–2.77 (m, 2H), 2.44 (s, 3H), 2.35–1.79 (m, 11H), 1.55–1.38 (m, 2H), 1.24 (d, *J* = 6.3 Hz, 3H), 1.21 (d, *J* = 6.3 Hz, 3H), 0.86–0.66 (m, 7H). HRMS (ESI) m/z: [M + H]^+^ Calcd for C_47_H_79_N_14_O_13_S 1079.56663; found 1079.56778.

#### 3.1.15. PMBN-SH (**15**)

Hydroxylamine solution (0.8 mL) prepared from hydroxylamine hydrochloride (18 mg) in 0.2 M dibasic sodium phosphate (2.5 mL) was added to **14** (38.3 mg, 35 mmol) and left at room temperature for 1.5 h. The crude material was purified on a C18 column eluting with an increasing gradient of acetonitrile in water containing 0.1% TFA. Pure fractions for both the sulfhydryl and disulfide were collected and lyophilized to yield a white solid **15** (14.4 mg, 39%) and the disulfide dimer (16.8 mg, 46%). The disulfide was treated with tris(2-carboxyethyl)phosphine hydrochloride (TCEP) and purified again on the C18 column to recover **15**. ^1^H NMR (500 MHz, D_2_O) δ: 7.45–7.33 (m, 3H), 7.31–7.24 (m, 2H), 4.59 (t, *J* = 8.3 Hz, 1H), 4.55–4.48 (m, 2H), 4.40–4.18 (m, 7H), 3.70 (d, *J* = 14.1 Hz, 1H), 3.60 (d, *J* = 14.1 Hz, 1H), 3.40–3.30 (m, 1H), 3.20–3.01 (m, 8H), 2.93–2.75 (m, 2H), 2.33–1.80 (m, 11H), 1.54–1.29 (m, 2H), 1.26 (d, *J* = 6.4 Hz, 3H), 1.20 (d, *J* = 6.0 Hz, 3H), 0.91–0,66 (m, 7H). HRMS (ESI) observed as a disulfide, m/z: [2M + 2H]^2+^ Calcd for C_90_H_152_N_28_O_24_S_2_ 1036.54824; found 1036.54897.

#### 3.1.16. α,ε-*N*,*N*′-Di-stearoyl Lysine (PMBN-*N*-Maleimido-PEG9 amine)-amide (**16**, **2S-P9-PMBN**)

A solution of maleimide **12** (11.7 mg, 9.2 mmol) and thiol **15** (14.4 mg, 13.8 mmol) in chloroform-methanol (2:1 mL) was adjusted to pH 6–7 by the addition of 10% triethylamine in methanol. The mixture was kept at room temperature overnight. After removal of the solvent, the residue was dissolved in chloroform and water (5 mL each). The water phase was extracted with chloroform (4 × 5 mL) and then frozen and lyophilized to a white amorphous solid **16** (15.7 mg, 74%). ^1^H NMR (400 MHz, 2:1 CDCl_3_:CD_3_OD) δ: 7.01–6.86 (m, 5H), 4.17–3.66 (m, 9H), 3.49–3.41 (m, 1H), 3.39–3.27 (m, 31H), 3.25–3.18 (m, 4H), 3.10–3.04 (m, 2H), 2.89–2.81 (m, 4H), 2.80–2.54 (m, 7H), 2.31–1.95 (m, 2H), 1.91 (t, *J* = 7.6 Hz, 2H), 1.84 (t, *J* = 7.5 Hz, 2H), 1.80–1.51 (m, 2H), 1.49–1.13 (m, 8H), 1.04–0.98 (m, 12H), 0.97–0.90 (m, 55H), 0.88–0.82 (m, 3H), 0.56 (t, *J* = 6.7 Hz, 6H), 0.48–0.26 (m, 7H); HRMS (ESI) m/z: [M + 2H]^2+^ Calcd for C_114_H_207_N_19_O_27_S 1153.25593; found 1153.25575.

### 3.2. Liposome Formulation and Characterization

Since the amide lipids are neither soluble in ethanol nor in diethyl ether, the thin film method was used to formulate liposomes. Briefly, the lipids were dissolved in chloroform-methanol in a round-bottom flask, and the solvents were evaporated under diminished pressure to create a thin film of lipids. The film was hydrated in buffers with occasional shaking to obtain mostly LUVs, which were transformed to SUVs after bath sonication at 60–70 °C for 45 min. The phospholipids used in formulation are 1,2-distearoyl-sn-glycero-3-phosphocholine (DSPC), 1,2-Distearoyl-sn-glycero-3-phosphoethanolamine (DSPE), and 1,2-dipalmitoyl-sn-glycero-3-phosphocholine (DPPC). The liposomes were easily filtered through microfilter (0.45 mm) and stored in glass vials at 4 °C. The liposome samples were used for physical characterization, bacterial binding, phagocytosis assay and for in vivo biodistribution imaging.

#### 3.2.1. Microscopy Images

5 mL of liposomes (1 mg lipids/mL) was added to a frosted slide with a coverslip at a 45° angle to avoid air bubbles. A drop of immersion oil was added to the center of the coverslip. Images were taken using 100× oil lens on an Olympus BX51 (Microscope Central, Feasterville-Trevose, PA, USA) and captured by a Q Imaging MicroPublisher 5.0 RTV camera (QImaging Corp, Surrey, BC, Canada).

#### 3.2.2. Cryo-TEM Images

A specimen of each formulation was prepared using the plunge freezing method. Briefly, one drop of formulation was placed on lacey carbon film supported TEM grids which were pre-treated with glow discharge; then, excess solution was blotted away from the TEM grid before being rapidly plunged into liquid ethane (−184 °C); the frozen samples on TEM grids were transferred to TEM and imaged at a temperature of −180 °C. All Cryo-TEM images were obtained on a JEOL2200FS TEM with an in-column Omega energy filter (JEOL Ltd., Tokyo, Japan) and running at an accelerating voltage of 200 kV. A 10 eV energy slit and defocusing were applied for contrast enhancement.

#### 3.2.3. Liposome Size

Liposome sizes were measured at room temperature by DLS using a Zetasizer nanoseries Nano-ZS ZEN3600 (Malvern Instruments, Westborough, MA, USA). Samples: 10 mL of liposomes (1 mg lipids/mL) was diluted to 1 mL total volume in a disposable ZEN0040 plastic cuvette. Assumed material refractive index of 1.33, material absorption of 0.010, and viscosity of 0.8872 at 24 °C, after 120 s of equilibration time, the samples were measured three times at 173° backscatter measurement angle, each with 11 runs of 10 s duration.

#### 3.2.4. Measurement Parameters for Zeta Potential (ZP)

The samples were given 120 s equilibration time. Minimum 10 and maximum 100 runs were recorded for each measurement. Three measurements were taken in Malvern disposable folded capillary cells and analyzed using the Smoluchowski model, assuming a value of 1.5 for the function f(Ka).

### 3.3. In Vitro PMBN-Mediated Liposome Binding to Bacteria

The experiment has the following steps: (1) streaking bacterial strains (*A. baumannii* ATCC19606, AB0057, the capsule deficient mutant AB0057ΔtviC and *S. aureus* USA300) from frozen stock onto LB agar plates and incubated overnight at 37 °C; (2) collecting a loopful of each strain and suspending into 3 mL sterile saline; (3) adding bacteria to 100 mL TSB broth in a baffle-bottom flask until OD600 = 0.95–0.105; (4) culturing bacteria at 37 °C, 200 rpm shaking until late exponential phase was reached (OD600 = 1.3–1.5); (5) transferring 1.5 mL to a microcentrifuge tube (1 tube/compound for each strain) and spin down cells at 21,000× *g* for 5 min at room temperature; (6) resuspending cells in PBS with liposome (1 mg lipid/mL); (7) placing tubes on a platform shaker and shaking at medium speed for 60 min in the dark at room temperature; (8) adding 1 mL PBS to each tube and spin down at 21,000× *g* for 5 min at room temperature; (9) washing once with 1 mL PBS and spin down at 21,000× *g* for 5 min at room temperature; (10) resuspending cells in 100 µL distilled water; (11) add 1 µL cell suspension on each microscope slide, and then add 5 µL mounting medium (Agilent, Santa Clara, CA, USA). Apply a coverslip and seal edges with coverslip sealant; and (12) keeping microscope slides overnight at 4 °C to harden prior to microscopic imaging.

### 3.4. The Effect of Serum Treatment of Liposomes on Bacteria-Binding

The experiment procedures are the same as described in the previous section, except only *A. baumannii* ATCC19606 was tested, and 80 µL of serum cultured liposomes (1 mg lipids/mL) from 10 min to 4 h were used in step 6, which were obtained from admix liposomes (2 mg lipids/mL) with an equal volume of sera collected from naïve mice. Additionally, an untreated control was prepared, consisting of 80 µL liposomes (2 mg lipids/mL) admixed with an equal volume of PBS.

### 3.5. Liposome Uptake by THP-1 Cells

THP-1 cells were spun down and resuspended in growth media to 20 mL of 1.5 × 10^5^ cells/mL. Phorbol 12-myristate 13-acetate in DMSO was added to the media at 100 ng/mL, and 100 μL of the cell suspensions was then added to the 96-well plate. No cells were added to the cell-free negative control wells; instead, 100 mL growth media was added. The samples were incubated for 72 h, following which the media was removed, and liposomes were added to growth media to the indicated concentrations. The plate was incubated for 4 h, then washed gently three times in growth media and once with PBS. Wells were then treated with 1 mM Cell Trace Yellow in PBS for 20 min, emptied, and washed once with PBS, then resuspended in Invitrogen Live Cell Imaging Solution containing 5 mM DRAQ nuclear stain and imaged on a Mirrorball.

### 3.6. Biodistribution and Retention in Mice (In Vivo Imaging)

An amide lipid conjugate of CF770 (**2S-P3-CF770**) was incorporated into liposome formulations for in vivo optical imaging studies using an IVIS Lumina III preclinical animal imager (Perkin Elmer, Waltham, MA, USA). Liposome formulations, from phospholipid, **2S-P1000** and **2S-P3-Sia,** were first normalized for fluorescent intensity and injected intravenously in SKH1 Elite hairless mice at 2.5–5 mg lipid/mL. Particle concentrations were measured using ZetaView^®^ Nanoparticle Tracking Analyzer (Particle Metrix Inc., Holly Springs, NC, USA). The movement and patterns of particles emitting scattered light are measured using a highly sensitive CMOS camera. Individual particles in the field of view are counted and tracked in short video clips, creating accurate concentration calculations and particle size distributions. Animals were imaged at pre-scan, 5 min, 15 min, 1 h, 2 h, 3 h, 6 h, 24 h, and 48 h in both dorsal and ventral positions. At the end of the experiment (48 h), animals were perfused with heparinized saline (20 mL, 50 U/mL), and the dissected organs (brain, heart, lung, liver, spleen, and kidney) were imaged ex vivo. Total fluorescence radiance efficiency for organs was determined using Living Image 4.1 software (PerkinElmer, Waltham, MA, USA). SKH1 mice were selected for optical imaging as they lack hair and skin pigmentation, which improves signal-to-noise ratio due to reduced light scattering and absorption. SKH1 elite hairless mice were purchased from Charles Rivers Laboratories, St. Constant, QC, Canada and housed in microisolators at the animal care facility at the National Research Council of Canada (Ottawa, ON, Canada). In addition to experience in using this mouse strain for our other research, their hairlessness allows for easier application and observation of topical and tail vein treatments and UV exposure, while their immunocompetent nature allows for studying immune response. We used isoflurane by inhalation as an anesthetic substance (4% for induction and 1–1.5% for maintenance). At the end of the experiment, the mice were under deep anesthesia with isoflurane, followed by intracardiac perfusion of heparinized saline (20 mL, 50 U/mL).

### 3.7. Animal Care

All studies were conducted in accordance with regulations and guidelines reviewed and approved by the Human Health Therapeutics Animal Care Committee and were conducted in facilities accredited by the Canadian Council on Animal Care, which are in agreement with those outlined in ARRIVE guidelines.

## 4. Conclusions

We have attempted to address some of the current challenges facing nanomedicine and lipid nanoparticle formulations. We outlined a synthetic method to produce amide-based lipids from lysine and stearic acid, featuring various hydrophilic head groups. Most of our amide lipids readily form liposomes separately or when co-formulated with commercial phospholipids and are relatively stable, as indicated by DLS and zeta potential data. As a proof of concept, we demonstrated that lipid A ligand (PMBN)-modified liposomes were able to target Gram-negative bacteria such as *A. baumannii*. However, such targeted delivery was significantly inhibited after serum treatment. Additionally, we found that PMBN-modified liposomes significantly increased their cellular uptake by THP-1 cells. Further studies on the co-formulation of phospholipids and amide lipids showed that sialic acid-modified liposomes provided more phagocytic resistance than liposomes containing **2S-P11-NH_2_** or only commercial phospholipids. Our in vivo investigation also yielded encouraging results, as we observed increased biodistribution and retention times in mice from formulations containing **2S-P3-Sia** and **2S-P1000** when compared to commercial phospholipids alone, suggesting that sialylation of nanoparticles may be an attractive alternative to PEGylation.

Although we have established some benefits of amide lipids in this study, much could be improved by optimizing formulations, including the incorporation of other carbohydrates. Further investigation into payload encapsulation, as well as protein corona characterization, is required to understand the metabolism and safety of the amide lipids.

## Figures and Tables

**Table 1:** Amide lipids and phospholipids form bilayer vesicles observed by light microscopy.

Sample	Lipids	Buffer	Nanoparticle
A	**2S-P3-Sia**	20 mM phosphate (pH 7.2)	Bilayer vesicles
B	**2S-P3-Sia**/Phospholipids (1:1 *w*/*w*)		
C	**2S-P3-Sia**/Phospholipids (1:2 *w*/*w*)		
D	**2S-P11-NH_2_**/Cholesterol (4:1 *w*/*w*)	10 mM NaH_2_PO_4_ (pH 4.5)	Bilayer vesicles
E		10 mM NaOAc-HOAc (pH 5.5)	
F	**2S-P1000**/phospholipids (1:1 *w*/*w*) with 2.5% **2S-P3-CF770**	20 mM phosphate (pH 7.2)	Bilayer vesicles/LNP

Phospholipids = DSPC:DSPE:Chol (3:1:1).

**Table 2:** pH dependence of liposome stability ^a^.

Liposome	Average Size (d.nm)			
	pH 4.5	pH 7.0		
		0 h	2 h	5 h
**2S-P11-NH_2_**/Chol (4:1 *w*/*w*)	170	164	173	221
**2S-P11-NH_2_**/DPPC-Chol (0.8:3.2:1 *w*/*w*)	317	291	702	-
DPPC-Chol (4:1 *w*/*w*)	211	225	-	224

^a^ The average sizes were obtained from three measurements.

**Table 3:** Liposome formulations tested in an in vivo biodistribution study ^a^.

Exp.	Liposome	Size (id-nm) ^b^	Original Particles/mL	Fluorescent Intensity	Original Concentration (mg/mL)	Adjusted Concentration (mg/mL) ^c^
No. 1	Phospholipids	114.0	2.9 × 10^12^	2.08 × 10^9^	4	2.56
	Phospholipids/**2S-P1000** (1:1 *w*/*w*)	161.8	1.7 × 10^12^	1.33 × 10^9^	4	4.00
	Phospholipids/**2S-P3-Sia** (1:1 *w*/*w*)	148	3.3 × 10^12^	6.29 × 10^9^	4	0.85
No. 2	Phospholipids	245.8	4.2 × 10^13^	5.73 × 10^9^	5	3.35
	Phospholipids/**2S-P1000** (3:1 *w*/*w*)	241.9	6.3 × 10^11^	7.61 × 10^9^	5	2.53
	Phospholipids/**2S-P3-Sia** (3:1 *w*/*w*)	203.8	5.7 × 10^11^	3.84 × 10^9^	5	5.00

^a^ Phospholipids are DSPC:DSPE:Chol (3:1:1, *w*/*w*); ^b^ The average sizes from three measurements prior to in vivo experiments; ^c^ The concentrations after fluorescent intensity adjustment.

## Data Availability

The authors declare that, in addition to the data and images provided as supporting material, other raw data supporting the findings are available upon request to the corresponding author.

## Supplementary Materials

The following supporting information can be downloaded at: https://www.mdpi.com/article/10.3390/ijms26189176/s1.

## Abbreviations

PMBN	polymyxin B nonapeptide
DCC	*N*,*N*′-dicyclohexylcarbodiimide
NHS	*N*-hydroxysuccinimide
SUV	Small unilamellar vesicle
LUV	Large unilamellar vesicle
MLV	Multilamellar vesicle
DSPC	Distearoylphosphatidylcholine
DSPE	1,2-Distearoyl-sn-glycero-3-phosphoethanolamine
DPPC	Dipalmitoyl phosphatidylcholine
Chol	Cholesterol
DLS	Dynamic Light Scattering
Cryo-TEM	Cryogenic transmission electron microscopy
