# Supplementary material for: Synthesis of α,ε-N,N′-Di-stearoyl Lysine-Derived Amide Lipids and Their Application to Liposome Formulation: Incorporation of Lipid A-Ligand for Bacterial Targeting and Sialic Acid for Phagocytosis Resistance
Source: Int J Mol Sci. 2025 Sep 19;26(18):9176. doi: 10.3390/ijms26189176 (PMC12470916; doi:10.3390/ijms26189176)
Supplement: Supplementary file 1 [file ijms-26-09176-s001.zip › ijms-3760743-supplemetary file - Dean...Zou-SI-revised.pdf]

Supplementary Material

Synthesis of α,ε-*N,N'*-di-stearoyl Lysine-derived Amide Lipids and Their Application to Liposome Formulation: Incorporation of Lipid A-ligand for Bacterial Targeting and Sialic Acid for Phagocytosis Resistance

Dean Williams,[1] Alyssa McAdorey,[1,2] Eric Lei,[1] Greg Beaudoin,[1] Binbing Ling,[1] Debbie Callaghan,[1] Dorothy Fatehi,[1] Angie Verner,[1] Jacqueline Slinn,[1] Maria Moreno,[1] Umar Iqbal,[1] Hui Qian,[3] Hongbin Yan,[2] Wangxue Chen,[1,2] Wei Zou[1*]

[1] *Human Health Therapeutics Research Centre, National Research Council Canada, 100 Sussex Drive, Ottawa, Ontario, Canada K1A 0R6*

[2] *Department of Chemistry and Centre for Biotechnology, Brock University, 1812 Sir Isaac Brock Way, St. Catharines, Ontario, Canada L2S 3A1*

[3] *Quantum and Nanotechnologies Research Center, National Research Council Canada, 11421 Saskatchewan Drive NW, Edmonton, AB T6G 2M9*

*Correspondance: wei.zou@nrc-cnrc.gc.ca

Contents

1. Figure S1 - NMR spectra of compounds synthesized
2. Table S1 - DLS and zeta potential analysis of liposomes
3. Table S2 - The internalization of nanoparticle-FITC into target cells (median fluorescent intensity)
4. Figure S2 - First in vivo imaging experiment
5. Figure S3 – First ex vivo Imaging IV (tail vein)
6. Figure S4 - Second in vivo imaging experiment
7. Figure S5 – Second ex vivo Imaging IV (tail vein)

Figure S1 - NMR spectra of compounds synthesized

^{1}H NMR spectrum (500 MHz, $CDCl_3$) of compound **1**

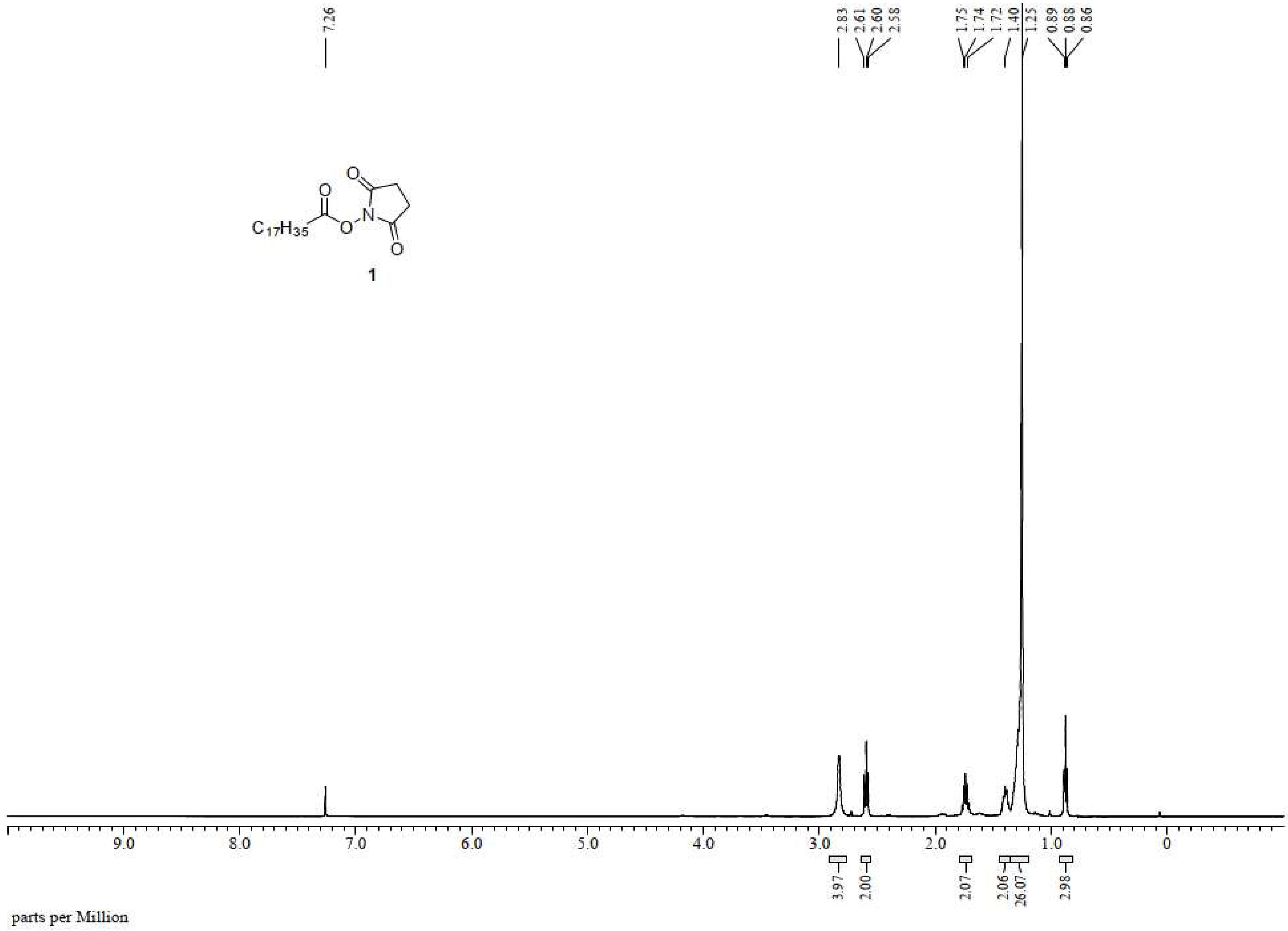

$^{13}C\{^1H\}$ NMR spectrum (125 MHz, $CDCl_3$) of compound **1**

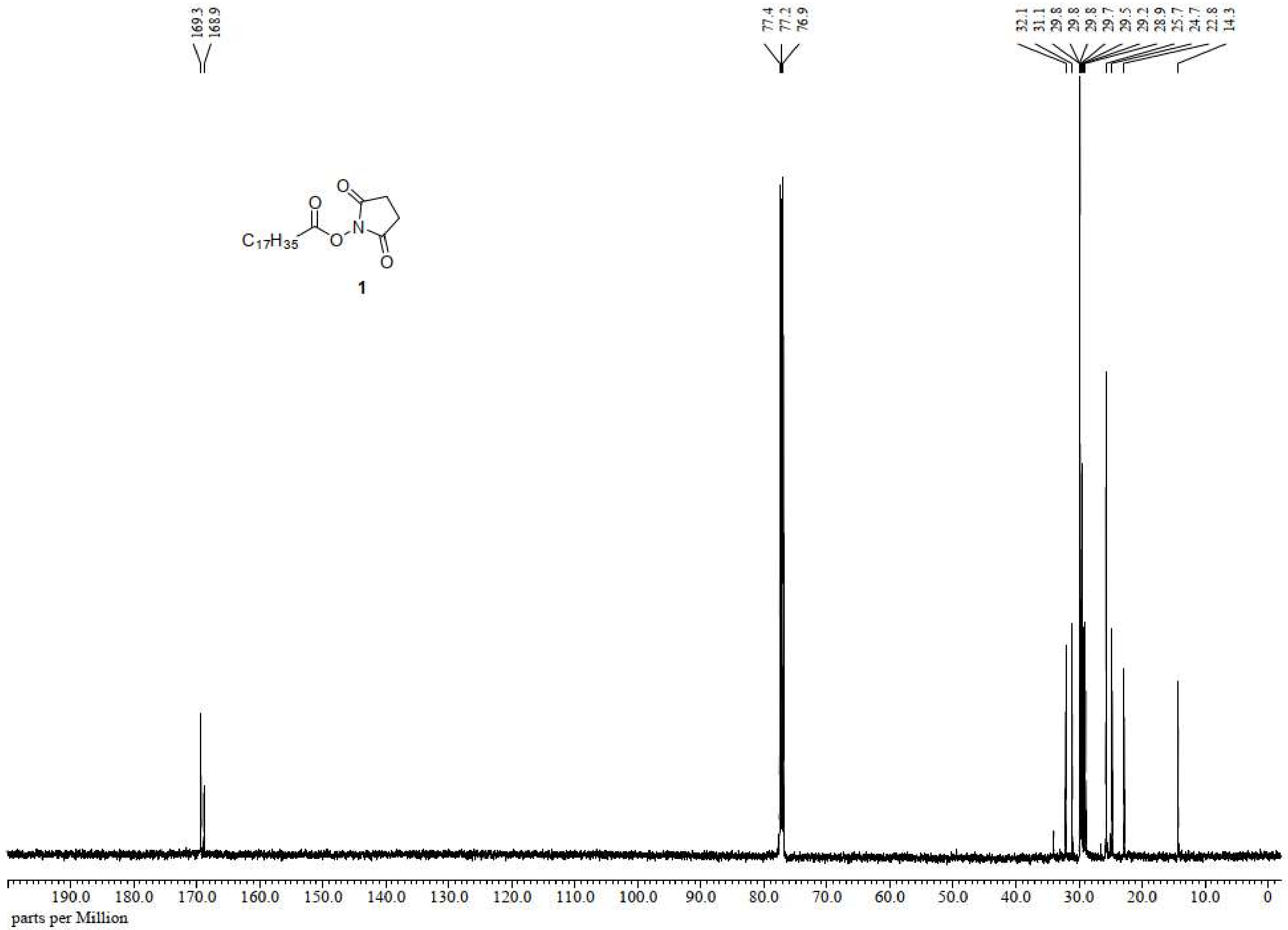

^{1}H NMR spectrum (500 MHz, 4:3 $CDCl_3$:CD_3OD) of compound **2**

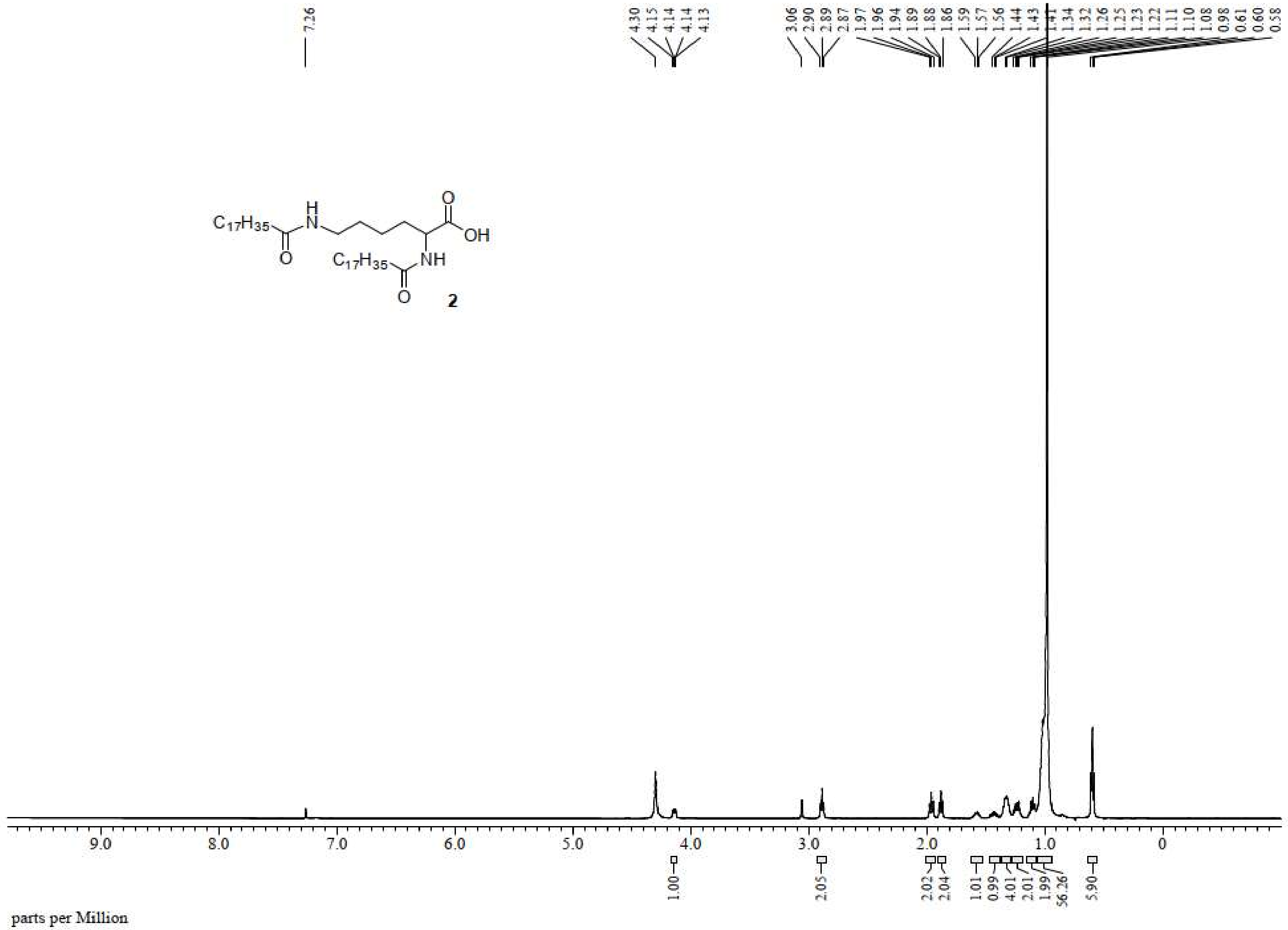

$^{13}C\{^1H\}$ NMR spectrum (125 MHz, 4:3 $CDCl_3$:CD_3OD) of compound **2**

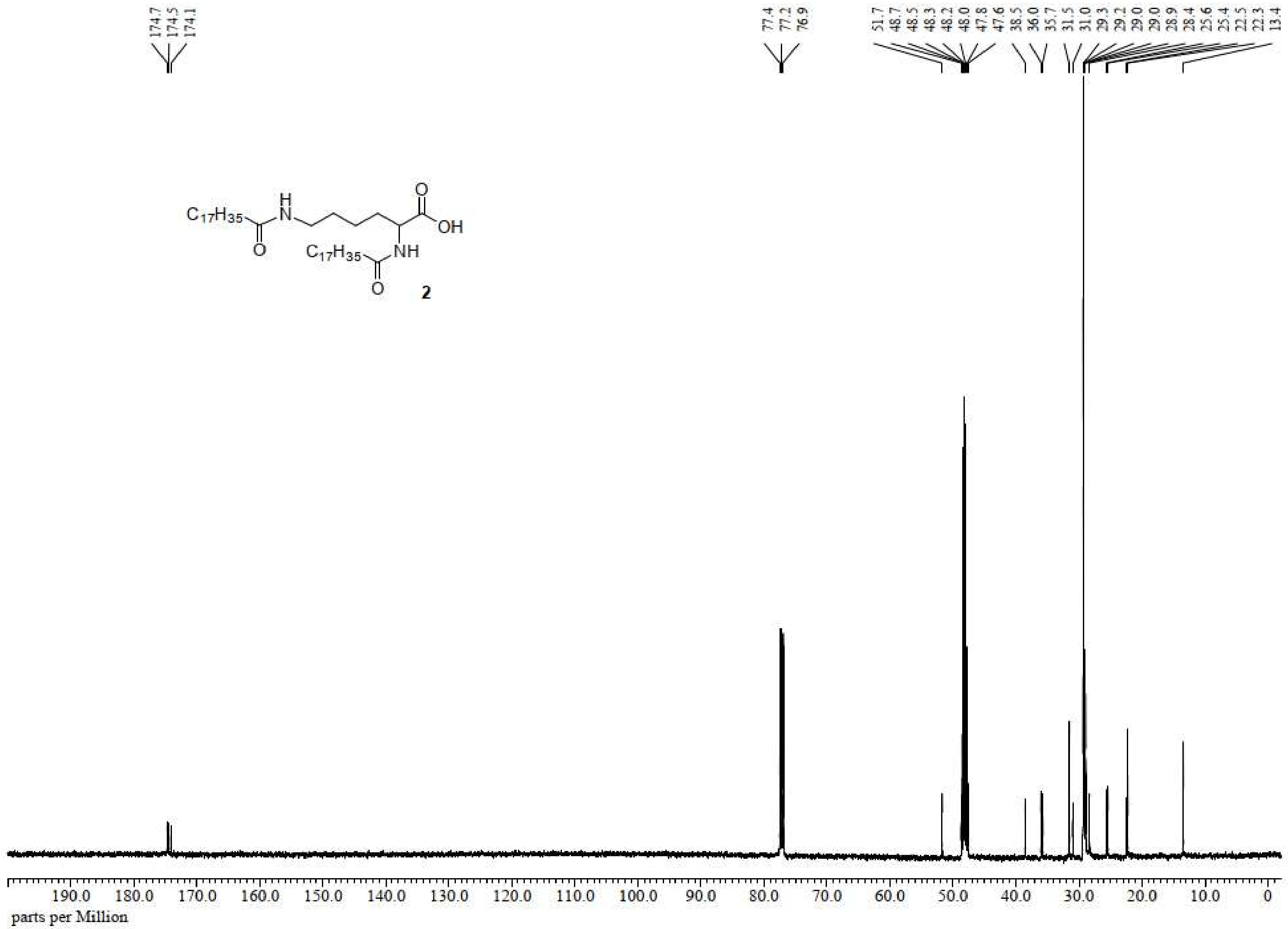

^{1}H NMR spectrum (500 MHz, $CDCl_3$, 35°C) of compound **3**

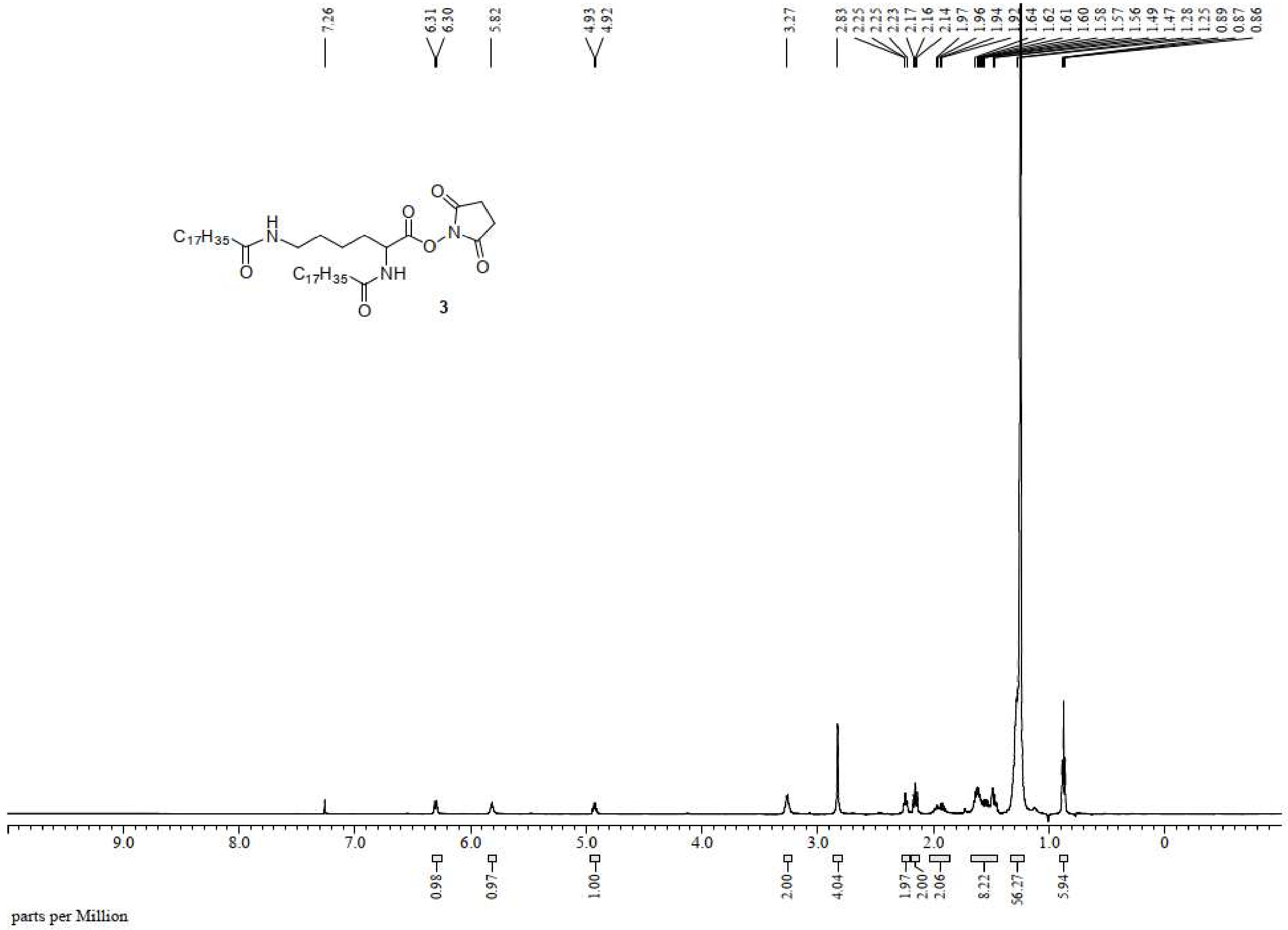

$^{13}C\{^{1}H\}$ NMR spectrum (125 MHz, $CDCl_3$, 35°C) of compound **3**

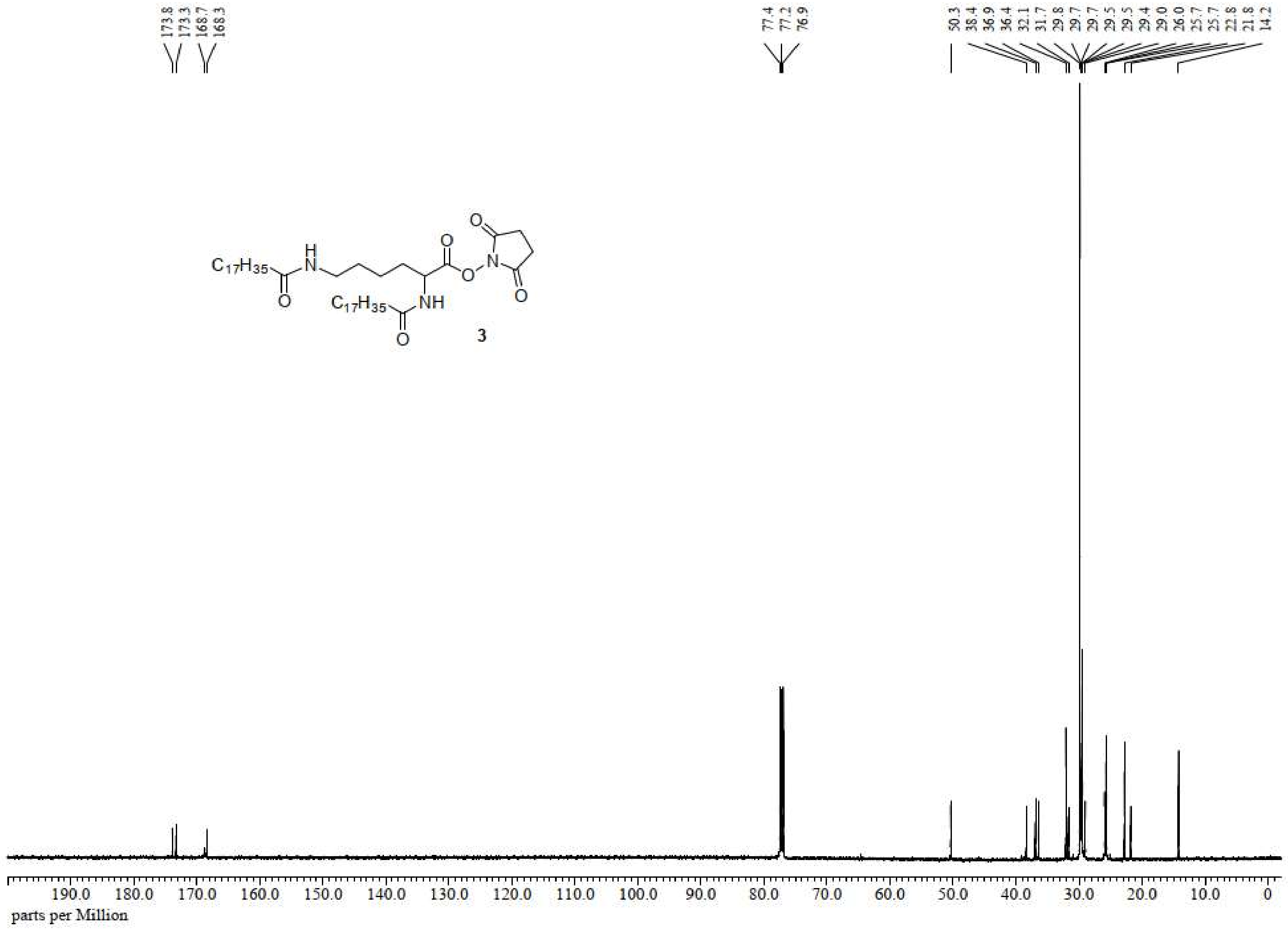

^{1}H NMR spectrum (400 MHz, 2:1 $CDCl_3$:CD_3OD) of compound **4**

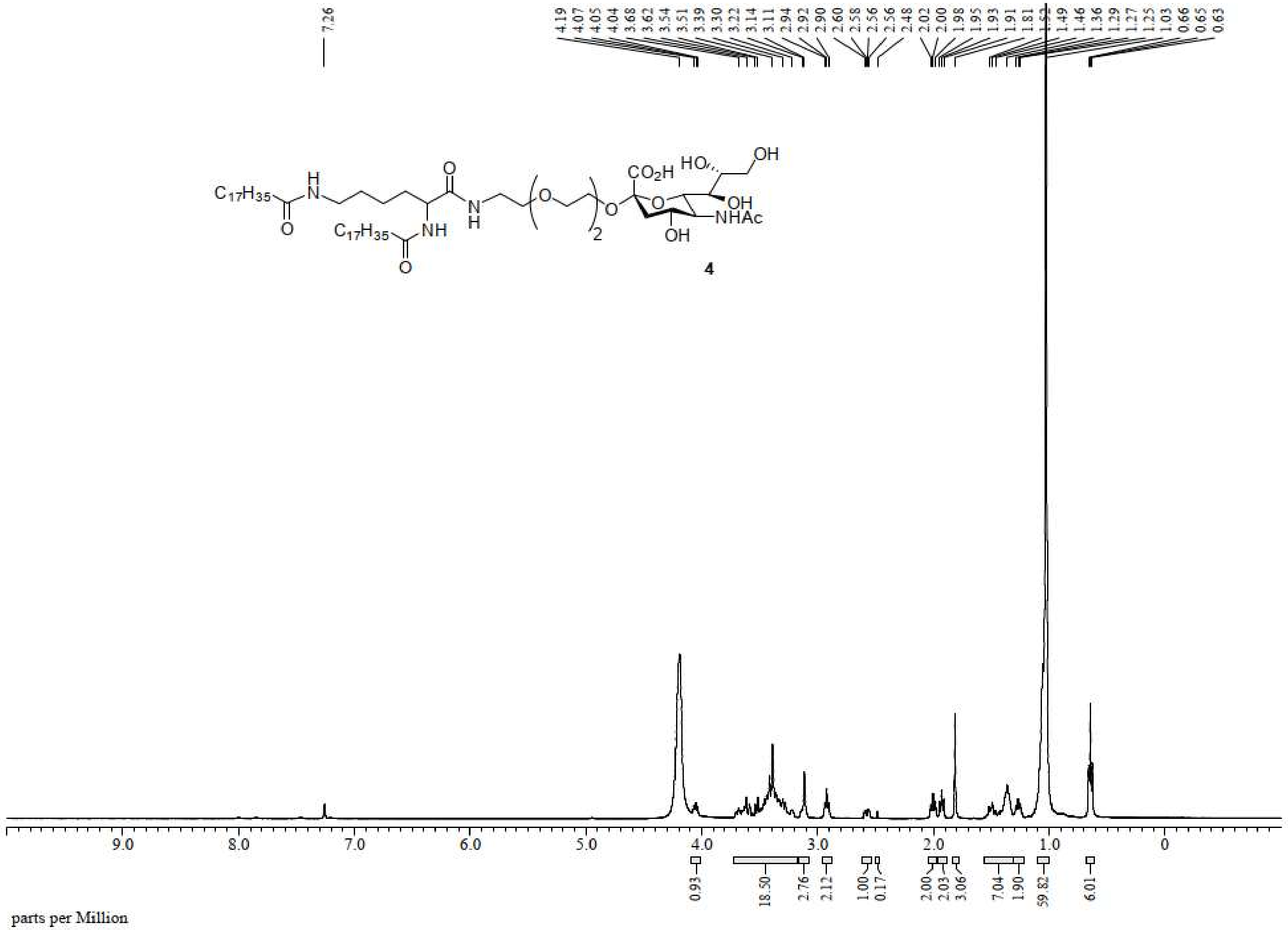

$^{13}C\{^1H\}$ NMR spectrum (101 MHz, 2:1 $CDCl_3$:CD_3OD) of compound **4**

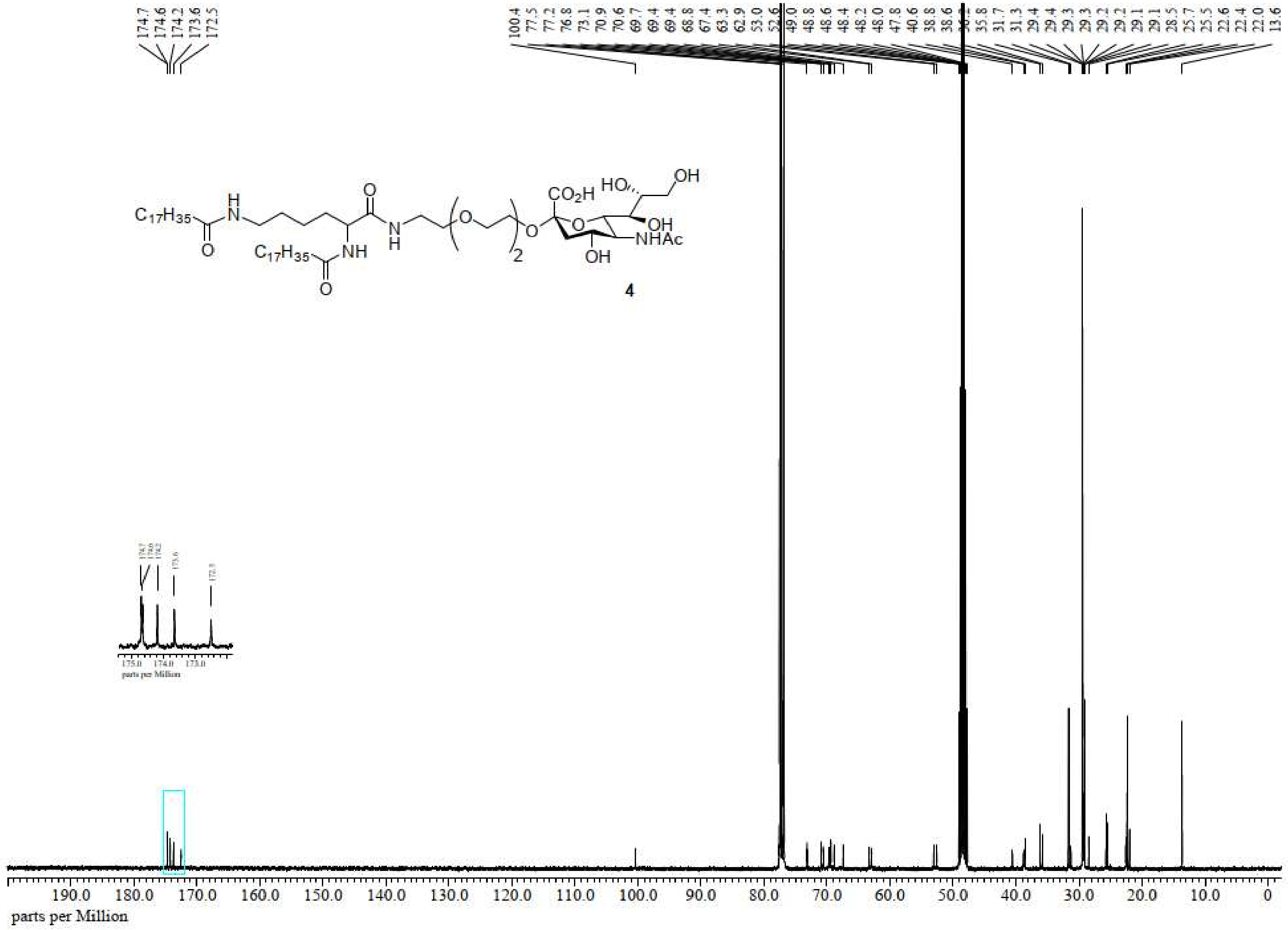

^{1}H NMR spectrum (400 MHz, $CDCl_3$) of compound **5**

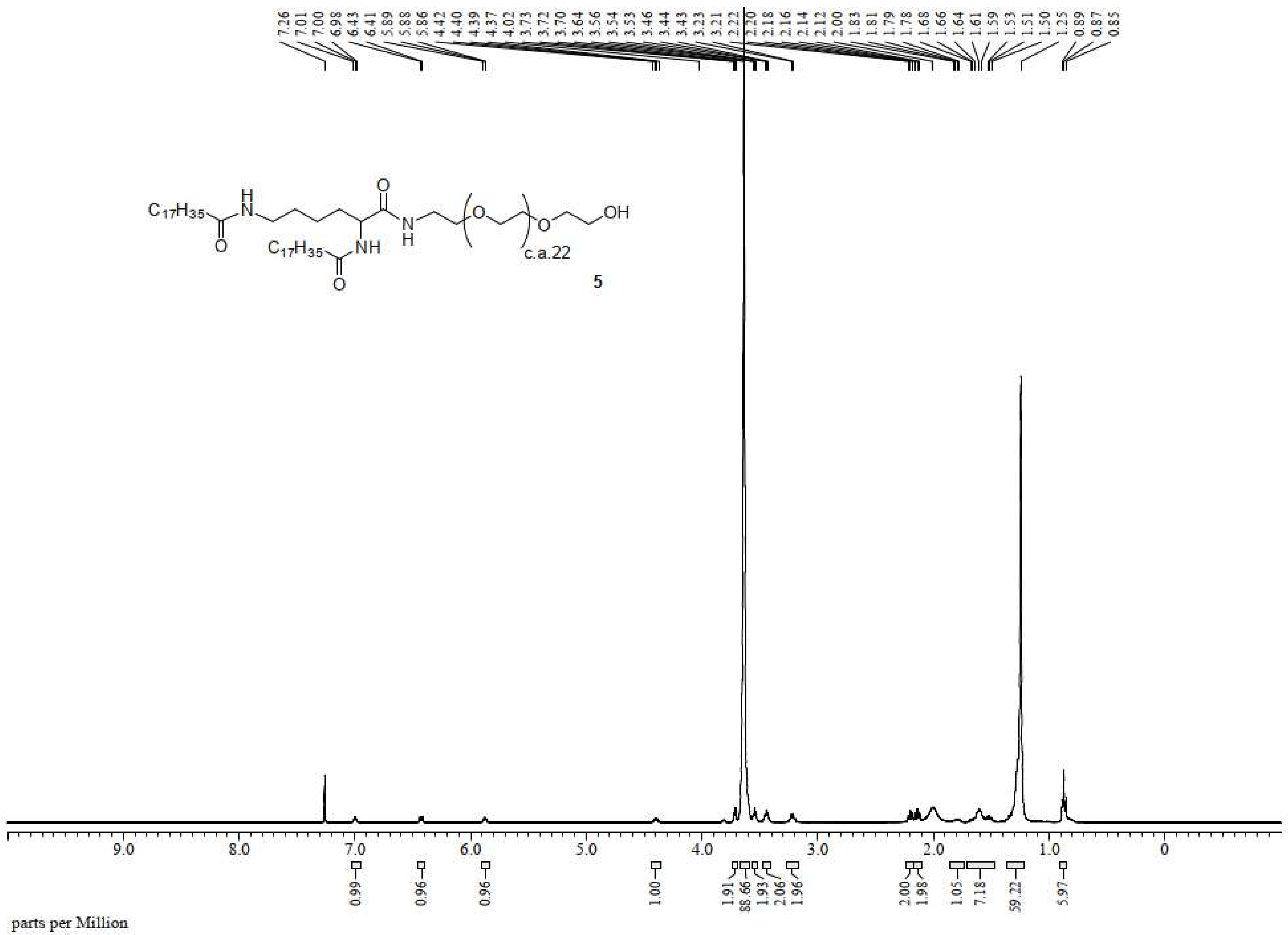

5

$^{13}C\{^1H\}$ NMR spectrum (101 MHz, $CDCl_3$) of compound **5**

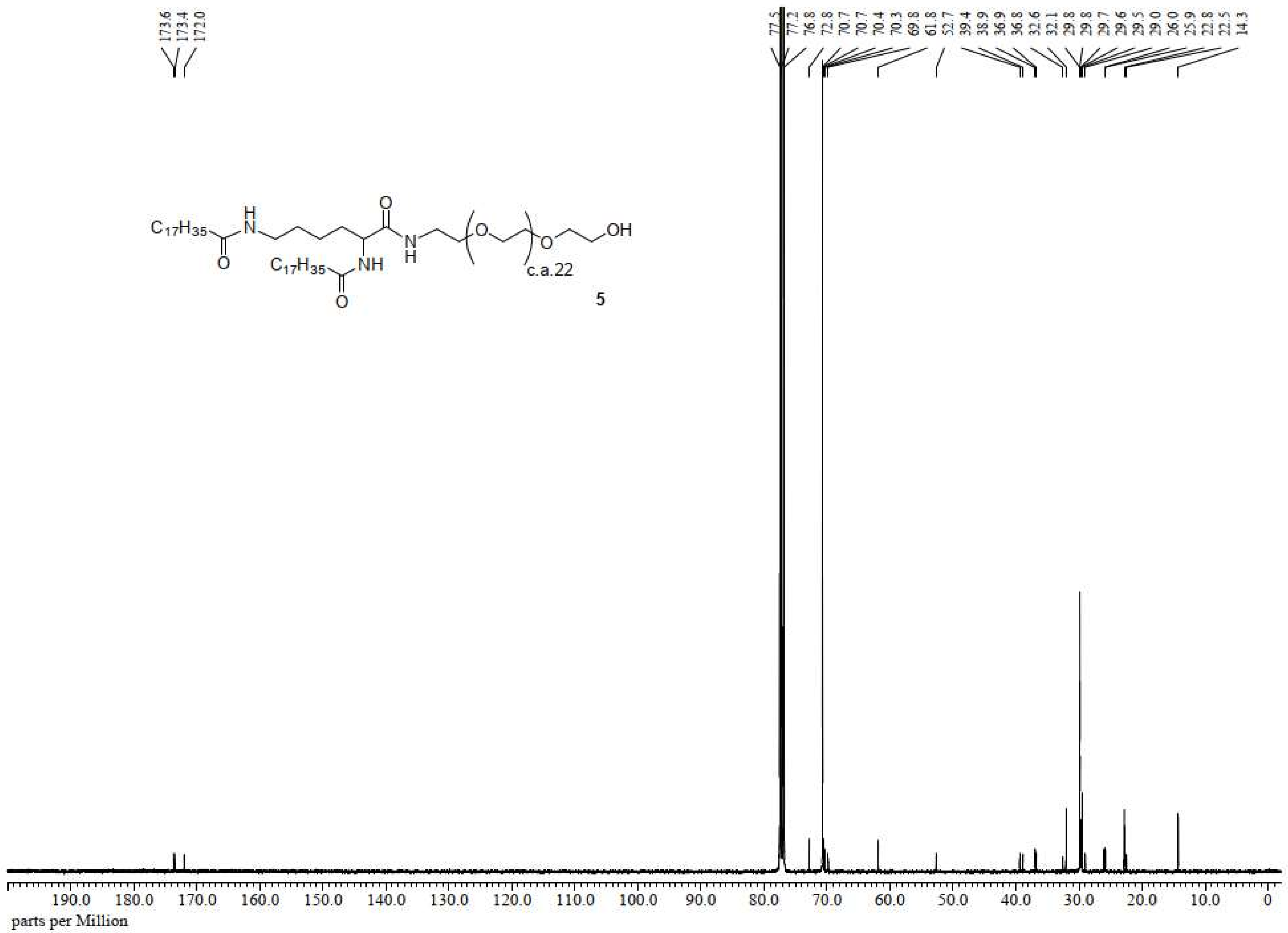

^{1}H NMR spectrum (500 MHz, $CDCl_3$) of compound **6**

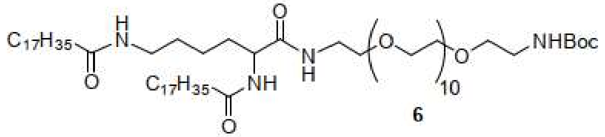

6

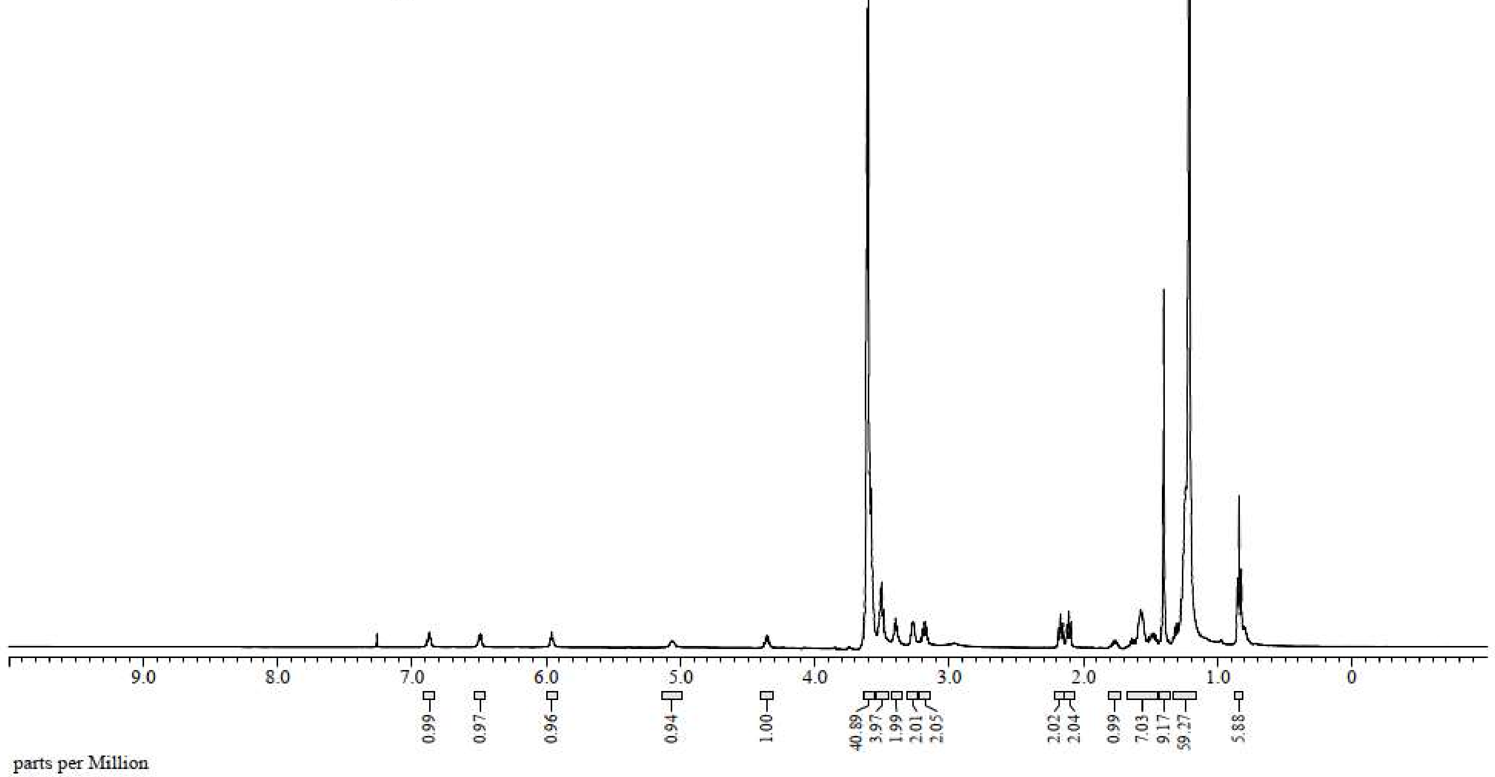

$^{13}C\{^{1}H\}$ NMR spectrum (125 MHz, $CDCl_3$) of compound **6**

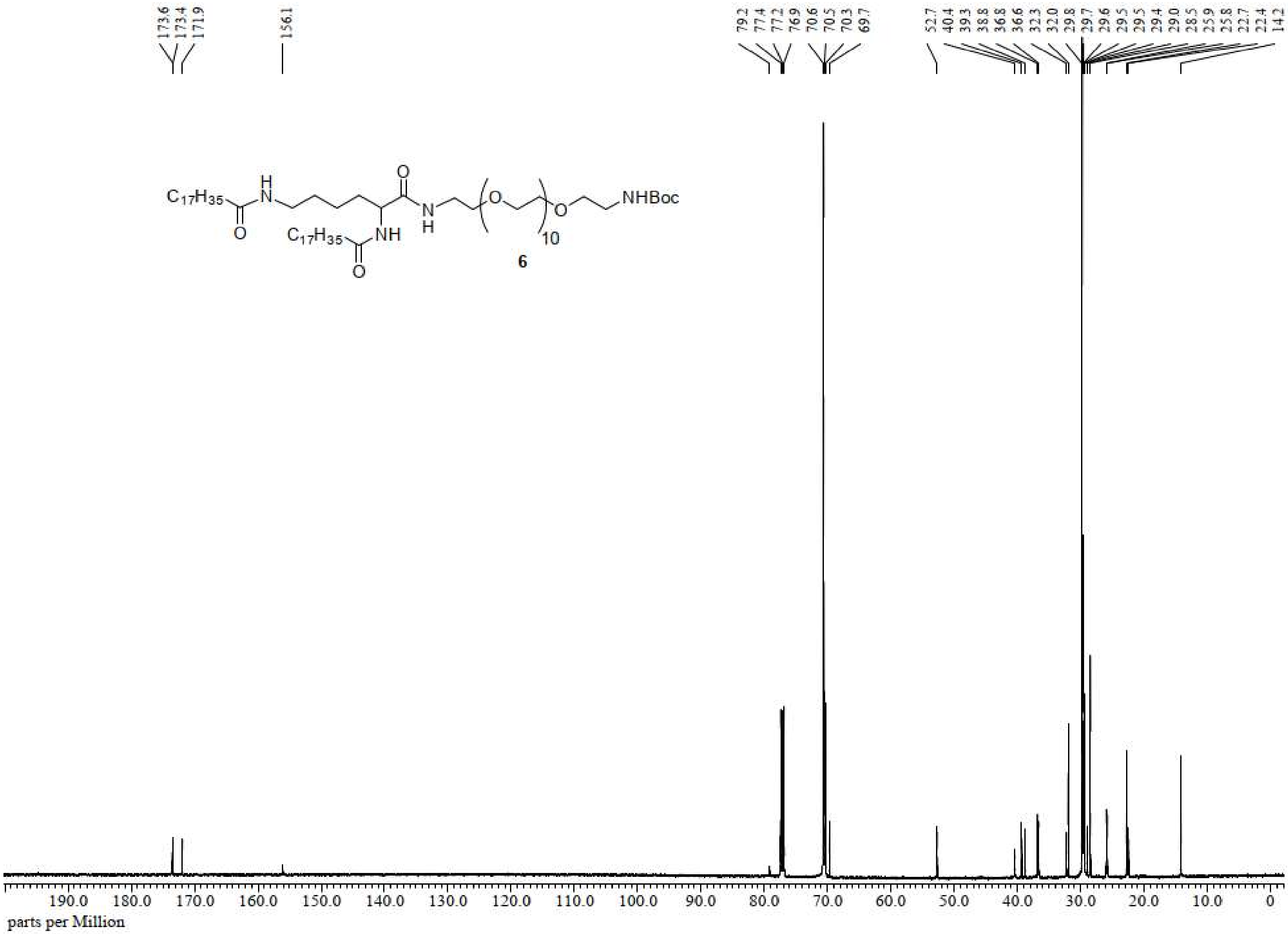

^{1}H NMR spectrum (400 MHz, $CDCl_3$) of compound **7**

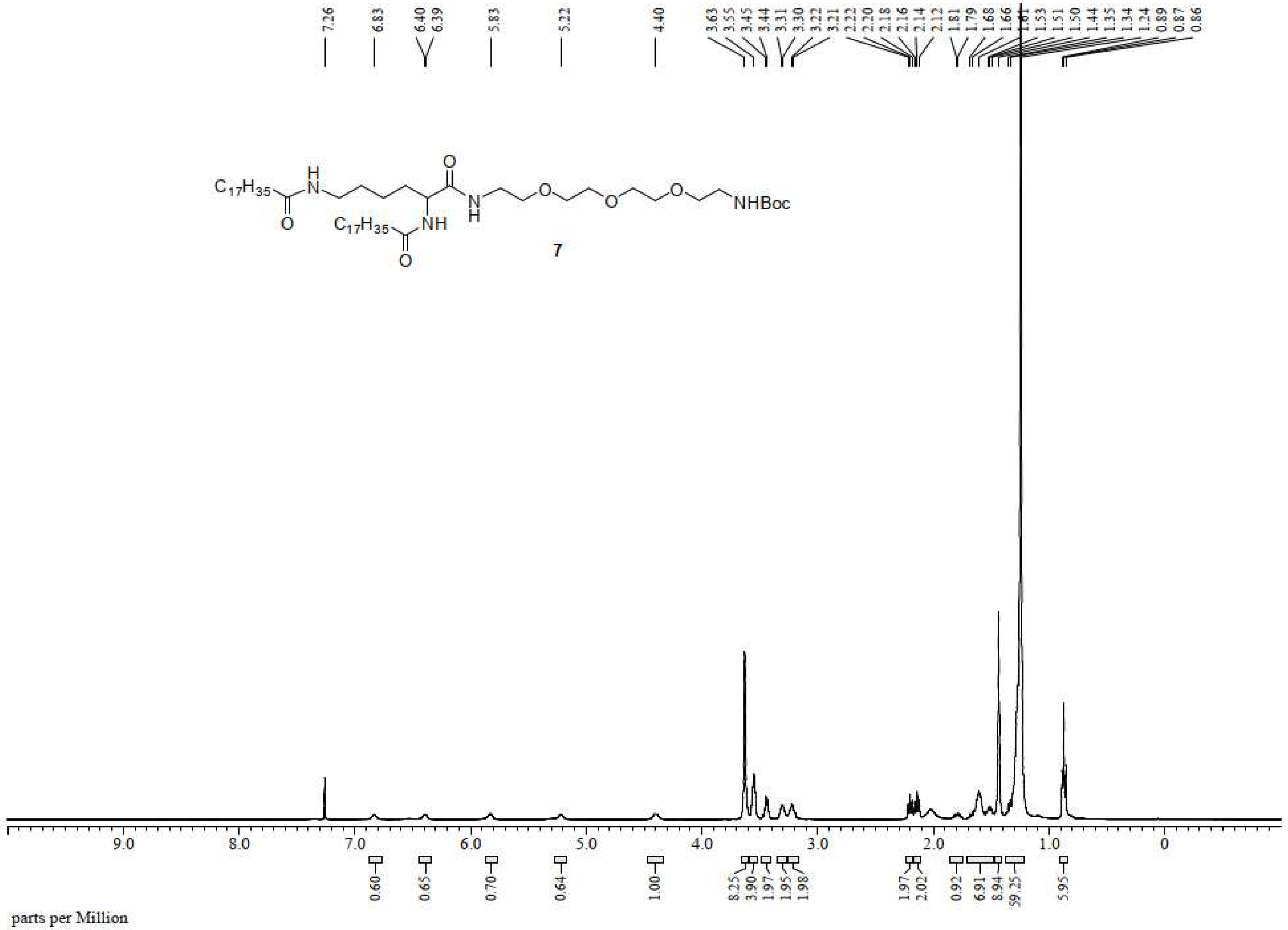

$^{13}C\{^{1}H\}$ NMR spectrum (101 MHz, $CDCl_3$) of compound **7**

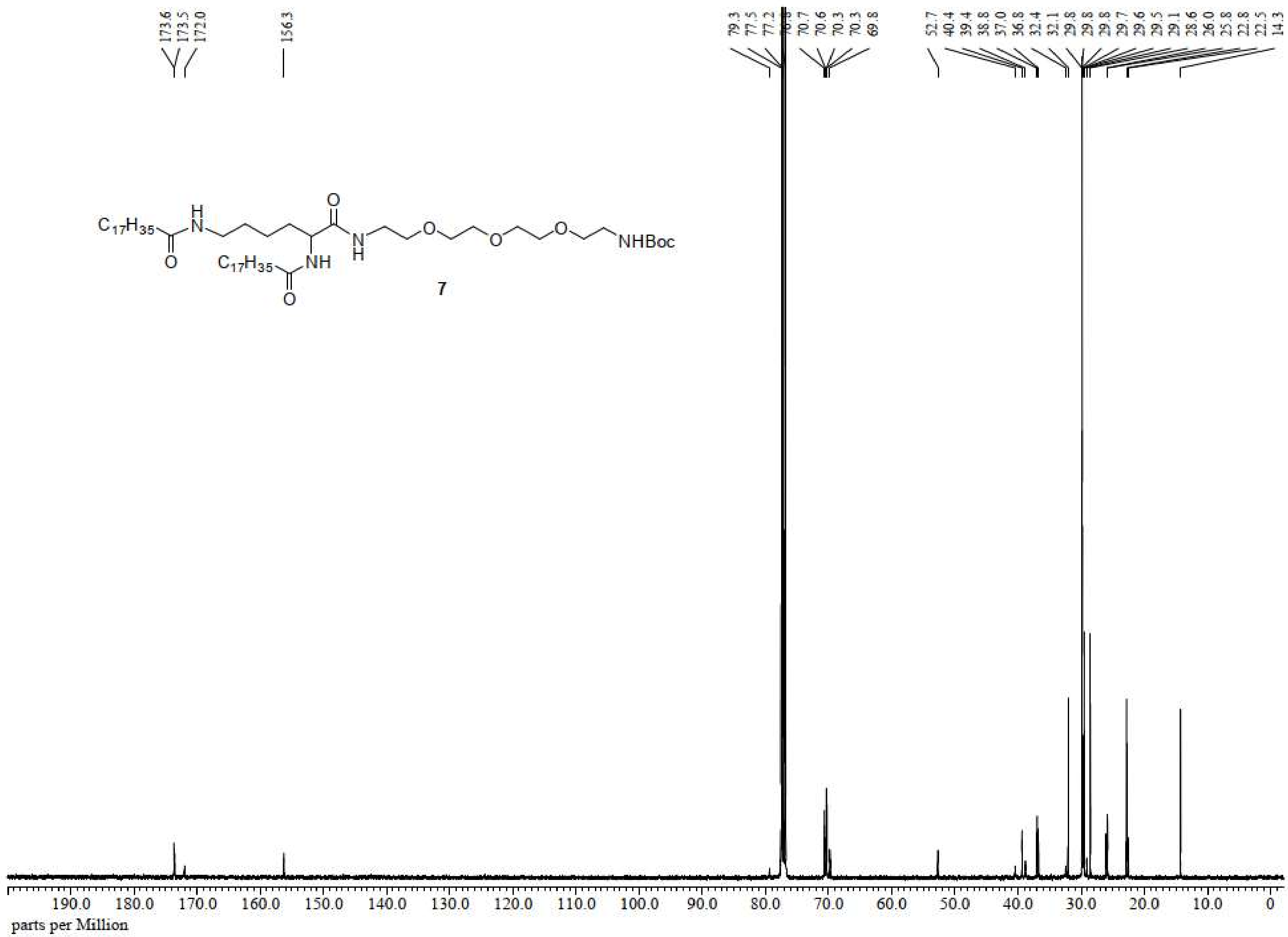

^{1}H NMR spectrum (500 MHz, $CDCl_3$) of compound **8**

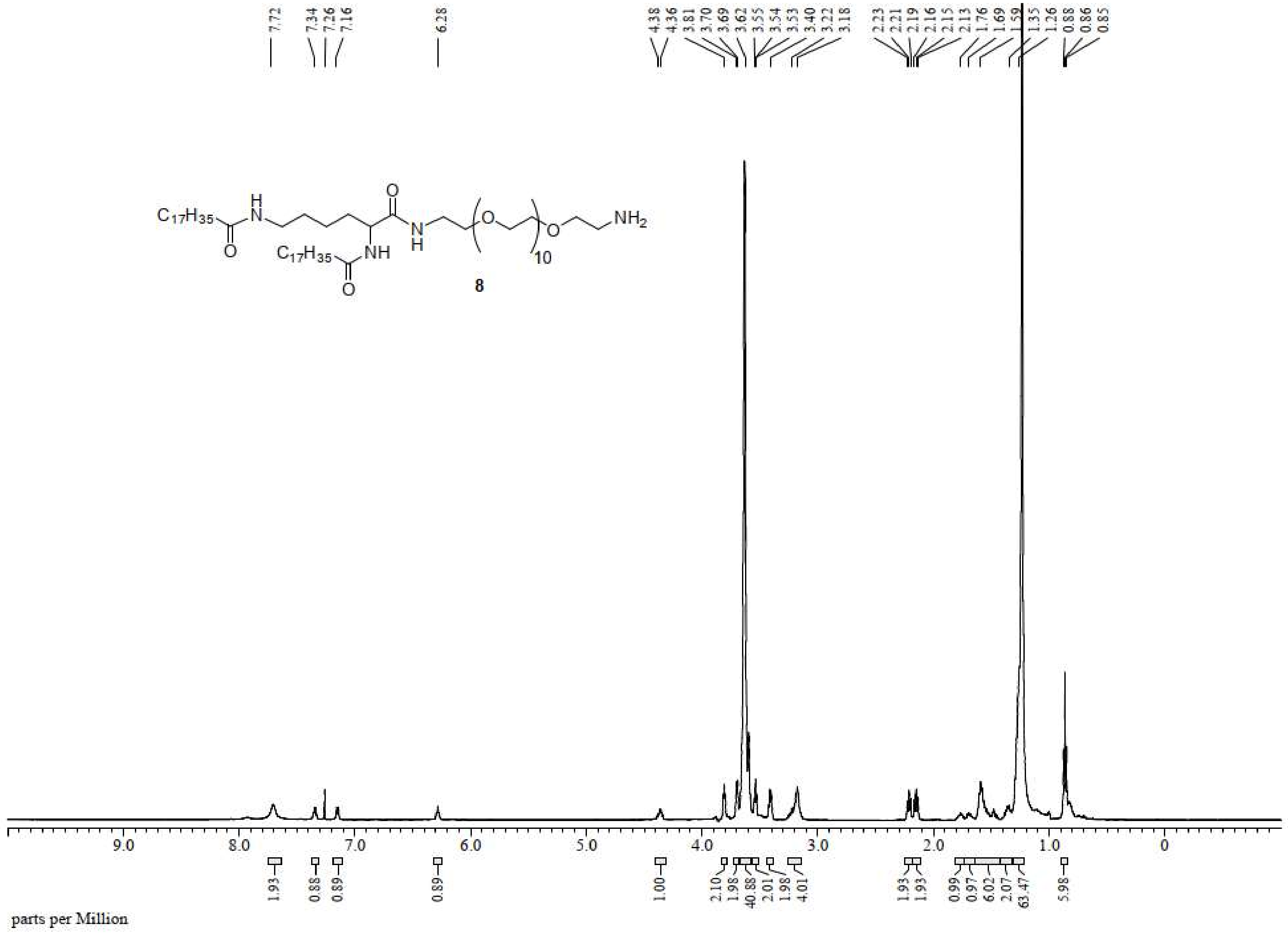

$^{13}C\{^1H\}$ NMR spectrum (125 MHz, $CDCl_3$) of compound **8**

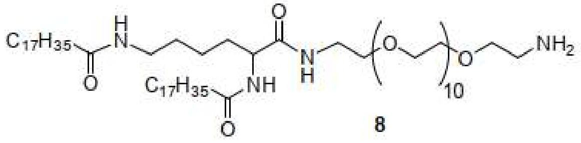

8

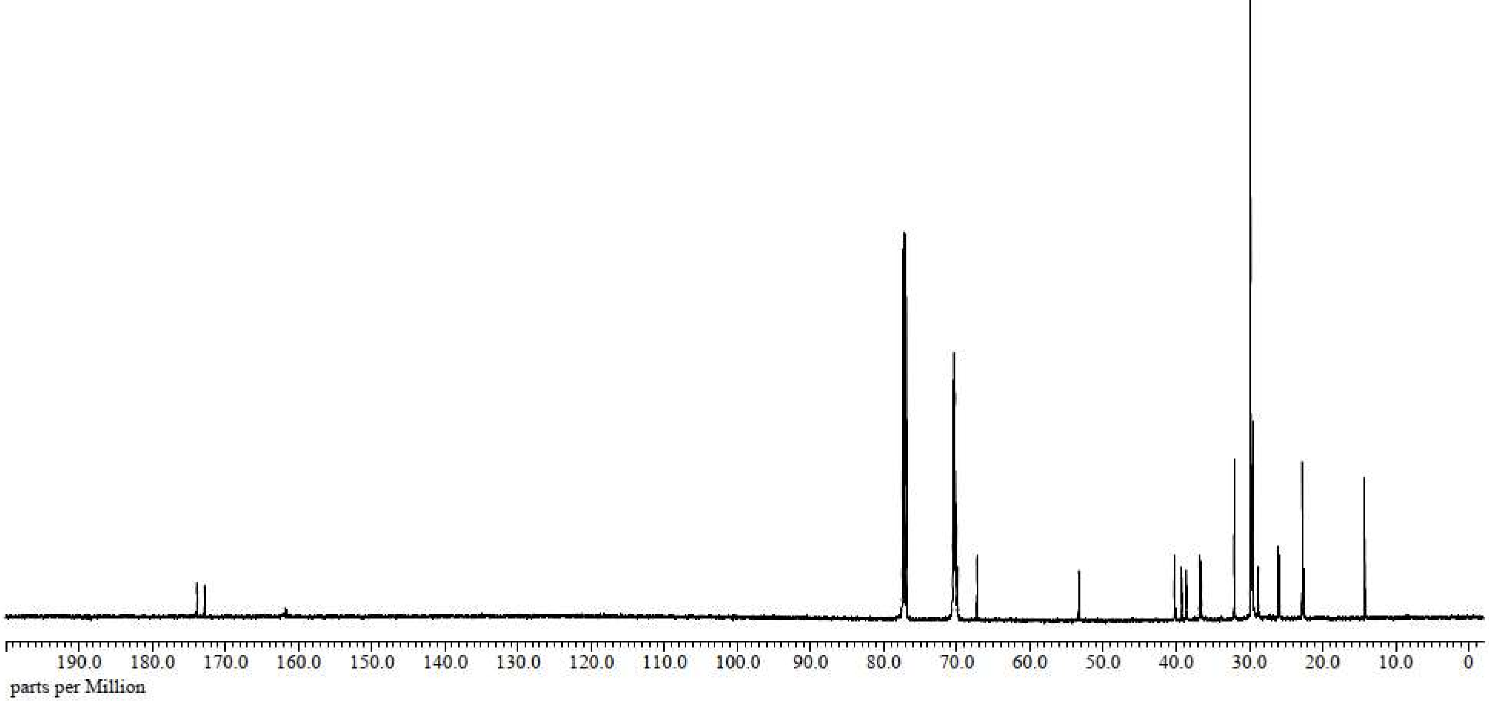

^{1}H NMR spectrum (400 MHz, $CDCl_3$) of compound **9**

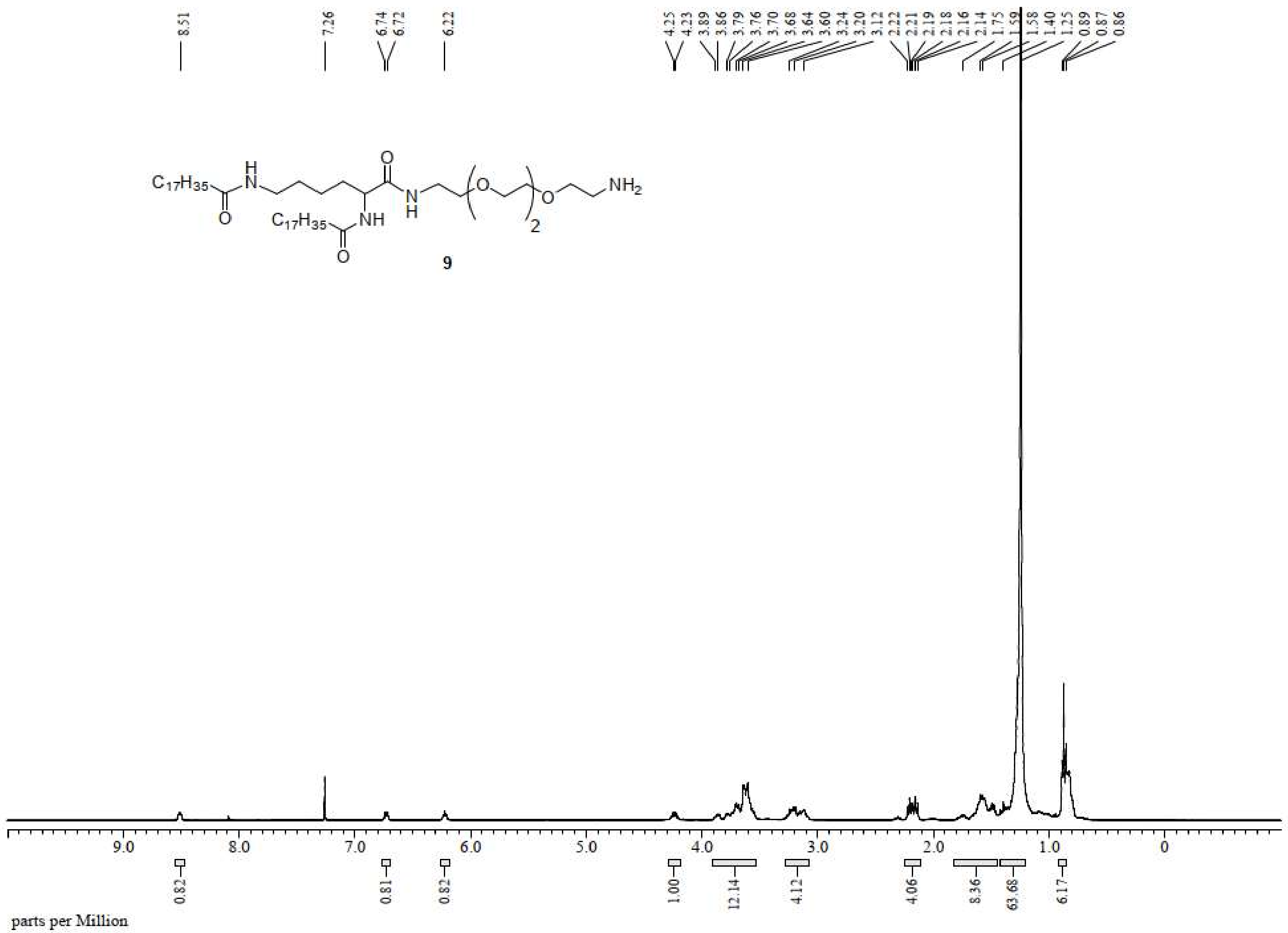

$^{13}C\{^{1}H\}$ NMR spectrum (101 MHz, $CDCl_3$) of compound **9**

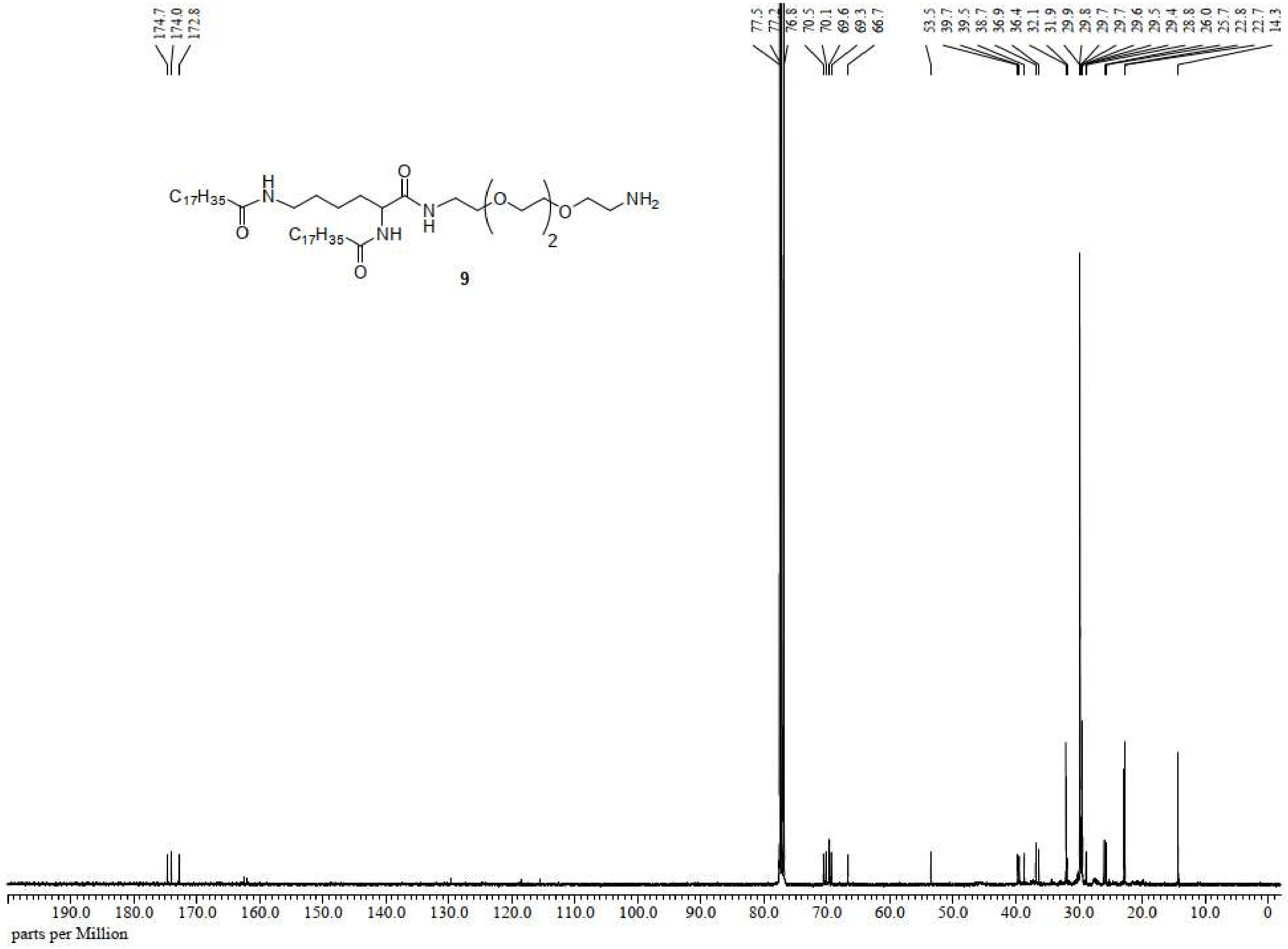

^{1}H NMR spectrum (400 MHz, 2:1 $CDCl_3$:CD_3OD) of compound **10**

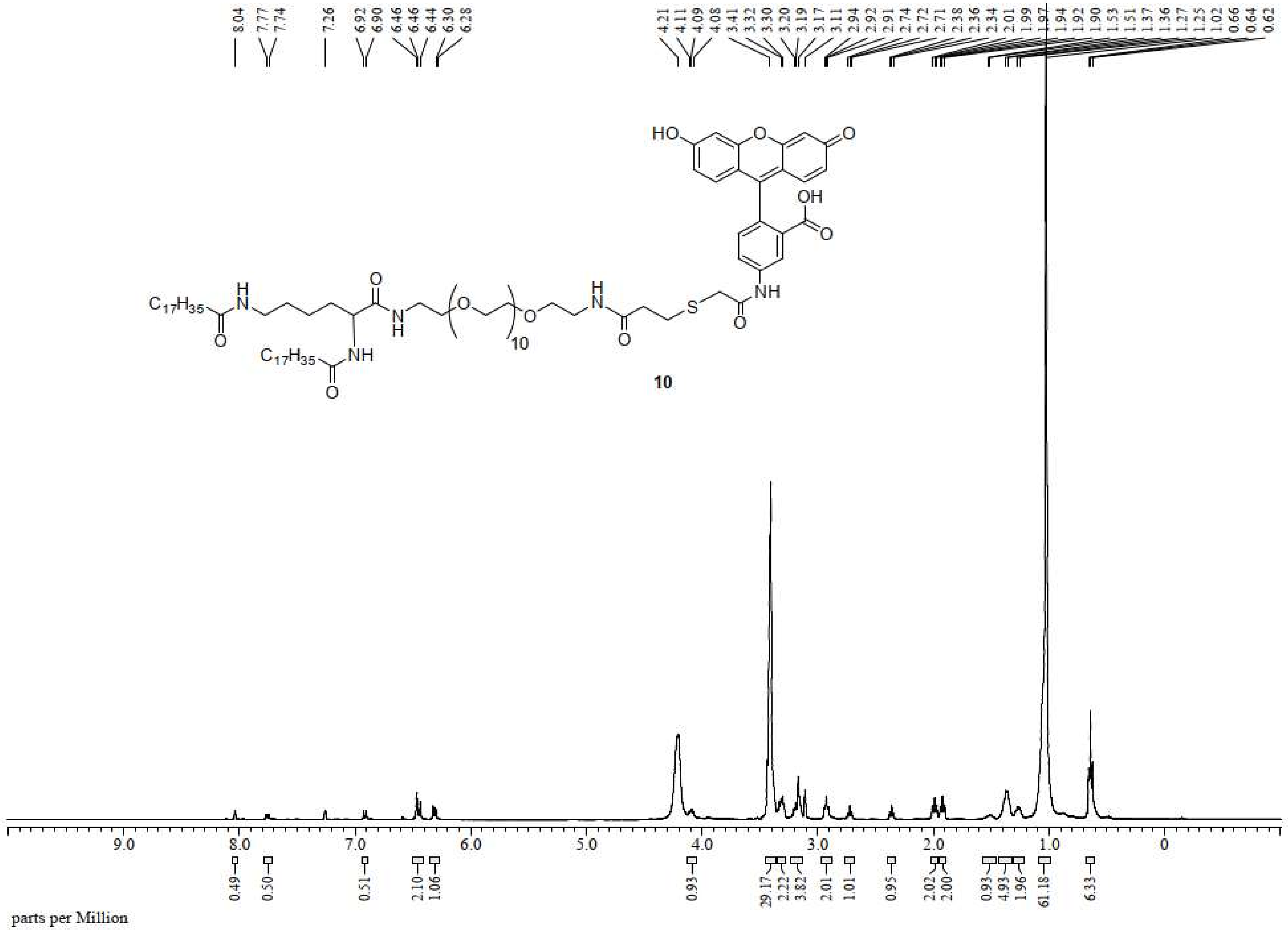

$^{13}C\{^1H\}$ NMR spectrum (101 MHz, 2:1 $CDCl_3$:CD_3OD) of compound **10**

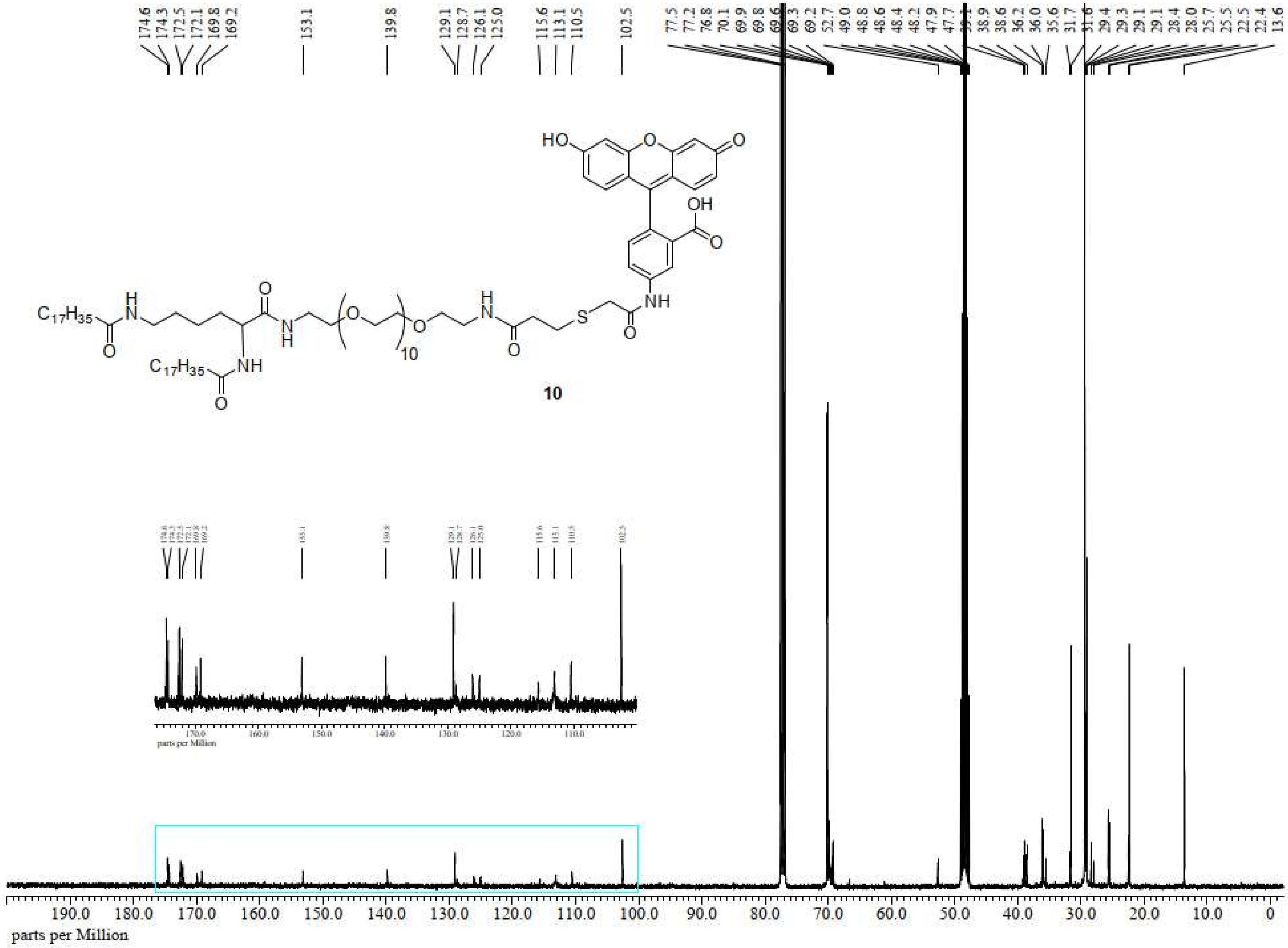

10

MALDI spectrum of compound **11**

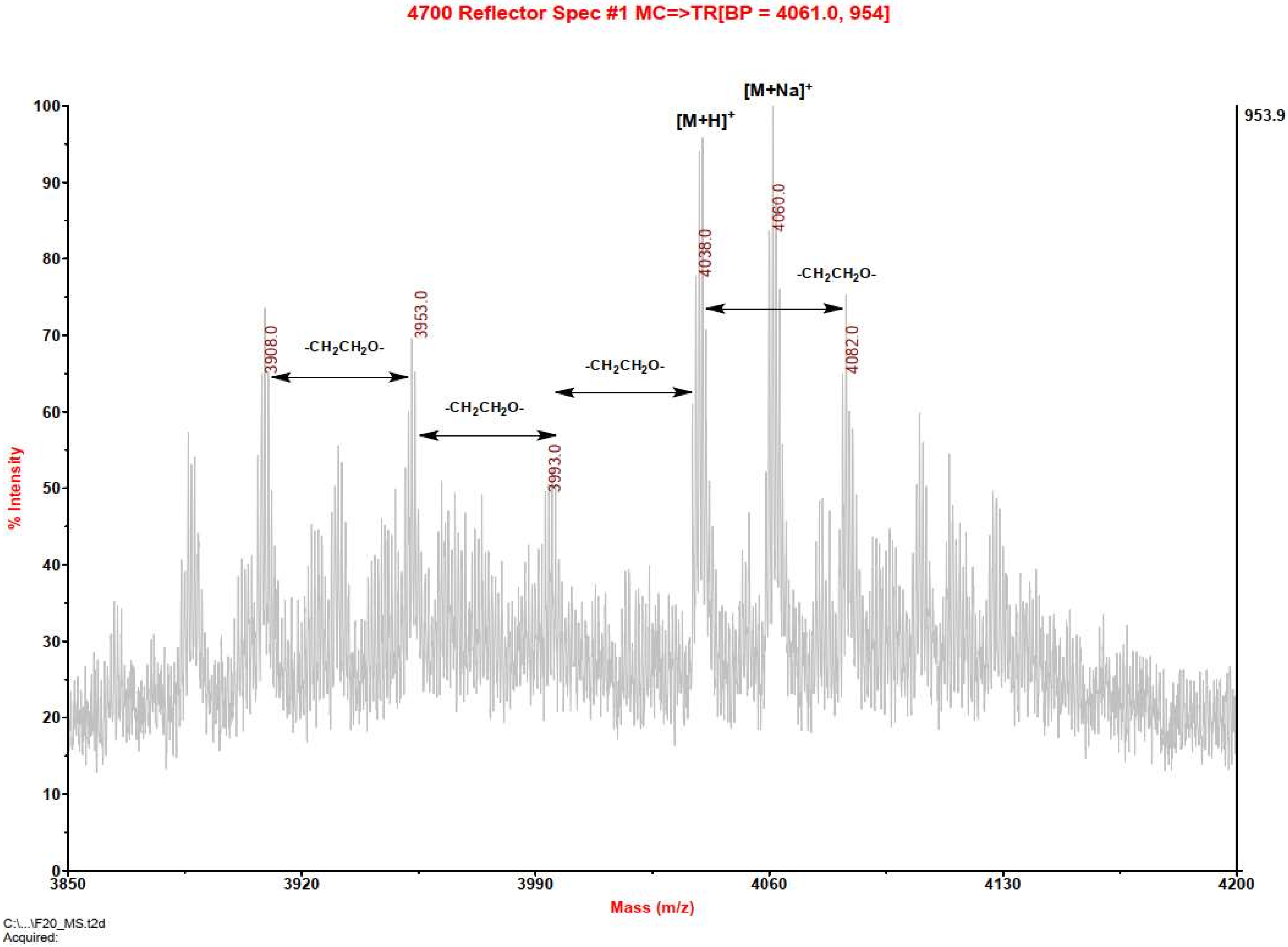

File Name: F20_MS

Trace: 4700 Reflector Spec #1 MC=>TR[BP = 4061.0, 954]

Index	Centroid Mass	Lower Bound	Upper Bound	Charge (z)	Height	Relative Intensity	Area (A)	S/N Ratio	Resolution	Isot
1	3886.01001	3885.59	3886.55	1	518	54.28	3556.62	23.10	9238.01	6083.51
2	3906.94751	3906.56	3907.53	1	507	53.15	2963.22	43.48	11082.31	18145.47
3	3930.99292	3930.61	3931.62	1	516	54.12	2894.64	23.99	9847.92	5965.64
4	3950.94434	3950.58	3951.47	1	503	52.71	2732.94	37.33	11412.60	13955.40
5	3961.91602	3961.40	3962.53	1	475	49.84	3083.24	15.64	12164.92	3076.49
6	3992.96533	3992.59	3993.52	1	471	49.33	3289.38	26.54	11485.38	8534.96
7	4037.00806	4036.50	4037.60	1	550	57.66	3833.99	47.18	11685.93	24268.70
8	4059.02905	4058.47	4059.57	1	489	51.30	4344.42	37.79	9080.17	26333.30
9	4075.93799	4075.65	4076.56	1	471	49.39	2738.79	23.30	11212.25	5866.40
10	4081.97388	4081.56	4082.58	1	611	64.03	5259.56	36.26	8609.56	13354.75
11	4104.95264	4104.52	4105.58	1	558	58.53	3609.50	25.48	12473.21	6659.27
12	4113.91797	4113.41	4114.40	1	484	50.79	2819.24	16.44	13373.50	2684.09

^{1}H NMR spectrum (500 MHz, $CDCl_3$) of compound **12**

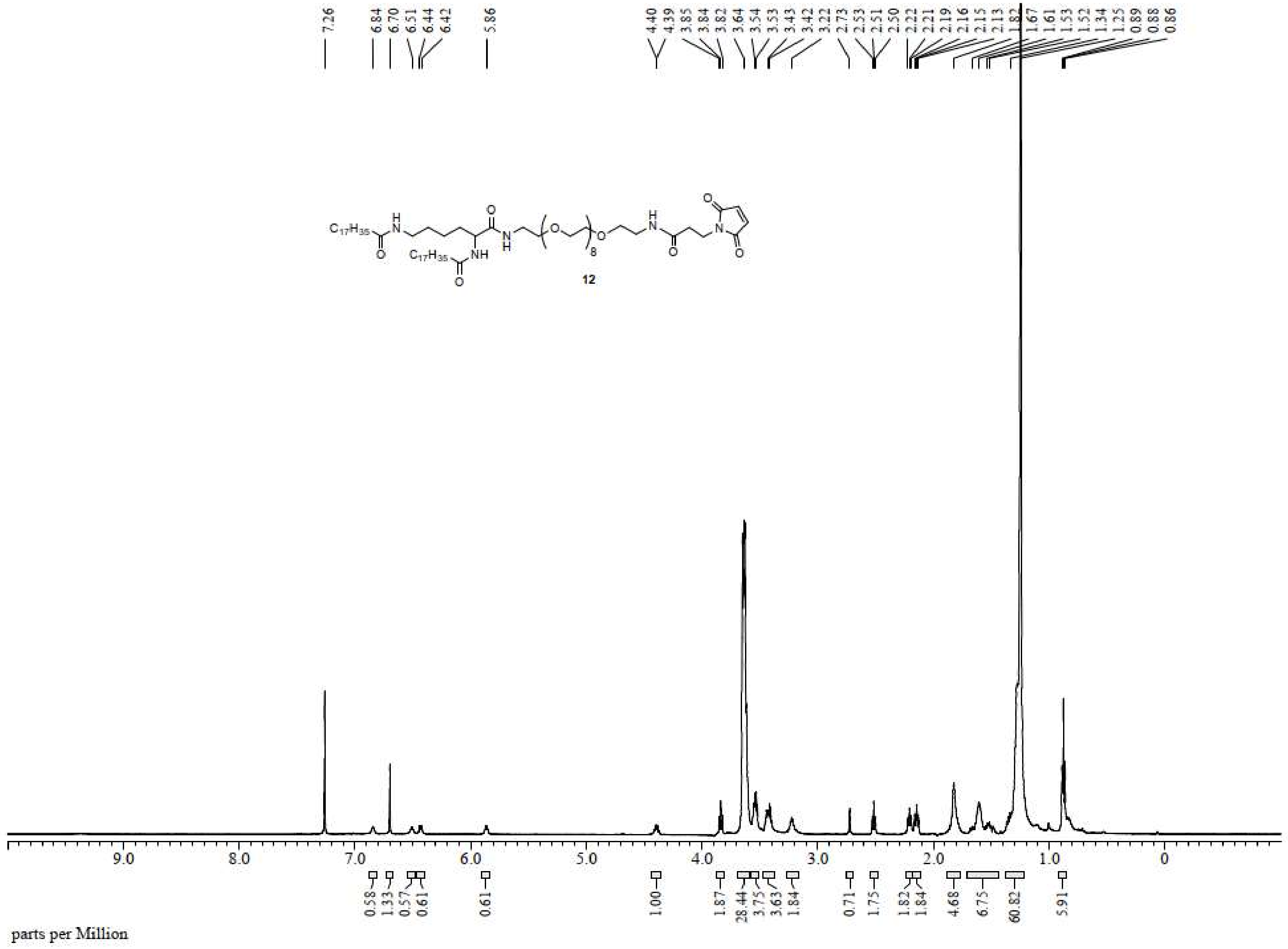

$^{13}C\{^{1}H\}$ NMR spectrum (125 MHz, $CDCl_3$) of compound **12**

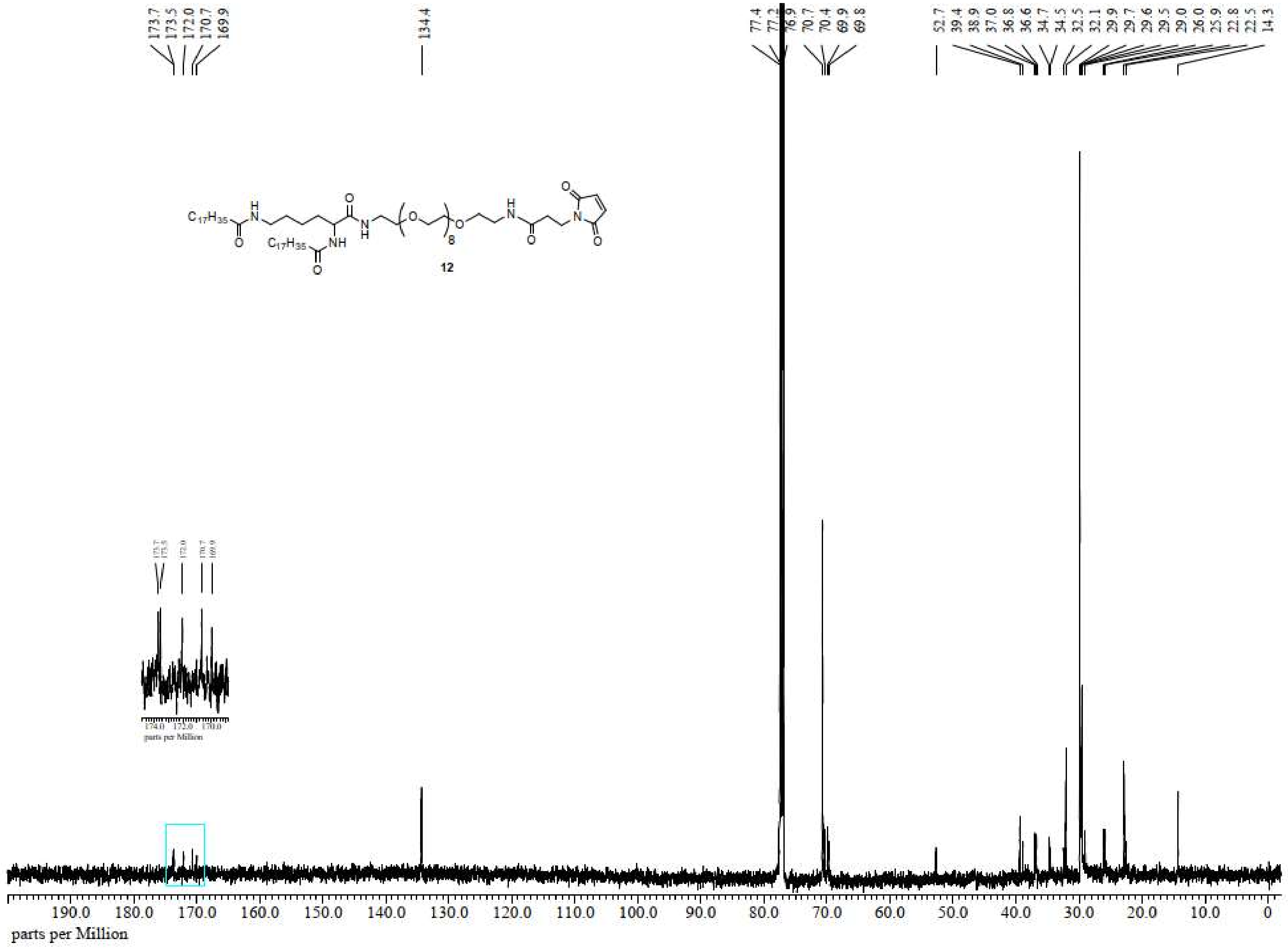

^{1}H NMR spectrum (500 MHz, 1:1 $CDCl_3$:CD_3OD) of compound **13**

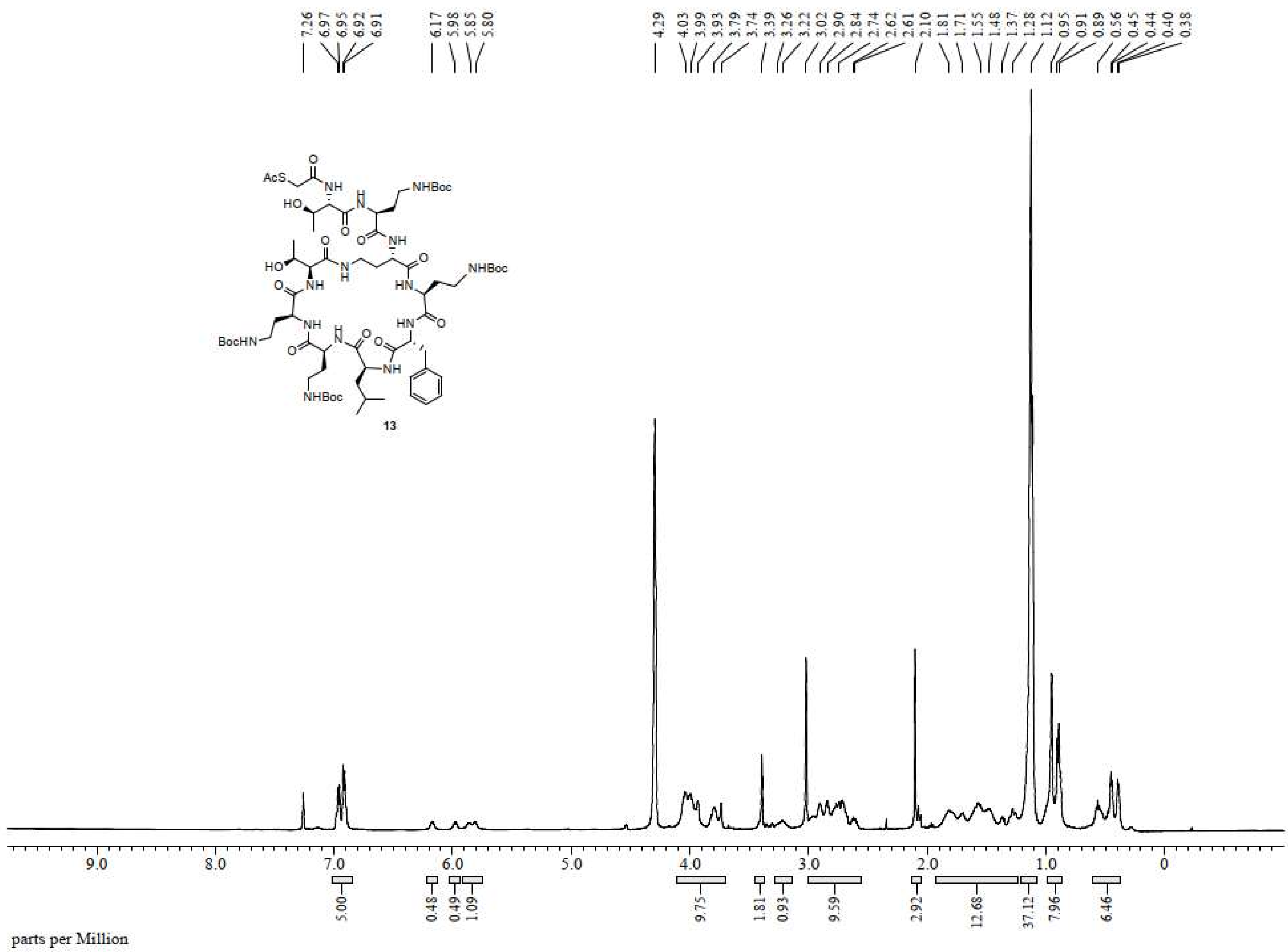

^{1}H NMR spectrum (500 MHz, D_2O) of compound **14**

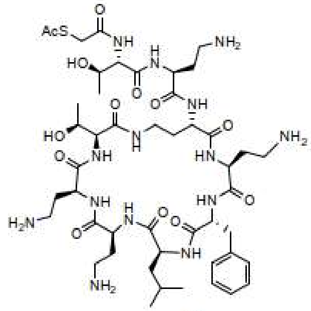

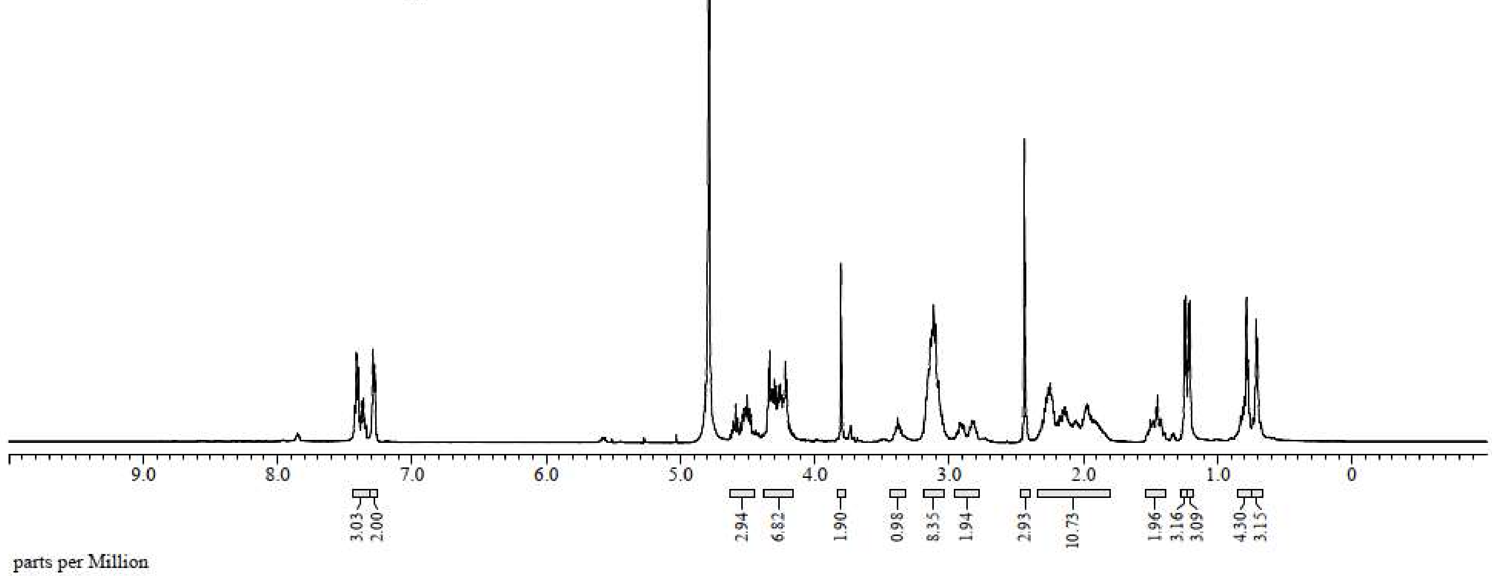

^{1}H NMR spectrum (500 MHz, D_2O) of compound **15**

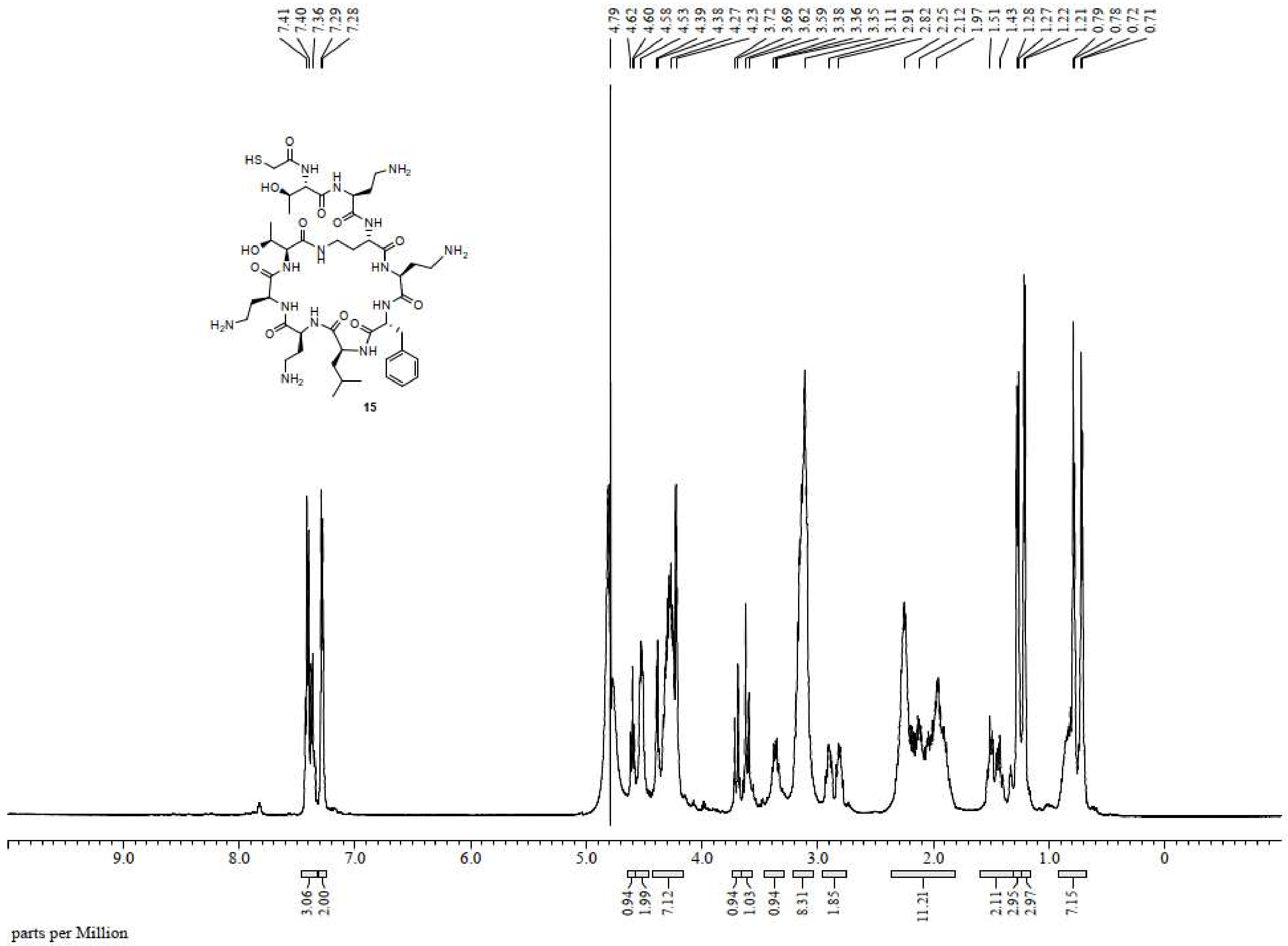

^1H NMR spectrum (400 MHz, 2:1 $CDCl_3$:CD_3OD) of compound **16**

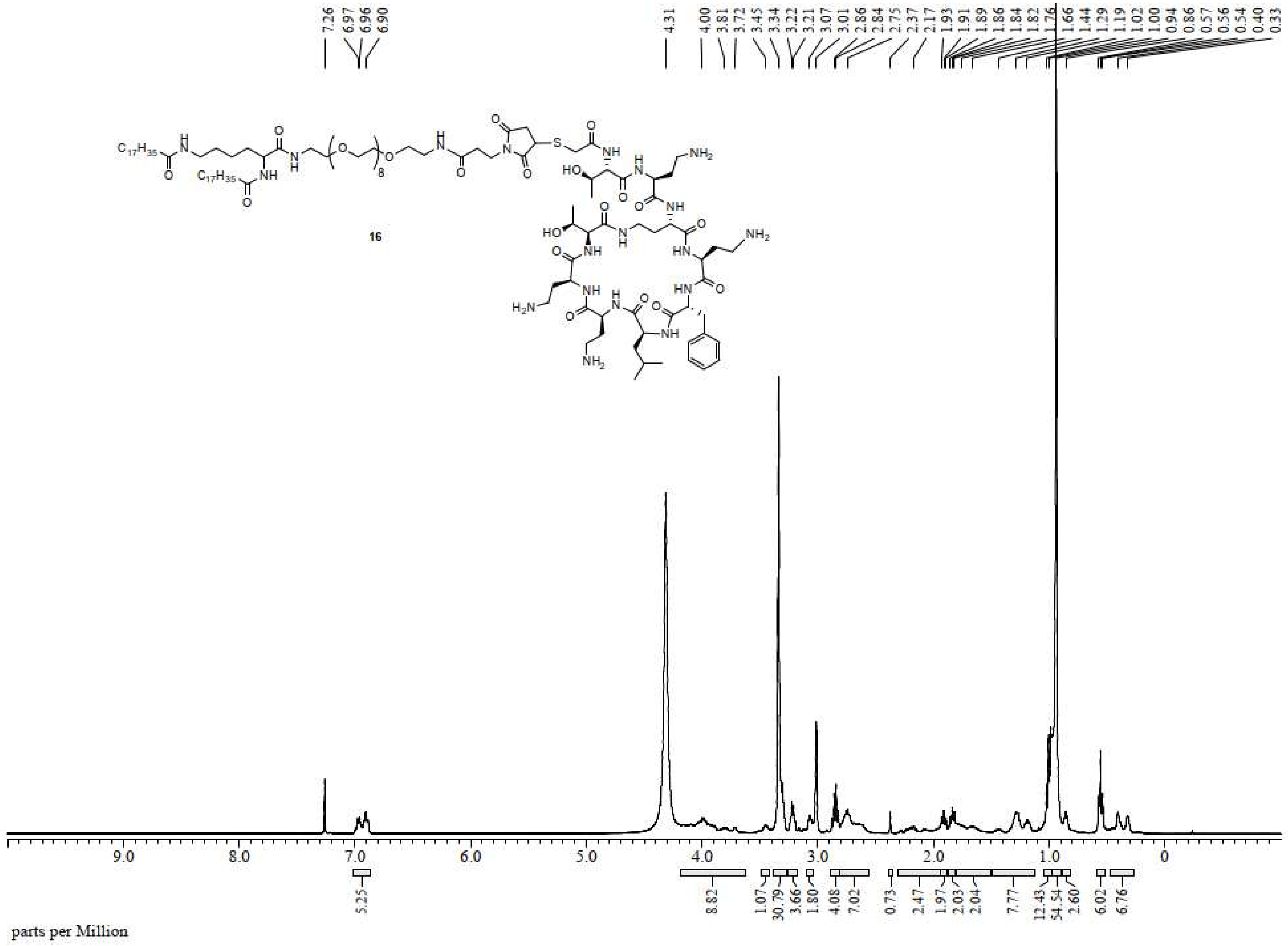

Table S1. DLS and zeta potential analysis of liposomes [a]

Liposome [b]	Day [c]	Average size (d.nm)	Zeta potential (mV)	Liposome [b]	Day [c]	Average size (d.nm)	Zeta potential (mV)
Phospholipids (1st exp) [d]	1	91.5 ± 1.2	-32.6 ± 1.4	Phospholipids (2nd exp) [d]	1	95.2 ± 0.8	-17.5 ± 0.5
	10	87.8 ± 0.3	-31.4 ± 1.3		10	121.2 ± 4.0	-20.5 ± 0.6
	17	92.0 ± 0.4	-33.2 ± 0.2		19	96.4 ± 0.8	-22.6 ± 0.8
	24	91.7 ± 0.2	-32.5 ± 0.7				
	31	88.7 ± 1.0	-33.8 ± 0.6				
Phospholipids/**2S-P1000** (1:1 w/w)	1	118.0 ± 0.6	-27.5 ± 3.6	Phospholipids/**2S-P1000** (3:1 w/w)	1	113.4 ± 0.6	-27.7 ± 0.9
	10	147.9 ± 1.7	-29.1 ± 0.7		10	114.1 ± 2.0	-23.5 ± 0.4
	17	157.0 ± 1.0	-27.2 ± 1.3		19	117.8 ± 1.0	-29.1 ± 1.0
	24	173.6 ± 2.0	-28.9 ± 1.4				
	31	171.8 ± 1.1	-26.7 ± 0.2				
Phospholipids/**2S-P3-Sia** (1:1 w/w)	1	125.6 ± 0.6	-43.2 ± 2.0	Phospholipids/**2S-P3-Sia** (3:1 w/w)	1	102.3 ± 0.6	-46.6 ± 0.6
	10	125.2 ± 1.1	-42.9 ± 1.1		10	129.8 ± 2.4	-49.8 ± 1.5

	17	128.5 ± 0.8	-42.9 ± 0.2		19	109.4 ± 0.8	-49.0 ± 0.8
	24	142.5 ± 1.2	-41.5 ± 0.8				
	31	140.3 ± 3.5	-45.9 ± 1.1				

[a] The average particle sizes and Zeta potentials were obtained from three measurements.

[b] Phospholipids are DSPC:DSPE:Chol (3:1:1, w/w) in 40 mM phosphate buffer (pH 7.2); each formulation contains 2.5% (w/w) **2S-P3-CF770**.

[c] The time after the formulation was stored at 4 ^{o}C.

[d] Liposomes from left and right columns were prepared separately (month apart) for respective *in vivo* biodistribution/retention experiments.

Table S2. The internalization of nanoparticle-FITC into target cells (median fluorescent intensity)

NP μg/mL	200	100	50	25	12.5	6.25
2S-P12-NH2	7174.5	7236.5	3690	1432.5	538	213.5
2S-P3-Sia	1761.5	1256.5	883	539	339	178.5

Figure S2 - First *in vivo* imaging experiment

- Phospholipids
- Phospholipids/2S-P1000 (1:1)
- Phospholipids/2S-Ps-Sia (1:1)

Figure S2 - In vivo Imaging IV (tail vein) with Phospholipids M1

Dorsal | **Ventral**

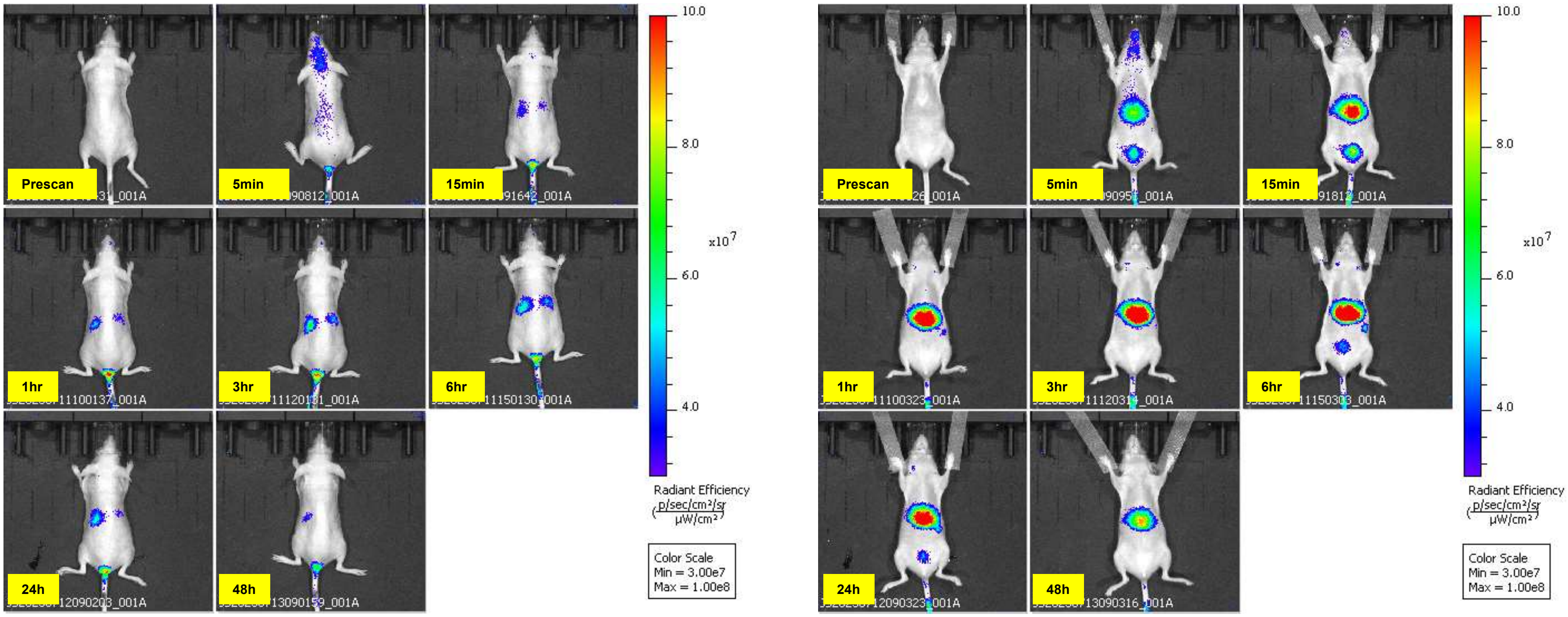

- High level of signals observed in the liver starting at 5 minutes and persisted at least for 48 hours
- Detectable signal in the bladder starting at 5 minutes.
- Detectable signals observed in the kidney at 15 minutes and peaked at 6 hours.

Figure S2 - *In vivo* Imaging IV (tail vein) with Phospholipids M2

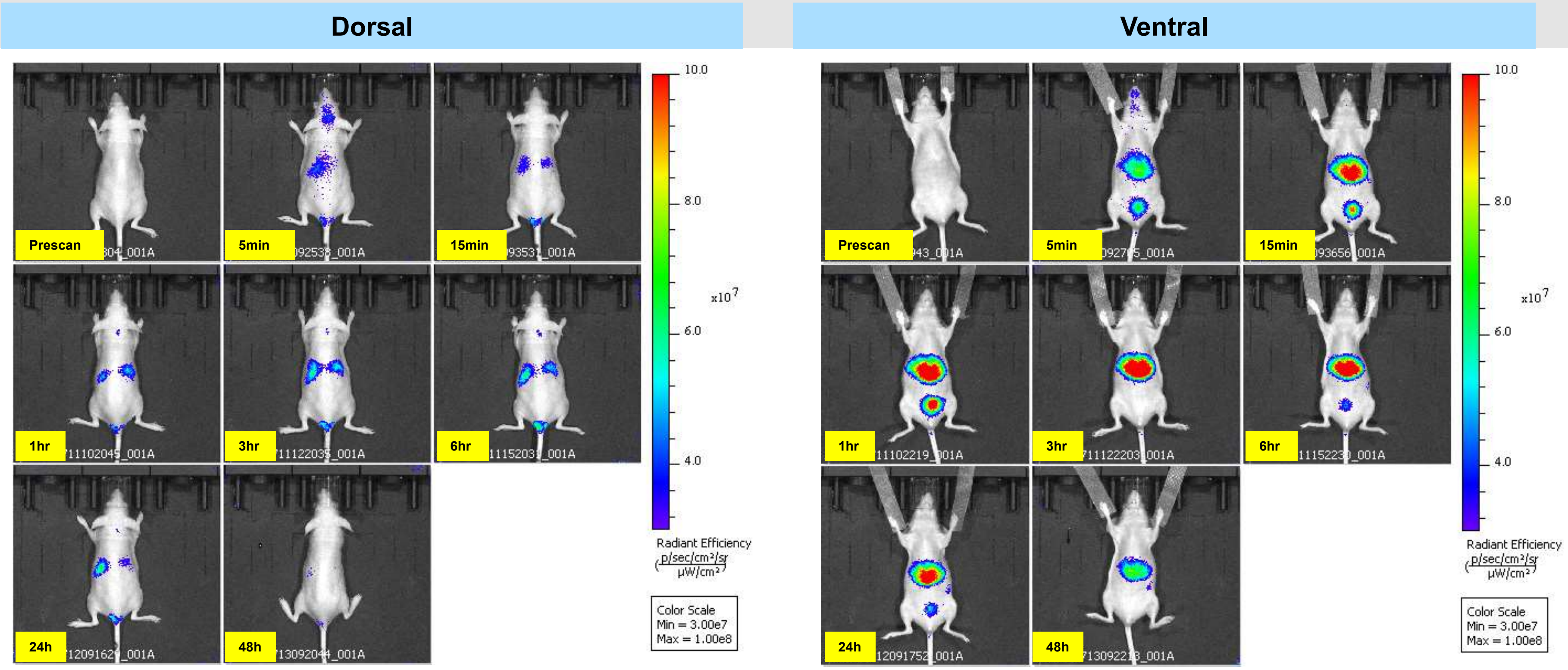

Figure S2 - *In vivo* Imaging IV (tail vein) with Phospholipids M3

Dorsal

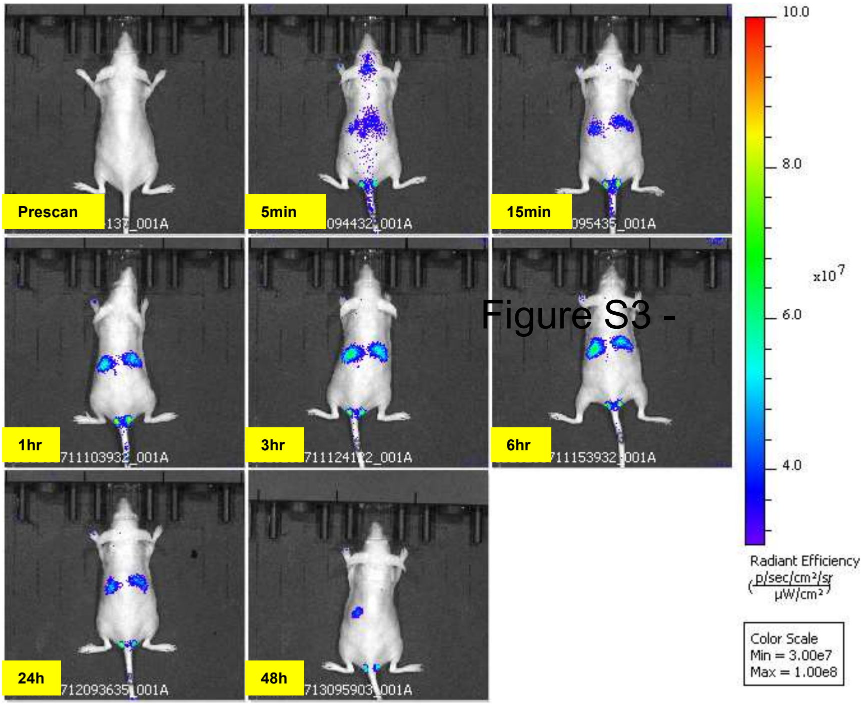

Ventral

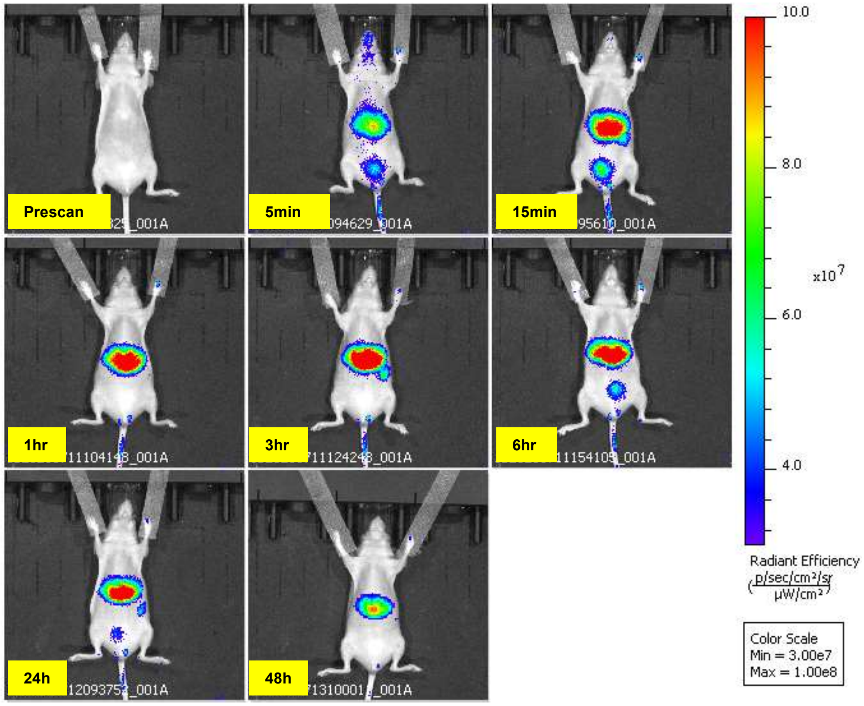

Figure S2 - *In vivo* Imaging IV (tail vein) with Phospholipids /2S-P1000 1:1 M4

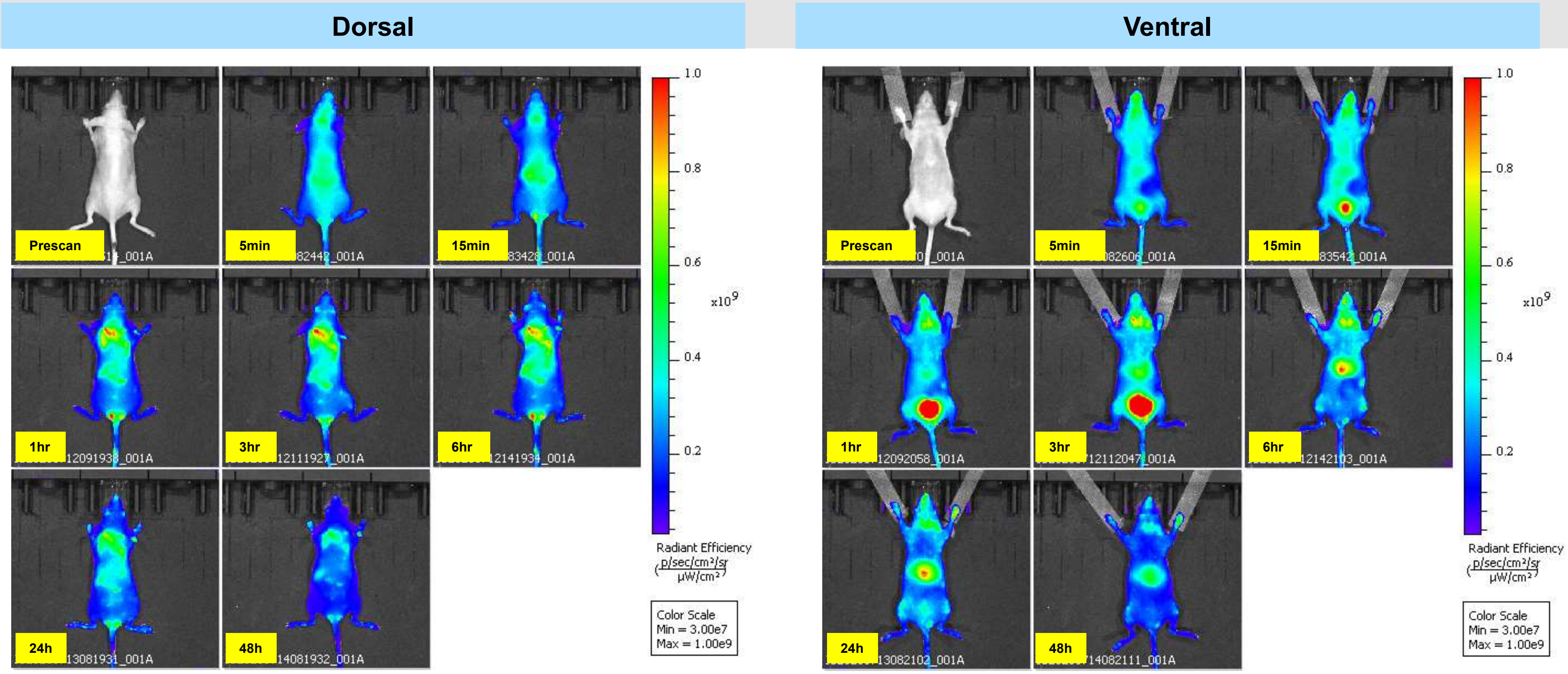

- Drug distributed to whole body within 5 minutes
- High level of signals were observed in the bladder starting at 15 minutes
- Higher signals observed in the liver at 6 and 24 hours then declined at 48 hours
- Body signals persisted at least for 48 hours

Figure S3 - *In vivo* Imaging IV (tail vein) with Phospholipids /2S-P1000 1:1 M5

Dorsal

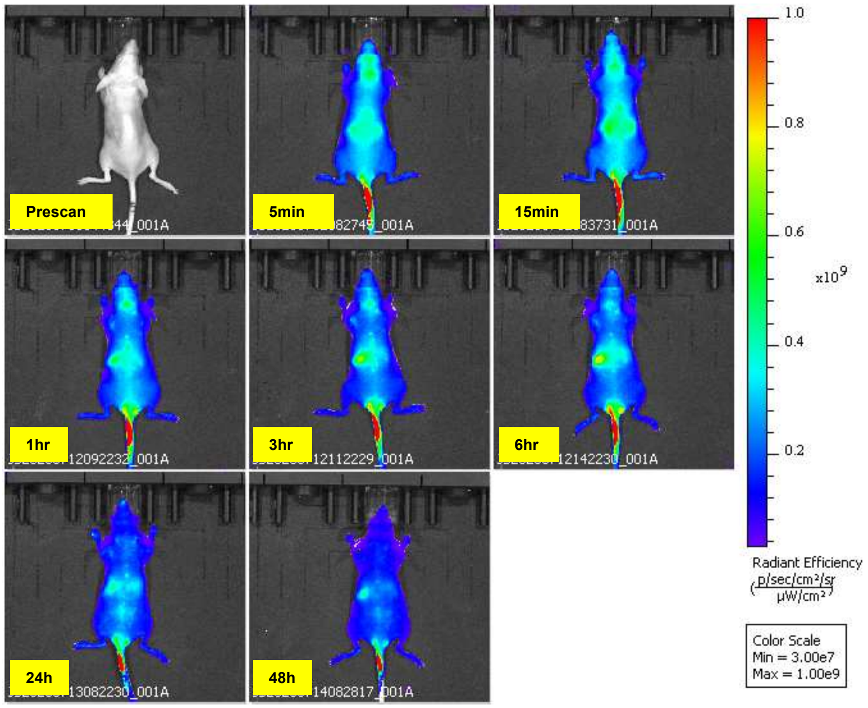

Ventral

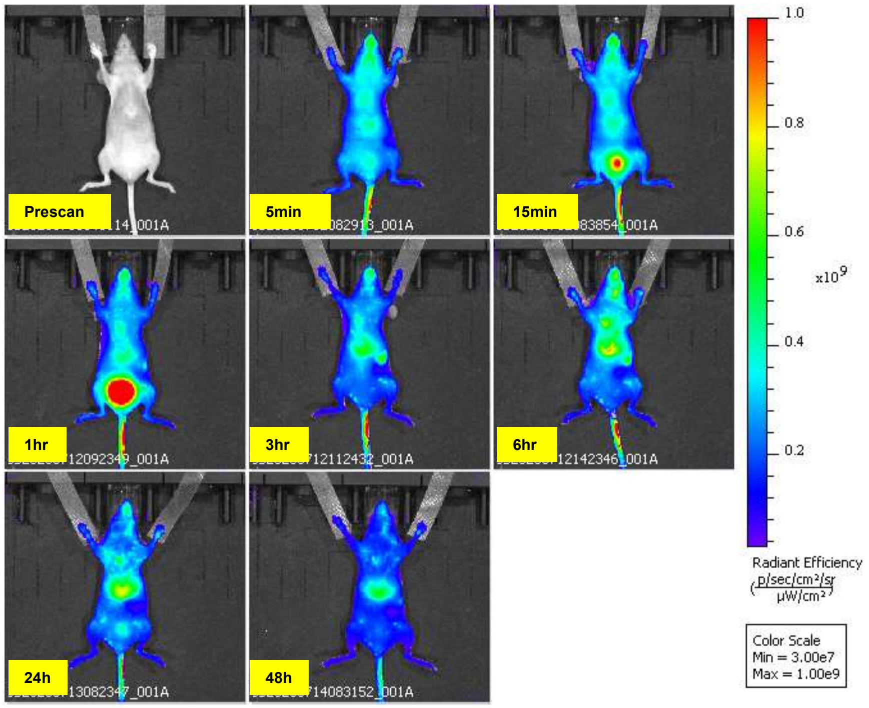

Figure S2 - *In vivo* Imaging IV (tail vein) with Phospholipids /2S-P1000 1:1 M6

Dorsal

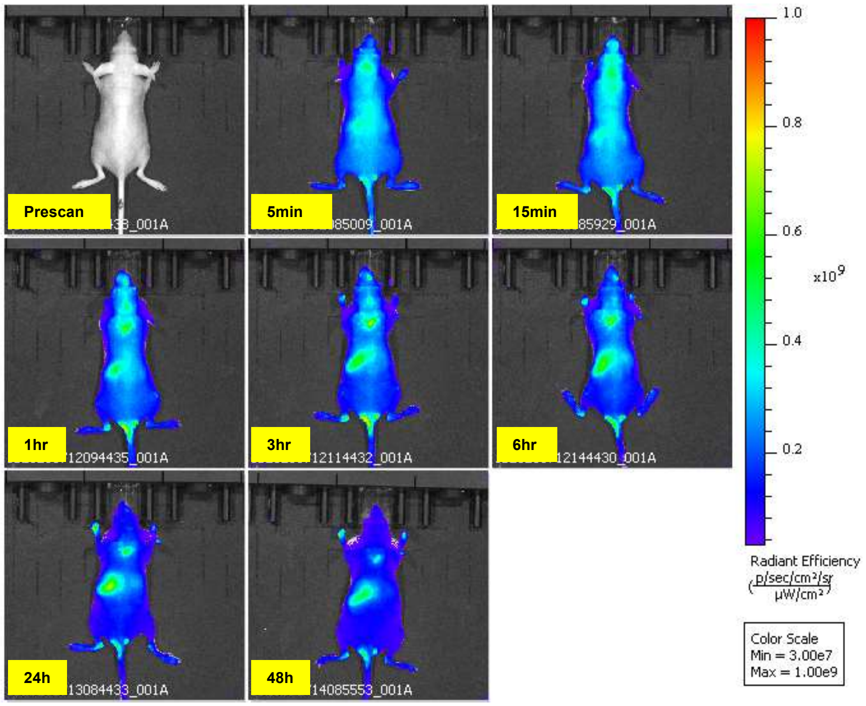

Ventral

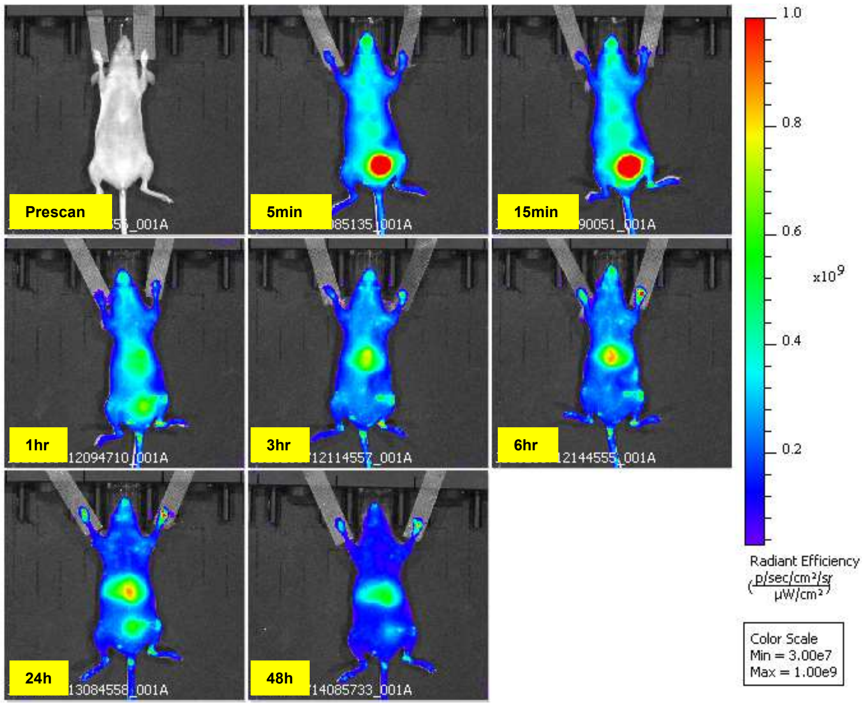

Figure S2 - *In vivo* Imaging IV (tail vein) with Phospholipids /2S-Sia 1:1 M7

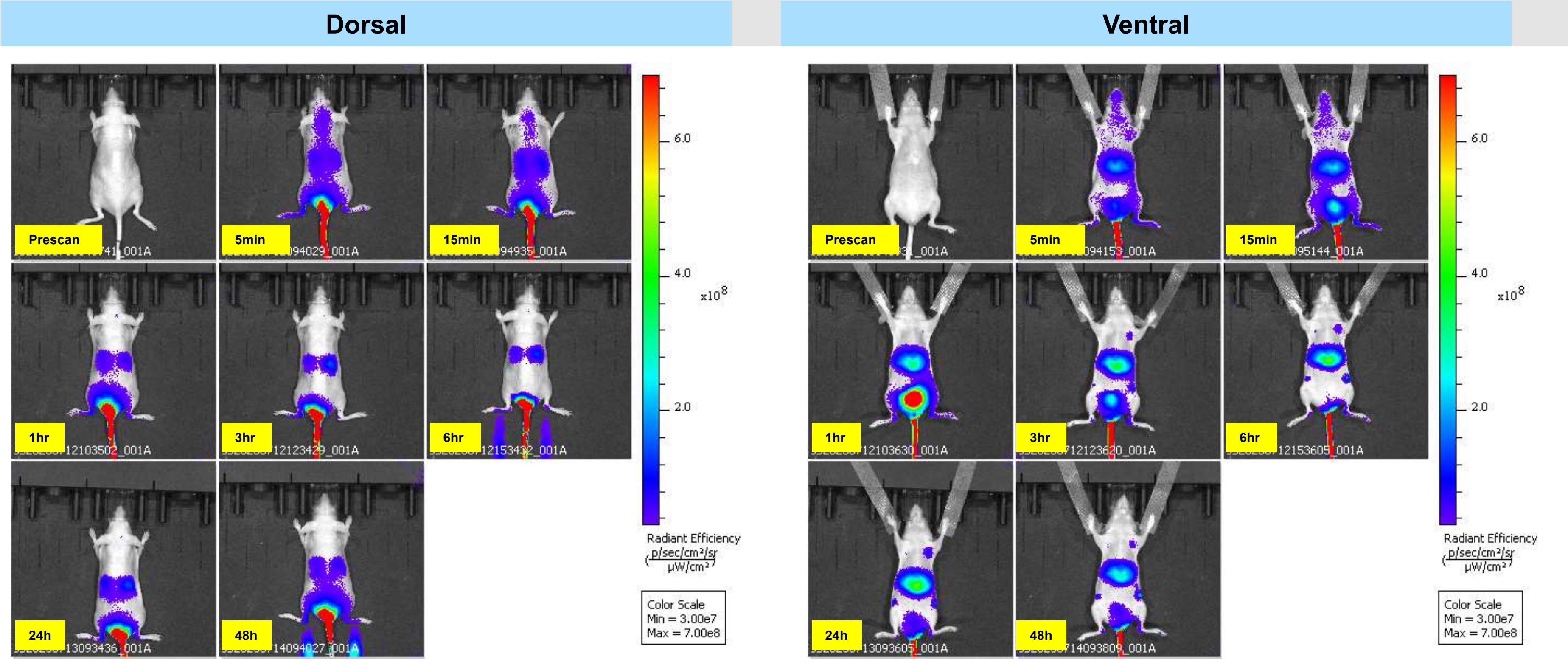

- NOTE: Large signals in tail vein suggesting failed IV injection.

Figure S3 - *In vivo* Imaging IV (tail vein) with Phospholipids /2S-Sia 1:1 M8

Dorsal | **Ventral**

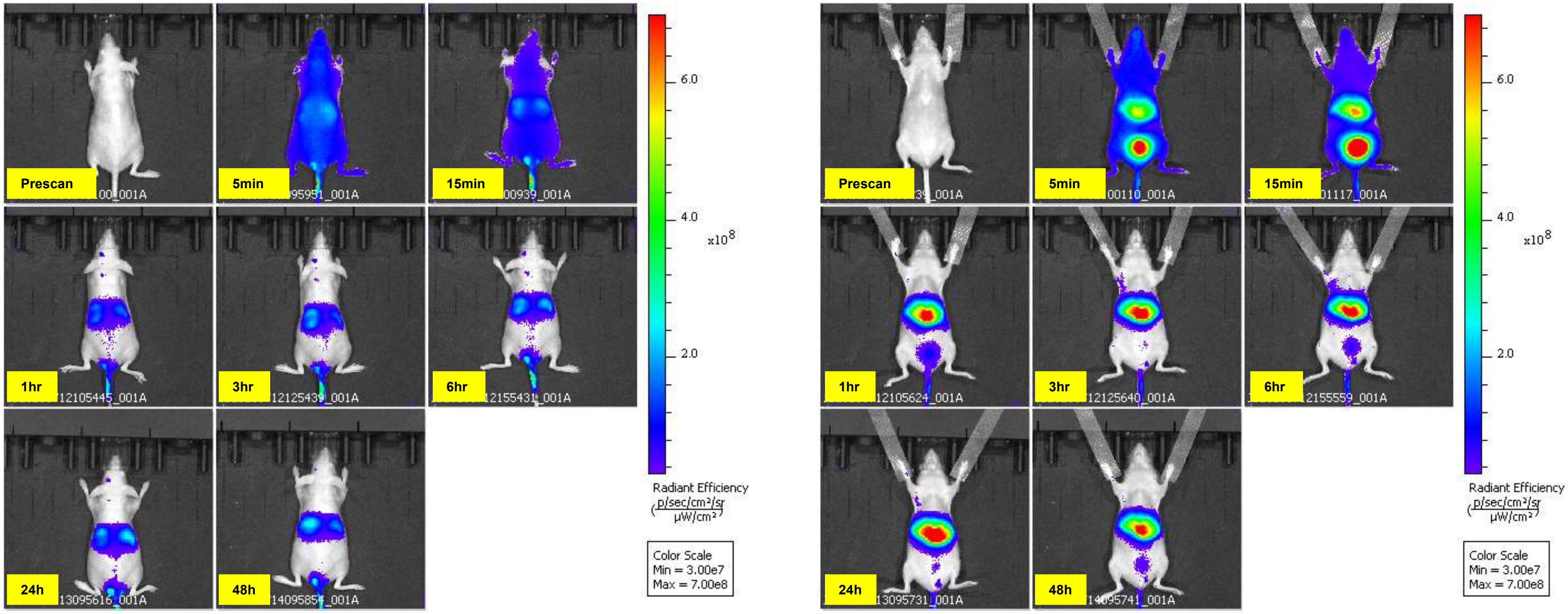

- Higher level of signals were observed in the kidney and liver starting at 5 minutes.
- High signal in liver persisted for up to 48hrs
- Signals were observed in bladder starting at 5 minutes

Figure S2 - *In vivo* Imaging IV (tail vein) with Phospholipids /2S-Sia 1:1 M9

Dorsal

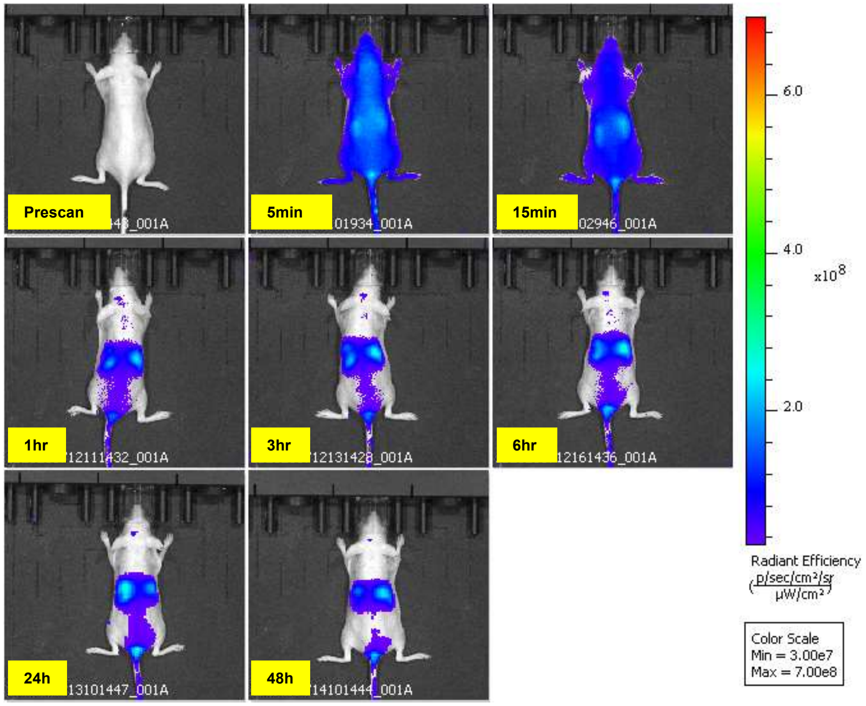

Ventral

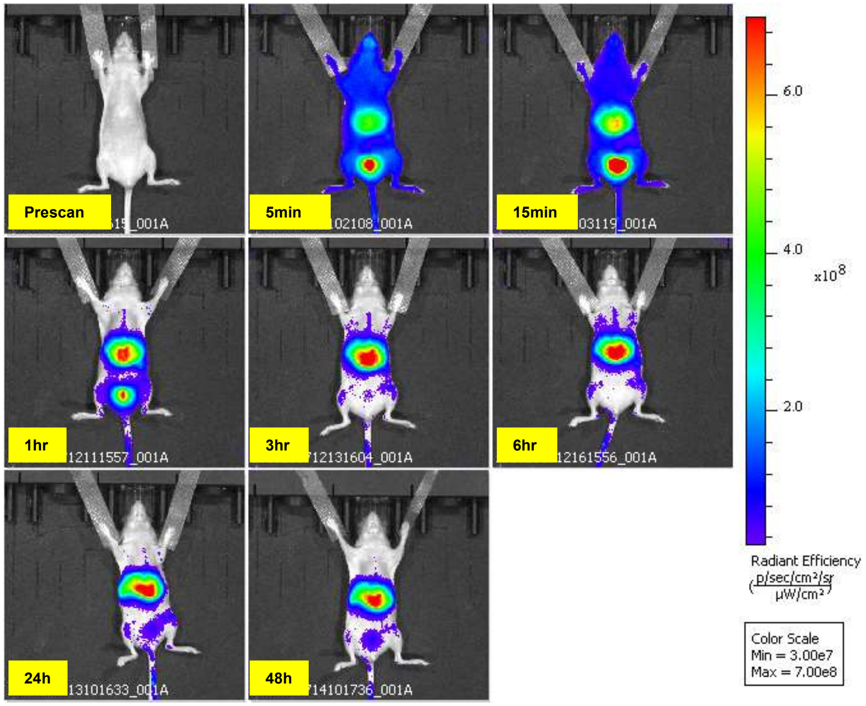

Figure S3 - *Ex vivo* Imaging IV (tail vein) with Phospholipids

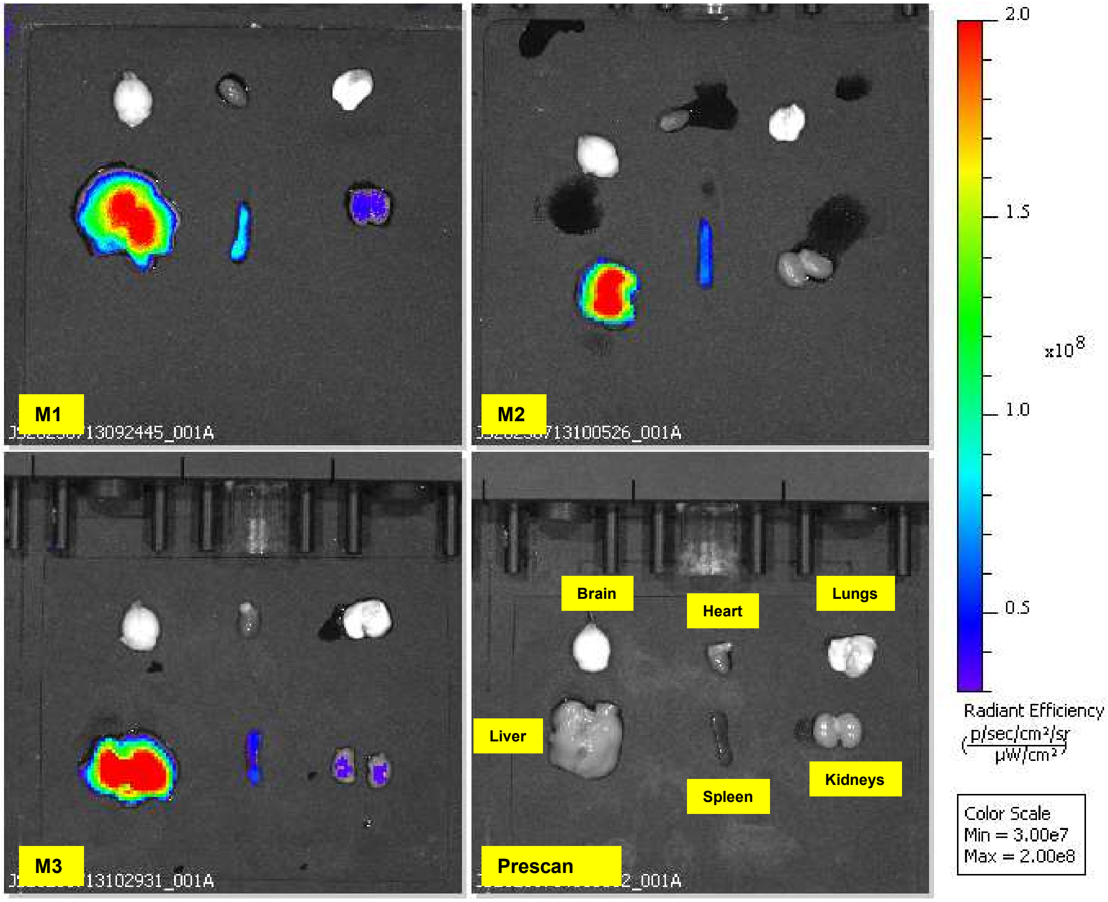

Quantification of *ex vivo* Imaging IV (tail vein) inj. of Group 1 Phospholipids alone: Total radiant efficiency (avg of 3 mice, mean +/- SD)

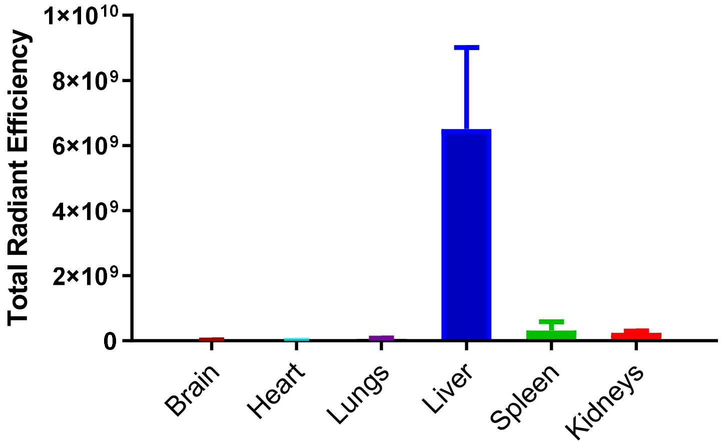

- Highest signals observed in the liver.
- Lower signals observed in spleen and kidneys.

Figure S3 - *Ex Vivo* Imaging IV (tail vein) with Phospholipids /2S-P1000 1:1

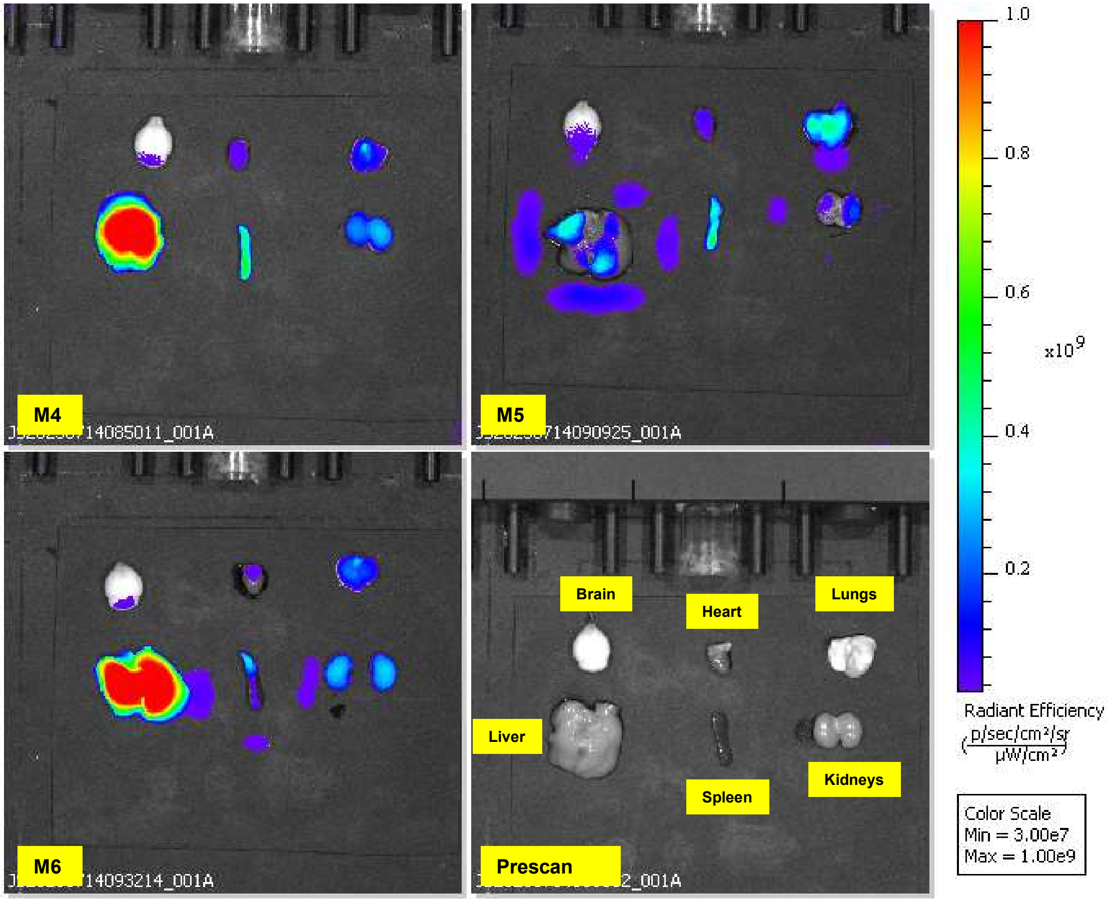

Quantification of *ex vivo* Imaging IV (tail vein) inj. of Group 2 Phospholipids/2S-P1000 1:1: Total radiant efficiency (avg of 3 mice, mean +/- SD)

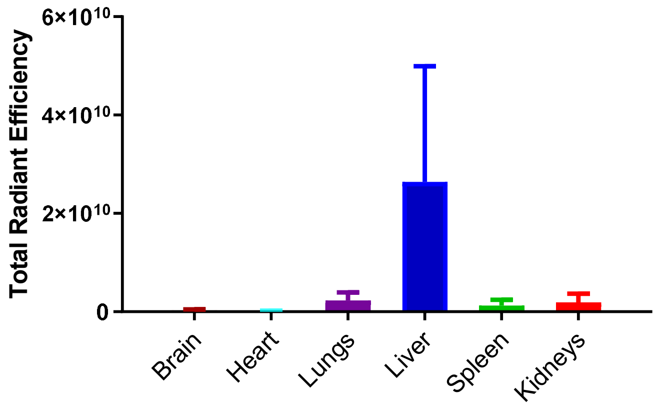

- Highest signals observed in the liver.
- Lower signals observed in spleen, kidneys and lung.

Figure S3 - *Ex Vivo* Imaging IV (tail vein) inj. of Phospholipids /2S-Sia 1:1

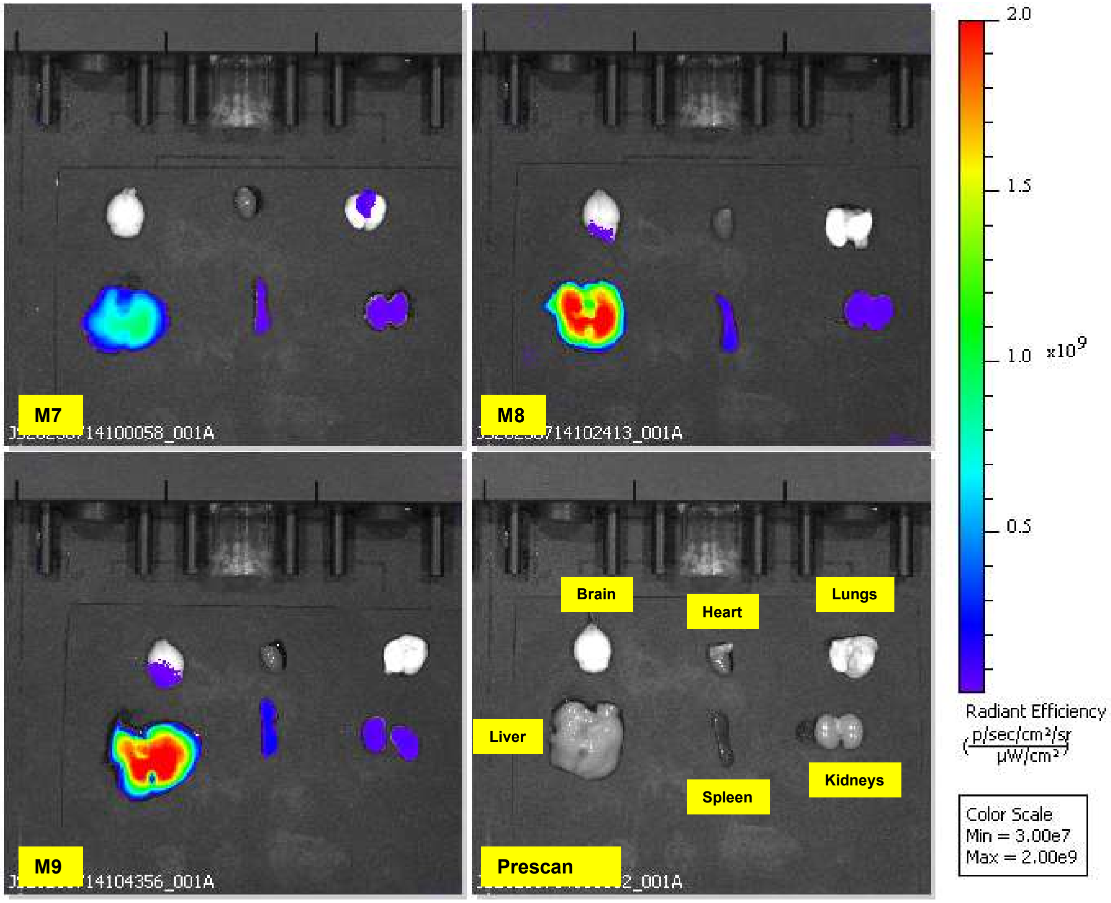

Quantification of *ex vivo* Imaging IV (tail vein) inj. of Group 2 Phospholipids/2S-Sia 1:1: Total radiant efficiency (avg of 3 mice, mean +/- SD)

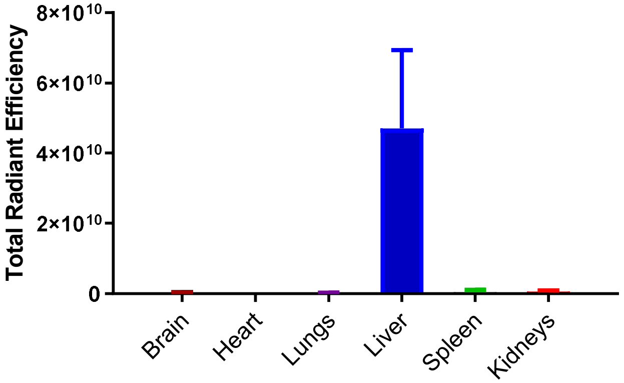

- Highest signals observed in the liver.

Figure S4 - Second *in vivo* imaging experiment

- Phospholipids
- Phospholipids/2S-P1000 (3:1)
- Phospholipids/2S-Ps-Sia (3:1)

Figure S4 - *In vivo* Imaging IV (tail vein) with Phospholipids M1

Dorsal

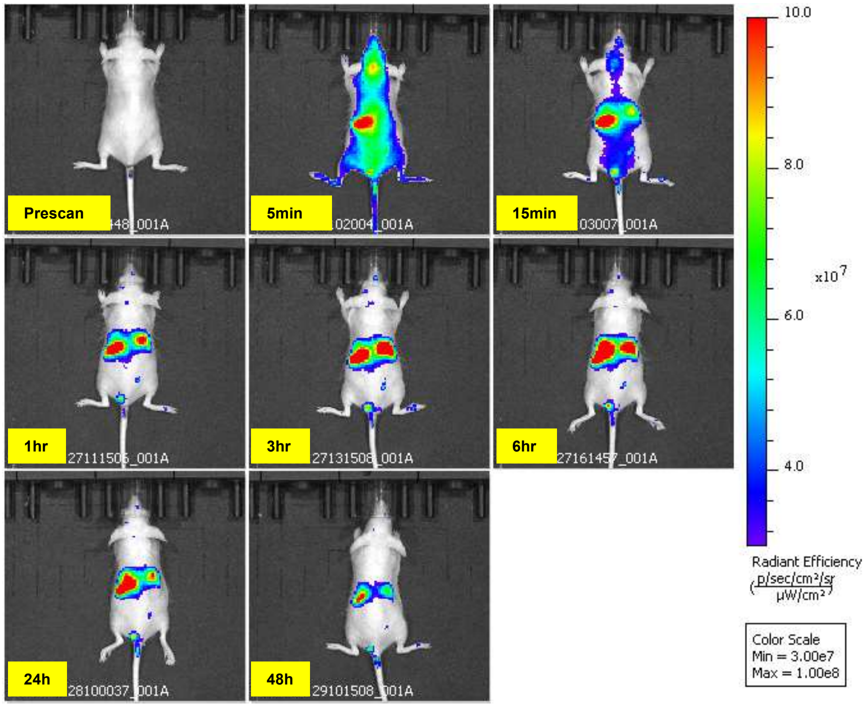

Ventral

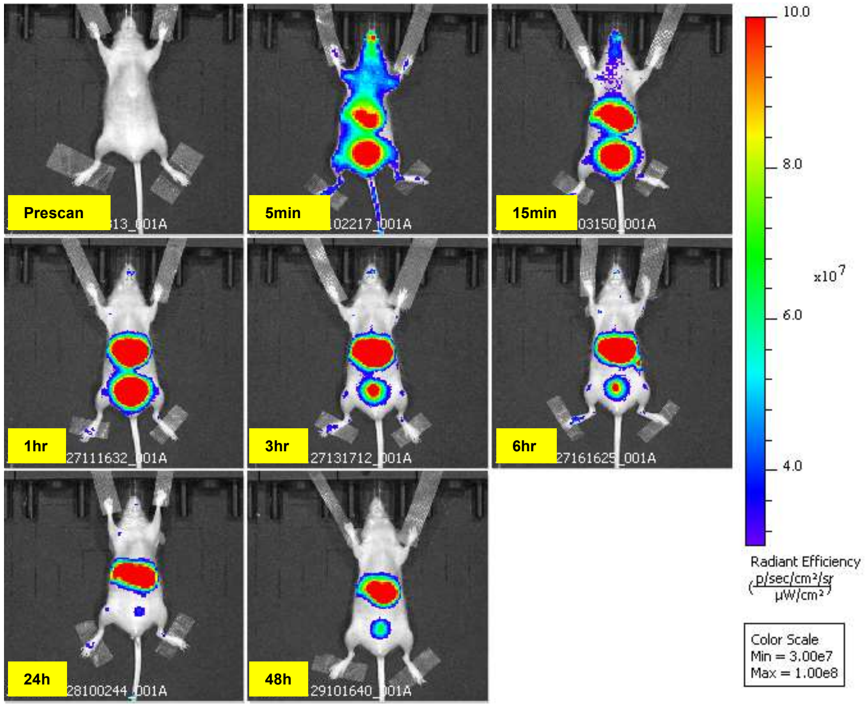

- High level of signals observed in the liver and kidney starting at 5 minutes and persisted at least for 48 hours
- Higher signal in the bladder starting at 5 minutes.

Figure S4 - *In vivo* Imaging IV (tail vein) with Phospholipids M2

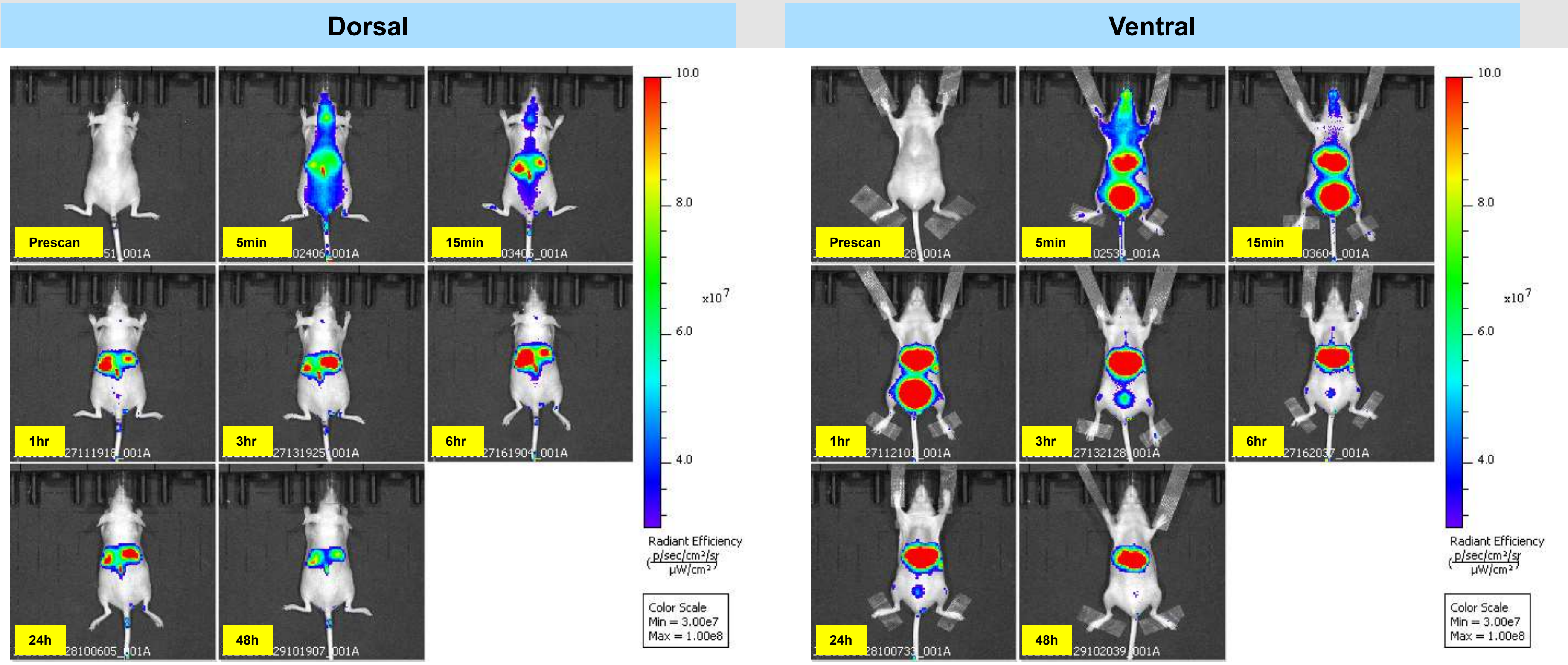

Figure S4 - *In vivo* Imaging IV (tail vein) with Phospholipids M3

Dorsal

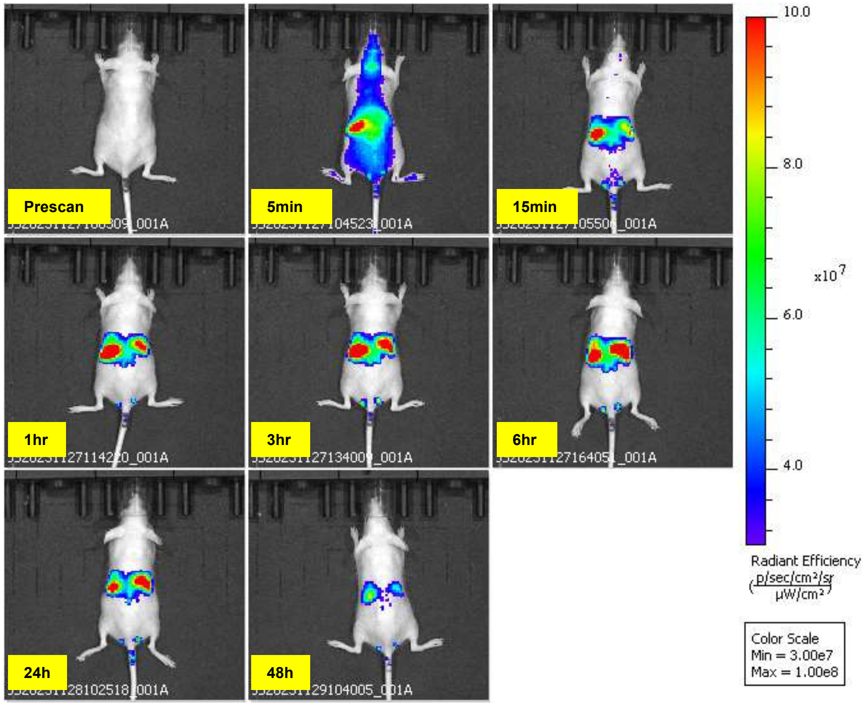

Ventral

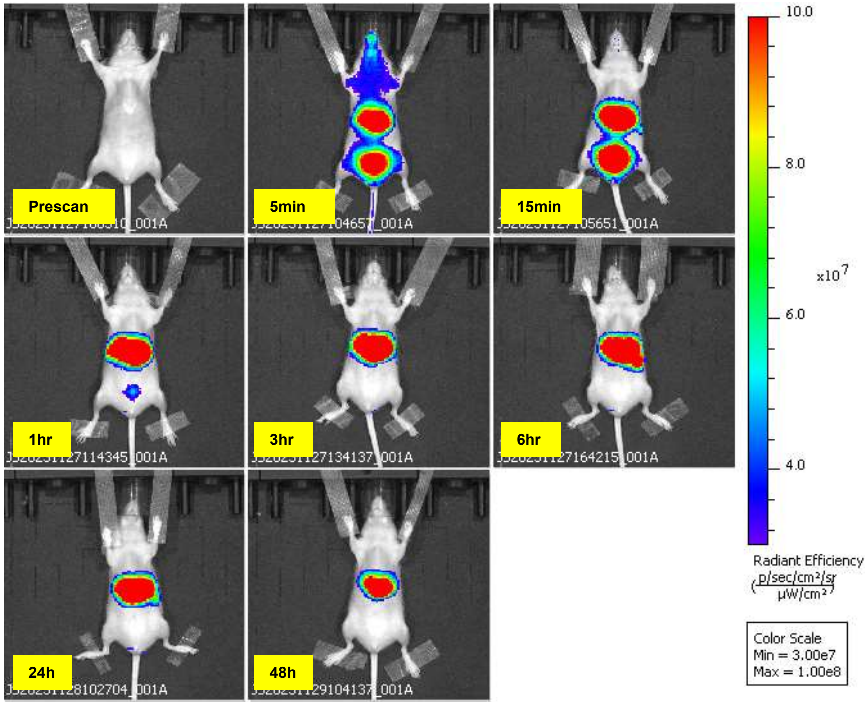

Figure S4 - *In vivo* Imaging IV (tail vein) with Phospholipids/2S-P1000 3:1 M1

Dorsal | **Ventral**

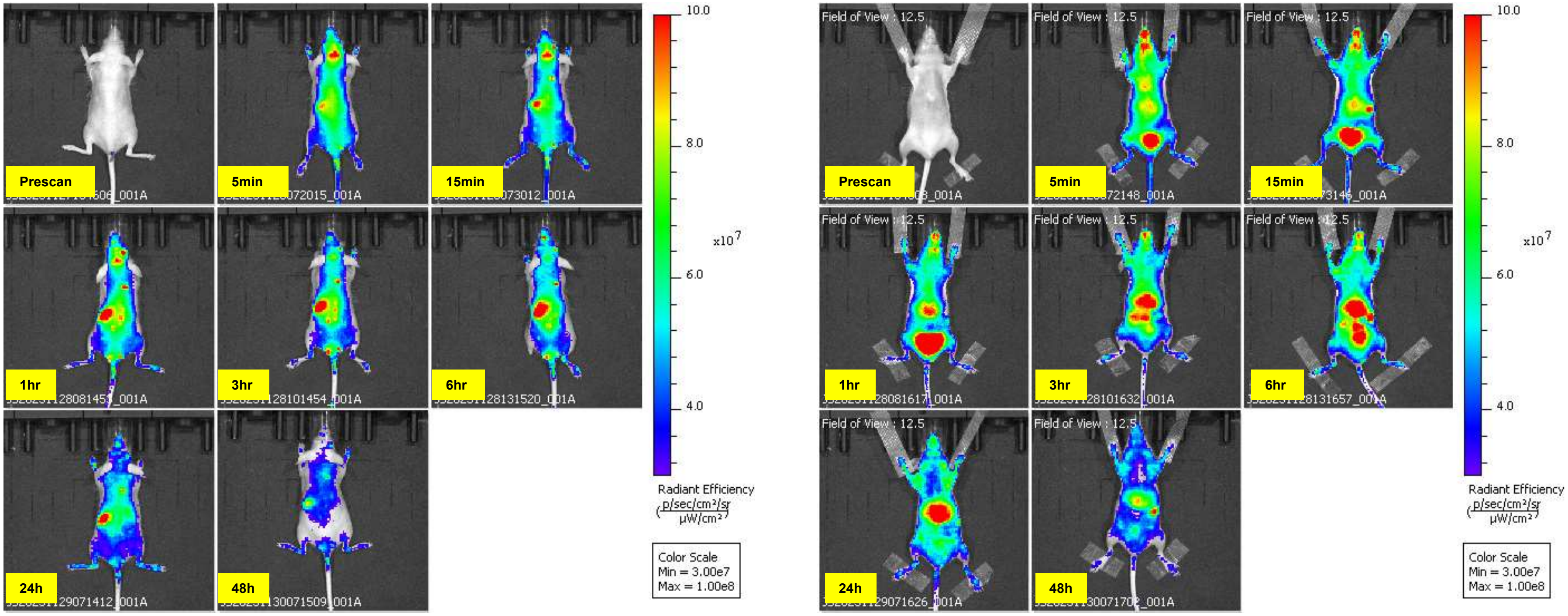

- Drug distributed to whole body within 5 minutes
- High level of signals were observed in the mouth and neck area starting at 5 minutes then decline with time
- High signals were observed in the kidney and persisted until 48 hours.
- Higher signals observed in the liver at 6 and 24 hours then declined at 48 hours
- Body signals persisted at least for 48 hours

Figure S4 - *In vivo* Imaging IV (tail vein) with Phospholipids/2S-P1000 3:1 M2

Dorsal | Ventral

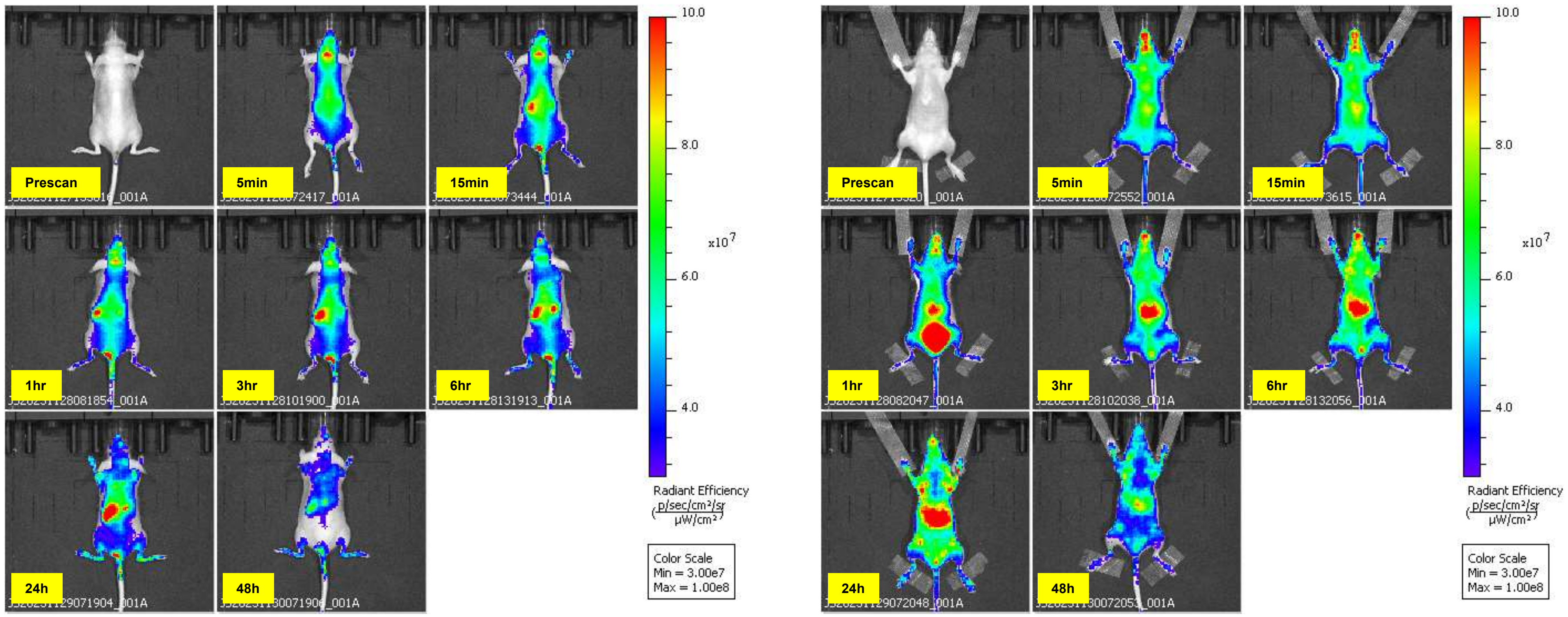

Figure S4 - *In vivo* Imaging IV (tail vein) with Phospholipids/2S-P1000 3:1 M3

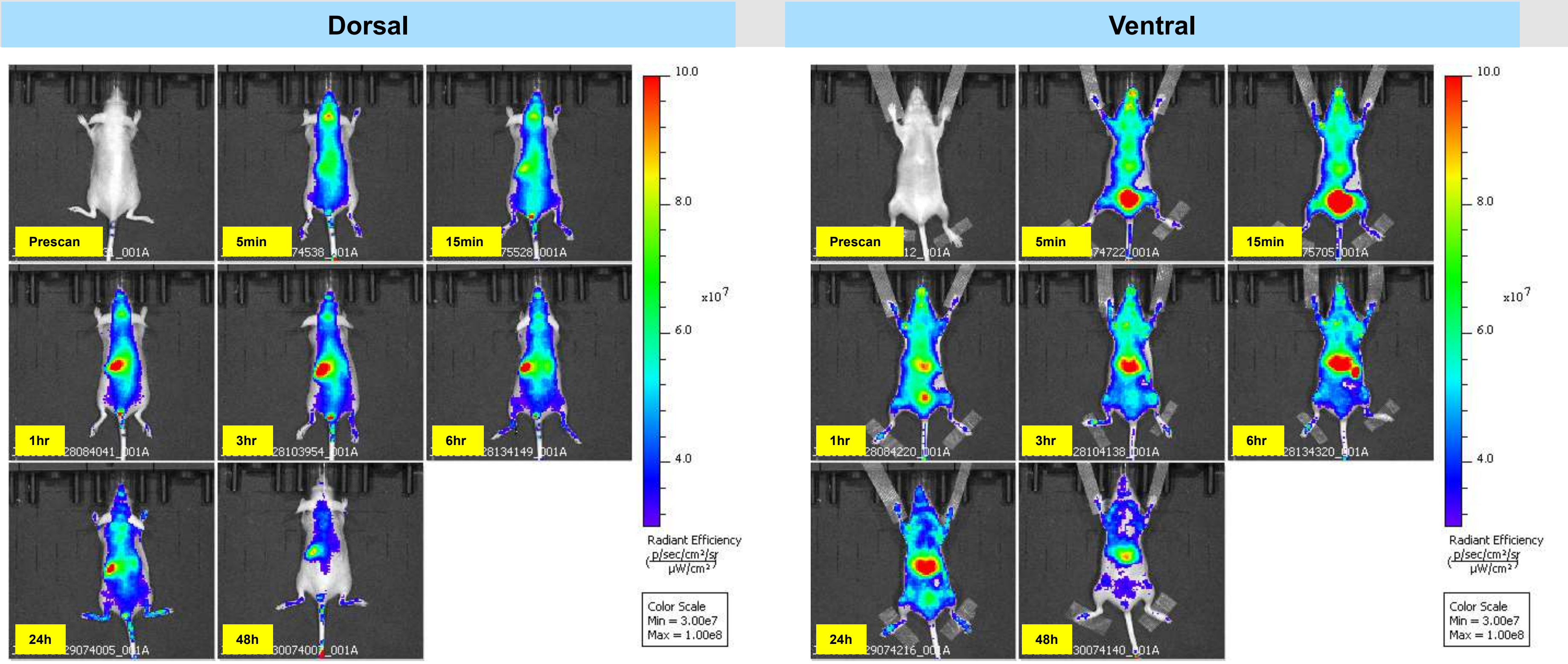

Figure S4 - *In vivo* Imaging IV (tail vein) with Phospholipids/2S-P3-Sia 3:1 M1

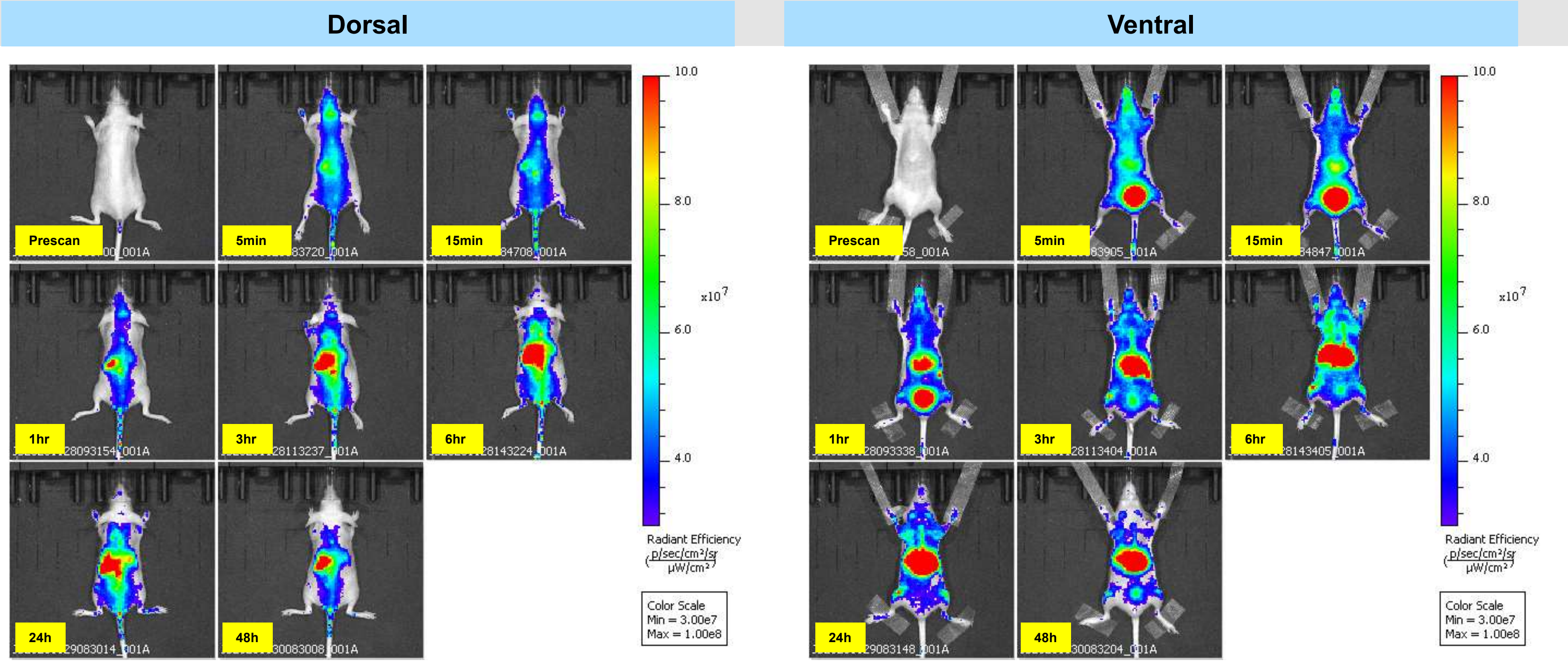

- Drug distributed to centre of the body within 5 minutes
- High level of signals were observed in the mouth and neck area starting at 5 minutes then decline with time
- High signals were observed in the kidney and persisted until 48 hours.
- Higher signals observed in the liver at 6 and 24 hours and persisted until 48 hours
- Body signals persisted at least for 48 hours

Figure S4 - *In vivo* Imaging IV (tail vein) with Phospholipids/2S-P3-Sia 3:1 M2

Dorsal | **Ventral**

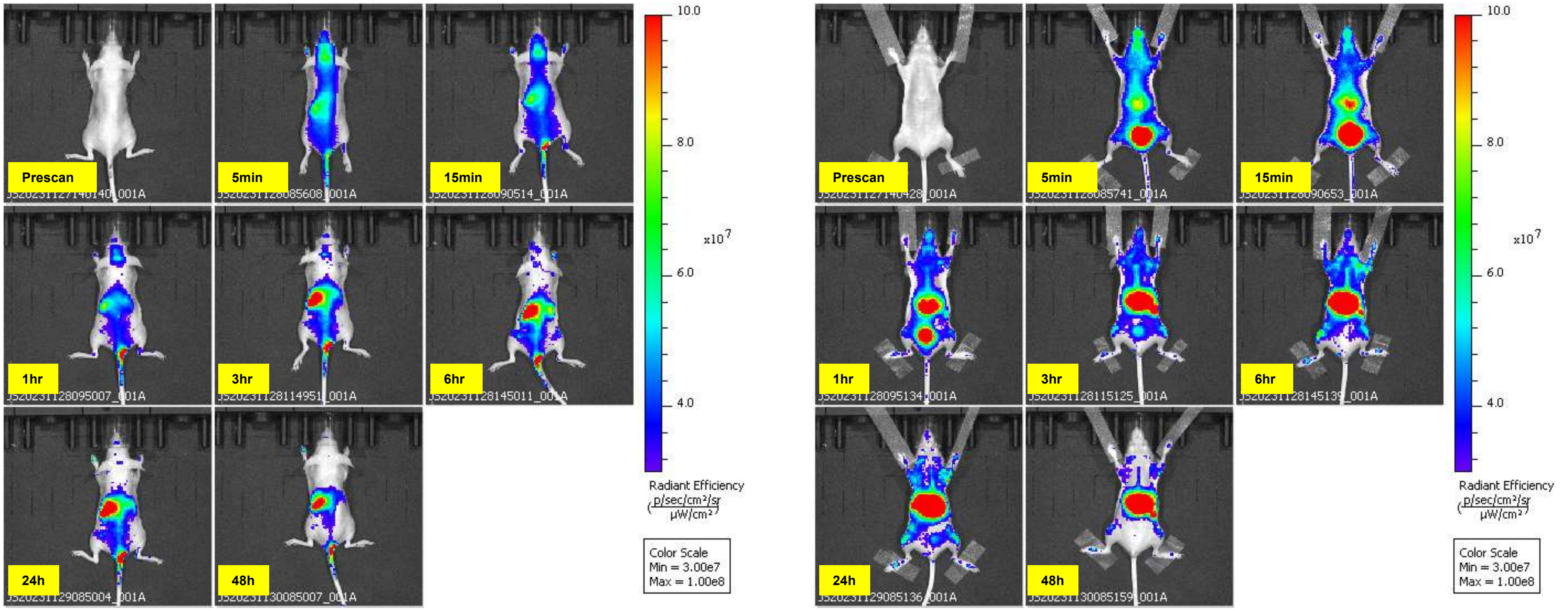

- Higher level of signals were observed in the kidney and liver starting at 5 minutes.
- High signal in liver persisted for up to 48hrs
- Signals were observed in bladder starting at 5 minutes

Figure S4 - *In vivo* Imaging IV (tail vein) with Phospholipids/2S-P3-Sia 3:1 M3

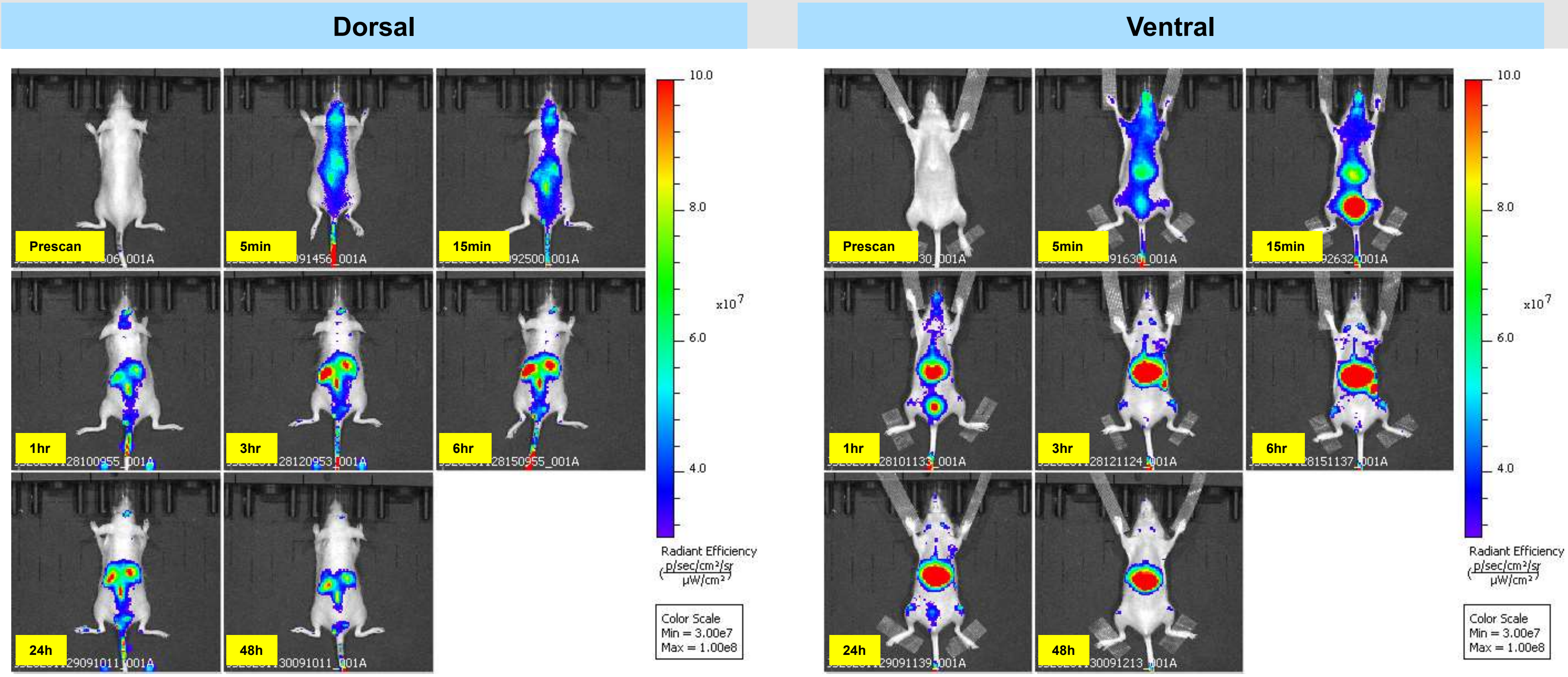

Figure S5 - *Ex vivo* Imaging IV (tail vein) with Phospholipids

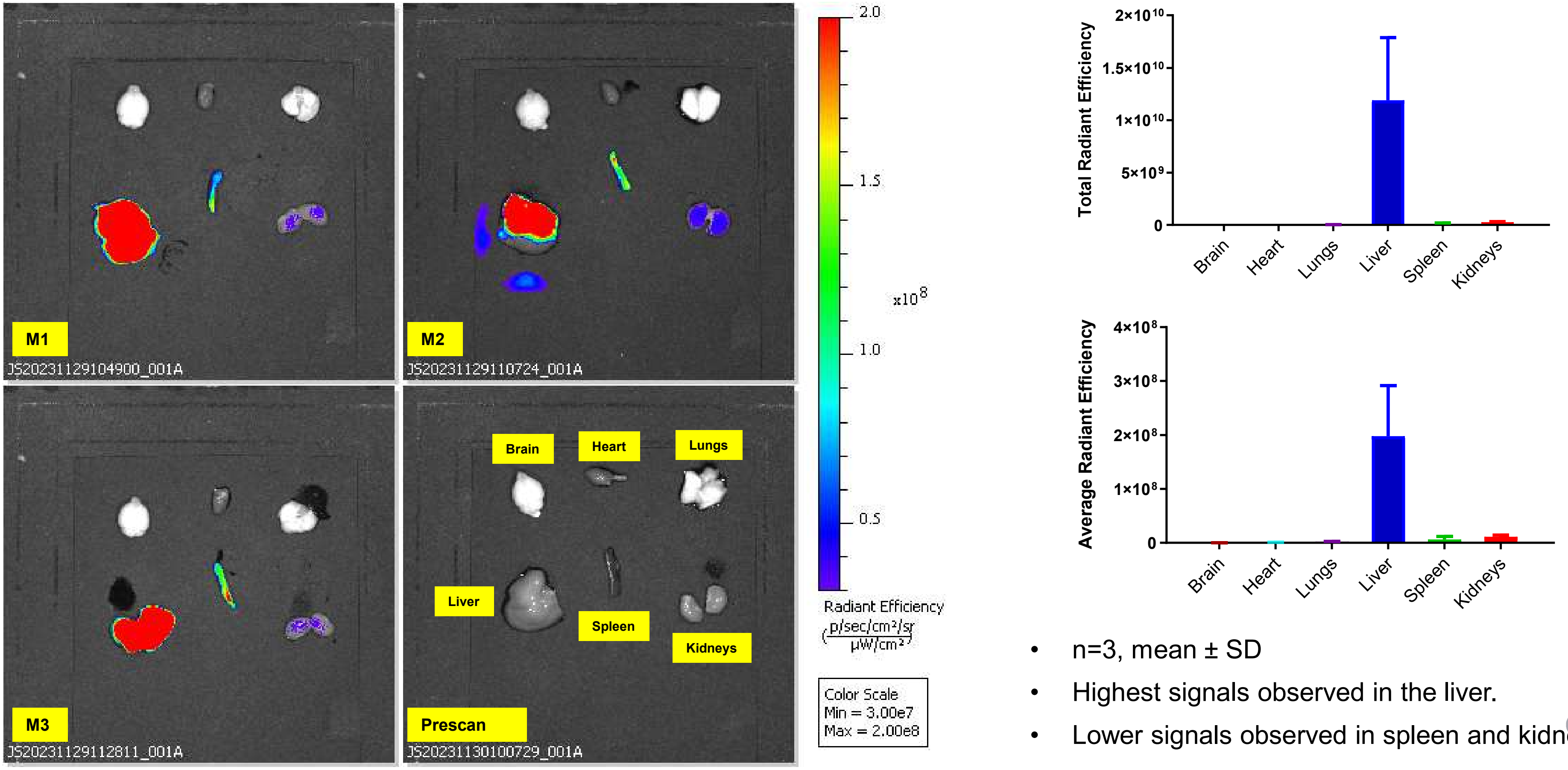

- n=3, mean ± SD
- Highest signals observed in the liver.
- Lower signals observed in spleen and kidneys.

Figure S5 - *Ex Vivo* Imaging IV (tail vein) with Phospholipids/2S-P1000 3:1 (Drug 3B)

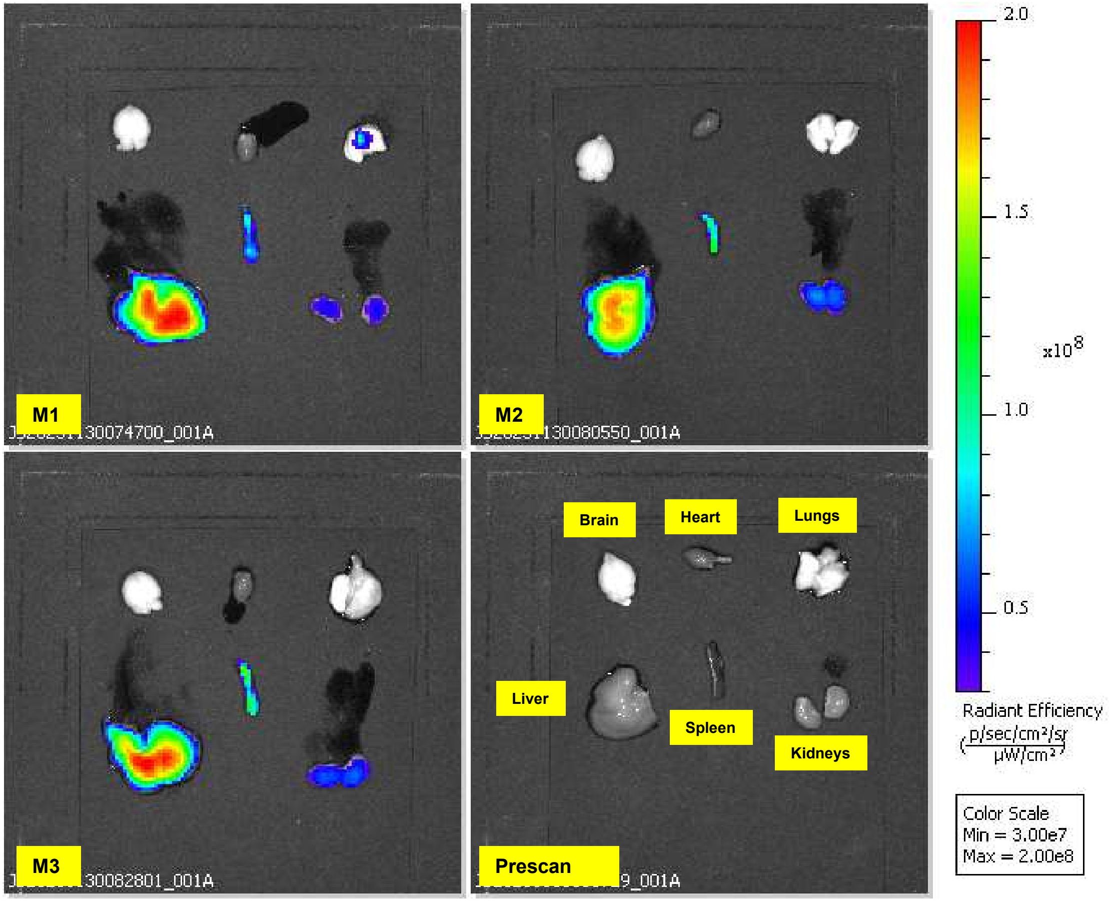

n=3, mean ± SD

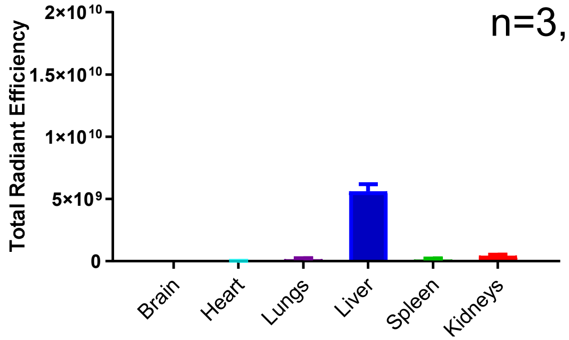

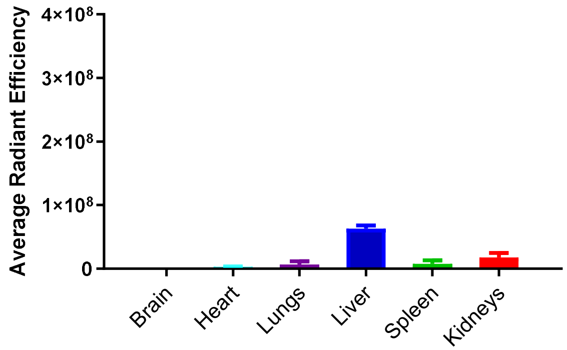

- Highest signals observed in the liver.
- Lower signals in lung, kidney and spleen.

Figure S5 - *Ex Vivo* Imaging IV (tail vein) with Phospholipids/2S-P3-Sia 3:1

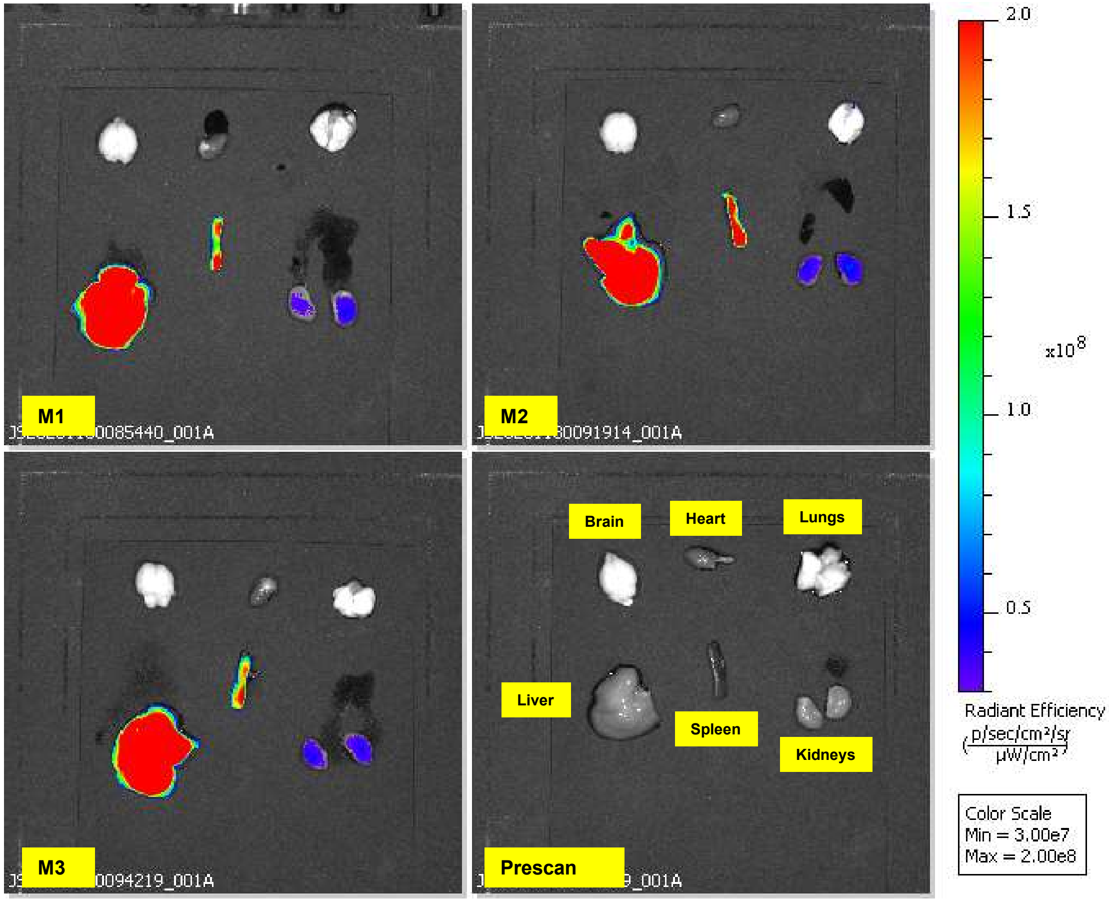

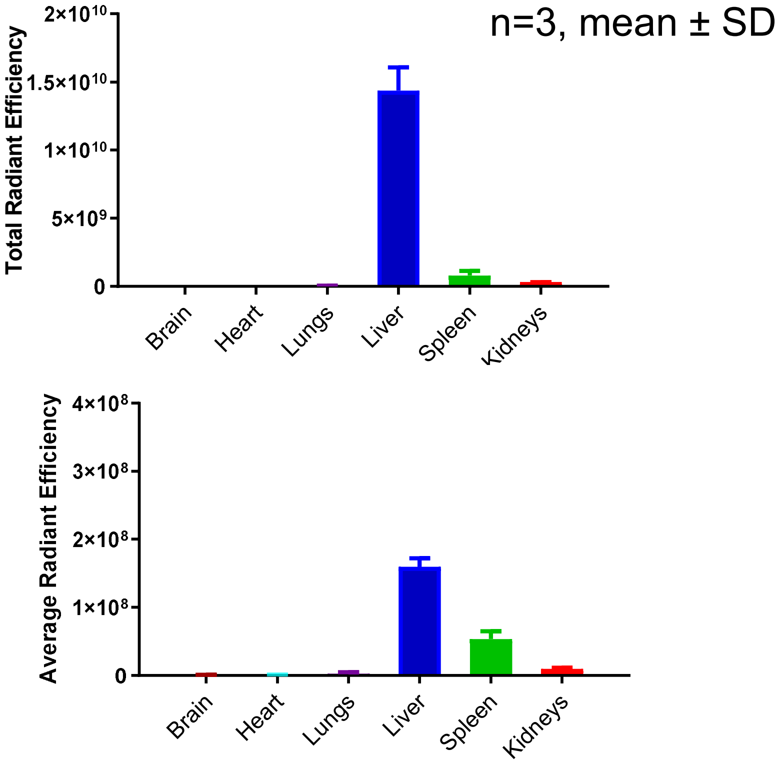

- Highest signals observed in the liver.
- Lower signals in lung, kidney and spleen.
